# Synthesis and Evaluation of Hypoglycemic Activity of Structural Isomers of ((Benzyloxy)phenyl)propanoic Acid Bearing an Aminobornyl Moiety

**DOI:** 10.3390/ijms24098022

**Published:** 2023-04-28

**Authors:** Sergey O. Kuranov, Darya A. Pon`kina, Yulia V. Meshkova, Mariya K. Marenina, Mikhail V. Khvostov, Olga A. Luzina, Tatiana G. Tolstikova, Nariman F. Salakhutdinov

**Affiliations:** N. N. Vorozhtsov Novosibirsk Institute of Organic Chemistry, Siberian Branch of the Russian Academy of Sciences, 9, Akademika Lavrentieva Ave., 630090 Novosibirsk, Russia; skuranov@nioch.nsc.ru (S.O.K.); fominamk@gmail.com (M.K.M.); khvostov@nioch.nsc.ru (M.V.K.); tolstiktg@nioch.nsc.ru (T.G.T.);

**Keywords:** GPR40, FFAR1, T2DM, diabetes, nature product

## Abstract

Free fatty acid receptor-1 (FFAR1) agonists are promising candidates for therapy of type 2 diabetes because of their ability to normalize blood sugar levels during hyperglycemia without the risk of hypoglycemia. Previously, we synthesized compound **QS-528**, a FFA1 receptor agonist with a hypoglycemic effect in C57BL/6NCrl mice. In the present work, structural analogs of **QS-528** based on (hydroxyphenyl)propanoic acid bearing a bornyl fragment in its structure were synthesized. The seven novel compounds synthesized were structural isomers of compound **QS-528**, varying the positions of the substituents in the aromatic fragments as well as the configuration of the asymmetric center in the bornyl moiety. The studied compounds were shown to have the ability to activate FFAR1 at a concentration of 10 μM. The cytotoxicity of the compounds as well as their effect on glucose uptake in HepG2 cells were studied. The synthesized compounds were found to increase glucose uptake by cells and have no cytotoxic effect. Two compounds, based on the meta-substituted phenylpropanoic acid, 3-(3-(4-(((1R,2R,4R)-1,7,7-trimethylbicyclo-[2.2.1]heptan-2-ylamino)methyl)benzyloxy)phenyl)propanoic acid and 3-(3-(3-(((1R,2R,4R)-1,7,7-trimethylbicyclo [2.2.1]heptan-2-ylamino)methyl)benzyloxy)phenyl)propanoic acid, were shown to have a pronounced hypoglycemic effect in the oral glucose tolerance test with CD-1 mice.

## 1. Introduction

Type 2 diabetes mellitus (T2DM) is a major public health problem worldwide. The global diabetes prevalence in 2019 was estimated to be 463 million people worldwide, with 90% of cases associated with type 2 diabetes [1]. More than one million deaths each year can be associated with T2DM, making it the ninth leading cause of death worldwide. [2]. The development of T2DM is considered to be caused by insulin resistance, which is triggered by a combination of two main factors: defective insulin secretion by pancreatic β-cells and the inability of insulin-sensitive tissues to respond to insulin [3,4]. Insulin resistance leads to a chronic condition of elevated blood glucose levels, i.e., hyperglycemia, which, if ignored, can lead to serious complications such as nephropathy [5], retinopathy [6], and cardiovascular system disorders [7].

Nowadays, T2DM pharmacotherapy is symptomatic and consists of normalizing glucose levels. There are several classes of hypoglycemic drugs that act on different biological targets and are able to exert a hypoglycemic effect. Therapy selection is individualized for each patient, which makes the search for new classes of hypoglycemic agents an important task [8]. New hypoglycemic agents are being scouted both for known targets, such as dipeptidyl peptidase-4 (DPP4) inhibitors [9,10], thiazolidinediones [11,12], α-glycosidase inhibitors [13,14,15], and sodium-glucose linked transporter-2 (SGLT-2) inhibitors [16,17], as well as for new targets with no approved drugs yet available. A fairly new target for the creation of hypoglycemic agents is free fatty acid receptor-1 (FFAR1), previously known as GPR40. FFAR1 agonists are considered to be promising hypoglycemic agents [18].

FFAR1 is a G protein-coupled receptor highly expressed by pancreatic β-cells that predominantly couples to the Gaq/11 G-protein subunit. In pancreatic β-cells, activation of FFAR1 promotes dissociation of Gαq protein, which further activates phospholipase C, cleaving phosphatidylinositol 4,5-bisphosphate into inositol triphosphate (IP3) and diacylglycerol (DAG) [18]. IP3 triggers calcium mobilization from the endoplasmic reticulum, causing insulin secretion [19], and DAG can activate protein kinase C and protein kinase D1, which may also stimulate insulin secretion [20]. It is worth noting that expression of the gene encoding FFAR1 increases with increasing blood glucose concentrations and decreases after blood glucose level normalization, which is considered to prevent hypoglycemia when using agonists of this receptor.

Some studies suggest the use of FFAR1 agonists for therapy of other diseases, such as alleviating organ inflammation and fibrosis, as well as for the treatment of central nervous system disorders such as Alzheimer’s disease and dementia [21].

The endogenous ligands of this receptor are medium- and long-chain fatty acids, such as docosahexaenoic acid (DHA) [22], but synthetic agonists tend to mimic endogenous ligands by using a phenylpropanoic acid fragment [23]. Previously, we synthesized a (hydroxyphenyl)propanoic acid derivative **QS-528** containing a terpene fragment, which was shown to have good affinity for the receptor discussed and also to induce a hypoglycemic effect in the oral glucose tolerance test (OGTT) with mice [24]. Further studies of this compound revealed other useful effects, as we propose, reduction of fatty degeneration of the liver and a hepatoprotective effect in mice [25,26]. Based on our results, we consider the structural analogs of **QS-528** to be promising compounds for subsequent studies.

**Figure 1:**
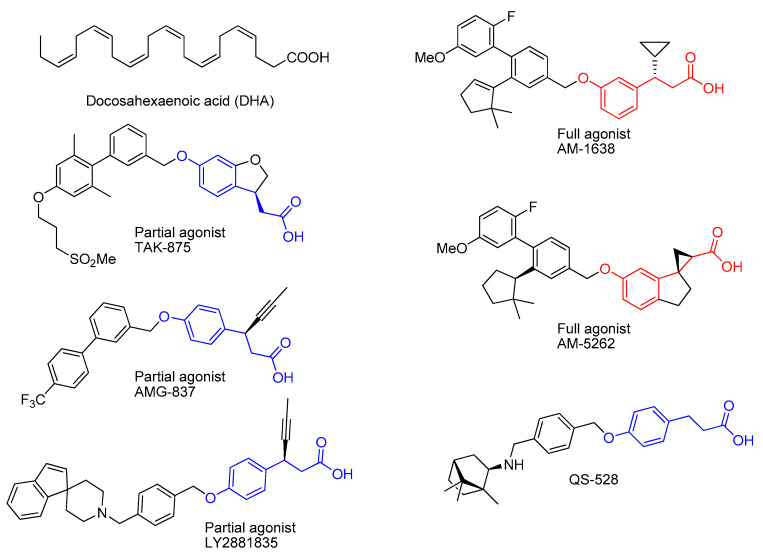
Endogenous (DHA) and synthetic FFAR1 (TAK-875, AMG-837, LY2881835, AM-1638, AM-5262, **QS-528**) agonists.

Some researchers [21,23,27] have noted that compounds containing a meta-substituted fragment of phenylpropanoic acid are more likely to exhibit full agonist properties (Figure 1). Full agonists exhibit superior levels of receptor activation in vitro and demonstrate superior efficacy in vivo versus the previously described partial agonists. In addition to stimulating insulin secretion via pancreatic action, full agonists have been reported to stimulate glucagon-like peptide-1 (GLP-1) secretion in the gut, potentially accounting for the observed enhancement in efficacy [28].

The aim of this work was to synthesize structural analogs of compound **QS-528** by varying the relative positions of structural fragments in the molecule, including the synthesis of structural analogs containing a meta-substituted fragment of phenylpropanoic acid, for further exploring the structure–activity relationship in in vitro and in vivo. In this work, we evaluated the influence of target compounds on cellular glucose uptake and their hypoglycemic effect in the oral glucose tolerance test in mice.

## 2. Results

### 2.1. Chemistry

**Scheme 1:**
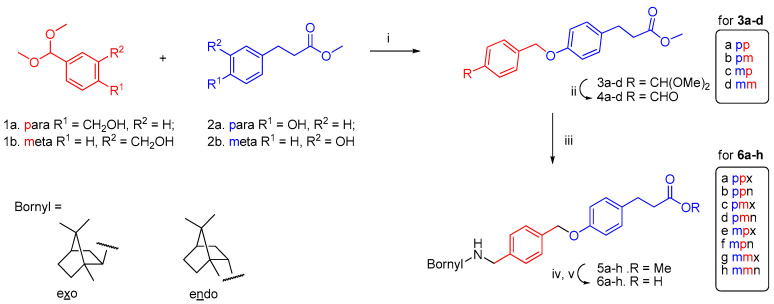
Target compounds synthesis. Only the para-para-substituted version of compounds **3**–**6** is presented in the scheme. Reagents and conditions: (i) DIAD, PPh_3_, THF, 0 °C to r.t.; (ii) 3% H_2_SO_4_, THF. Reagents and conditions: (iii) bornylamine (bornylNH_2_), NaBH(OAc)_3_, AcOH, CH_2_Cl_2_, 0 °C to r.t.; (iv) LiOH, MeOH, THF, H_2_O; (v) HCl, H_2_O.

The proposed scheme for the synthesis of the target compounds was as follows (Scheme 1). The Mitsunobu reaction was used to conjugate alcohols **1a**,**b** with phenols **2a**,**b** [29]. The reaction of alcohols **1a,b** with a small excess of phenols **2a**,**b**, diisopropyl azodicarboxylate (DIAD), and PPh_3_ was carried out in tetrahydrofuran (THF) for 48 h, and the products **3a**–**d** were isolated by column chromatography. The yields of the obtained compounds **3a**–**d** (scaffolds with a protected aldehyde group) were in the range of 67–76%. The dimethylacetal protection was removed just before the next step by treating substances **3a**–**d** in THF with dilute sulfuric acid for a few hours at room temperature, which resulted in the formation of aldehydes **4a**–**d** in quantitative yield.

Reductive amination of aldehydes **4a**–**d** and exo- and endobornylamines was carried out in methylene chloride in the presence of an equimolar amount of acetic acid and a small excess of sodium triacetoxyborhydride. Compounds **5a**–**h** were isolated with yields of 67–81% after column chromatography.

Alkaline hydrolysis of compounds **5a**–**h** with lithium hydroxide followed by treatment of the reaction mixture with 2M hydrochloric acid allowed us to isolate the target compounds **6a**–**h** as hydrochlorides in yields of 48–67%.

### 2.2. In Vitro Study

Compounds **6a**–**h** were tested for FFAR1 activation using the Cayman FFAR1 Assay Kit at a single concentration (10 μM). **GW9508** was used as a positive control at the same concentration; its efficiency was taken as 100%. All the investigated compounds showed the ability to activate the receptor at a concentration of 10 μM. The efficacy of the compounds ranged from 76% to 120%.

The effects of compounds **6a**–**h** on glucose consumption and lactate release as well as their cytotoxicity was investigated in HepG2 (human liver cancer) cells at concentrations of 2.5, 5, 10, and 25 μM. All compounds were found to have no significant effect on cell viability over the whole range of concentrations investigated.

All compounds tested at concentrations of 2.5 and 5 μM showed no effect on glucose consumption and lactate release. Increasing the concentration of compounds to 10 and 25 μM (Table 1) led to increases in glucose consumption and lactate release to 30–40% and 18–37%, respectively (except **6e** and **6f** at a concentration of 10 μM). Additionally, we used known FFAR1 agonist **GW9508** at concentrations of 10 and 25 μM, which induced significant increases in glucose consumption to 44–54% and lactate release to 34–46%.

### 2.3. In Vivo Study

**Figure 2:**
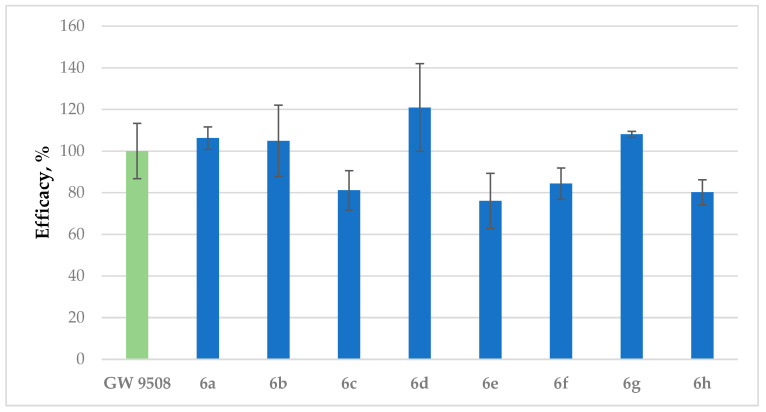
Effect of compounds **6a**–**h** (10 μM) on FFAR1 activation. The results are represented as mean ± SD, presented as a % of the value of **GW9508**.

**Figure 3:**
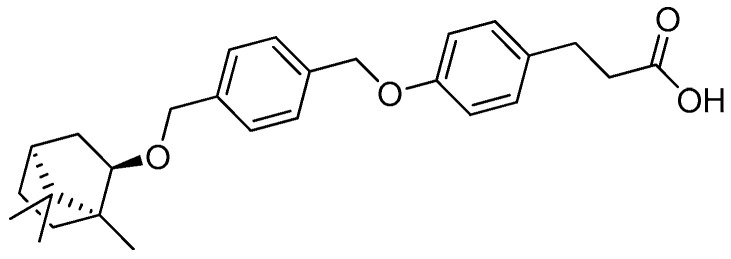
Structure of **QS-619**.

To evaluate the hypoglycemic effect of synthesized compounds **6a**–**h**, the OGTT was conducted with CD-1 mice at doses of 5 and 15 mg/kg. Vildagliptin (Galvus^®^) was used as a positive control at a dose of 10 mg/kg. Table 2 shows the differences between the AUC (area under the curve) values of the experimental and control groups, presented in percentage. The data obtained indicated that compound **6e** at the studied doses induced a hypoglycemic effect in a dose-dependent manner, since increasing the dose led to a stronger effect (Figure 2). Compound **6g** showed a hypoglycemic effect only at a dose of 15 mg/kg (Figure 3).

## 3. Discussion

The structures of FFAR1 agonists, as can be seen in Figure 1, are linear molecules in which the acid head (phenylpropanoic acid or its analog) is linked through an oxygen atom to the tail part of the molecule. Generally, synthesis of this type of structure involves parallel synthesis of the ester (hydroxyphenyl)propanoic acid and the tail part, and their conjugation reaction is followed by hydrolysis of the ester group, which leads to the formation of the target compounds. Previously, we synthesized compound **QS-528** containing a fragment of (4-hydroxyphenyl)propanoic acid and a bornyl fragment [24]. **QS-528** was shown to be an effective FFAR1 receptor agonist and induced a hypoglycemic effect in mice at doses of 1–25 mg/kg. The key difference between **QS-528** and the other synthetic agonists shown in Figure 1 is the absence of substituents in the aliphatic part of the phenylpropanoic acid. The purpose of the substituent in the aliphatic part of phenylpropanoic acid is to reduce β-oxidation and increase selectivity toward the target receptor [30].

Indeed, phenylpropanoic acid derivatives are likely to interact with a wide variety of targets present in the body, some of which are also considered targets for anti-diabetic agents. Among these targets, in addition to FFAR1 mentioned earlier, are free fatty acid receptor-4 (FFAR4) [31], peroxisome proliferator-activated receptor alpha (PPARα) [32,33], peroxisome proliferator-activated receptor gamma PPARγ [32,34], aldo-keto reductase AKR1B1 [34], glucose transporter type 4 (GLUT4) [34], and protein tyrosine phosphatase 1B (PTP1B) [35]. Currently, more and more papers are being published that have studied multi-target agents for the treatment of diabetes in particular, many of which are derivatives of phenylpropanoic acids or their analogs. As an example of such agents, the following can be mentioned: FFAR1-FFAR4 [36], FFAR1-PPARγ [37], PPARα-PPARγ [38,39].

Based on the above, we considered that such phenylpropanoic acid derivatives as **QS-528** may be promising antidiabetic agents with potential multitarget action. This assumption was supported by our consequent studies.

Compound **QS-528** was shown to induce a hypoglycemic effect in C57BL/6 mice but not in C57BL/6^Ay^ mice with developed diabetes, although some benefits were exhibited when administered for a prolonged time. **QS-528** exhibited hepatoprotective properties by restoring liver functions. In comparison, compound **QS-619** (Figure 2), which is an *O*-endobornyl analog of compound **QS-528**, induced a hypoglycemic effect in both C57BL/6 and C57BL/6^Ay^ mice but had no hepatoprotective effect [25].

In order to further evaluate the effect of the molecular shape on its anti-diabetic properties, we decided to synthesize all eight possible combinations for this structure by varying the bond from the para to the meta position in both aromatic rings as well as changing the configuration of the asymmetric center in the bornyl moiety.

Ortho-substituted compounds were discarded due to the lack of data about known successful examples as well as the available information about the lack of effectiveness of such compounds [30], which may be related to the strong conformation deformation as compared to the endogenous ligands.

Based on the literature analysis, we assumed that the meta-substituted derivatives were likely to exhibit better activity by being full agonists [40] or positive allosteric modulators [27].

**Scheme 2:**
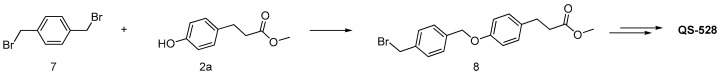
The previously described synthesis of the compound.

In previous work [24], we used 1,4-bis(bromomethyl)benzene **7** as a building block for the synthesis of the target compound **QS-528**, but in scaling up we faced problems in needing to use a considerable amount of dibromide as well as difficulties in its separation from the reaction product (Scheme 2).

**Scheme 3:**
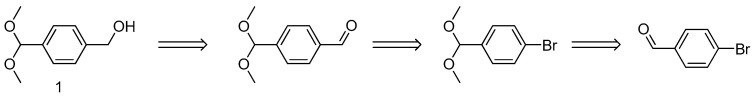
Synthetic approach to the key building block.

To avoid these problems, we suggested using another building block, ((dimethoxymethyl)phenyl)methanol **1**, which could be obtained from commercially available bromoaldehydes (Scheme 3).

The above-mentioned meta- or para-substituted building blocks **1a**–**b** were reacted with methyl esters of (hydroxyphenyl)propanoic acids **2a**–**b** under standard Mitsunobu conditions, and the products **3a**–**d** were isolated by column chromatography.

It is worth noting that in order to prevent the deprotection of the aldehyde group when working with all compounds containing the dimethyl acetal fragment discussed in this paper, their isolation from reaction mixtures and following purification via chromatography were performed with solvents containing 1% *v/v* of triethylamine.

The removal of dimethyl acetal protection from compounds **3a**–**d** occurred in quantitative yield when the substance solution in THF was treated with diluted aqueous sulfuric acid solution. When trying to purify aldehydes **4a**–**d** obtained by column chromatography, we noticed a partial decrease in the yield, so the optimal solution was to use the aldehydes in the next stage without additional purification.

The reductive amination using sodium triacetoxyborhydride proceeded smoothly for all combinations of aldehydes and amines. The compounds were purified by column chromatography on silica gel to afford compounds **5a**–**h**.

The use of lithium hydroxide allowed us to perform hydrolysis of the ester groups in compounds **5a**–**h** under mild conditions. Follow-up treatment of the reaction mixture with dilute hydrochloric acid led to precipitation of the target compounds as hydrochloride salts **6a**–**h**.

It can be noted that the proposed approach to the synthesis of the target substances was versatile; the yields of compounds did not strongly depend on the structure of the initial substrates. Despite the increased number of stages compared to our previous approach [24], we found the new approach to be more suitable for the production of larger amounts of compounds.

Thus, seven new structural analogs of **QS-528** were synthesized and studied using in vitro tests and the OGTT.

Glucose consumption and lactate release tests in HepG2 cells allowed us to estimate the effect of the compounds on glucose metabolism in liver cells, which can be used to further examine the mechanism of action [41,42]. The effects of compounds **6a-h** at concentrations of 2.5, 5, 10, and 25 μM on glucose uptake and lactate release in human liver cancer HepG2 cells were studied (Table 1). The cytotoxicity of these compounds on this cell line was also estimated.

At low concentrations (2.5 and 5 μM), the studied compounds had no effect on glucose uptake and lactate release, but as the concentration increased to 10 and 25 μM, there was a dose-dependent increase in both glucose uptake and lactate release. It can be noted that the exobornyl derivatives (**6a**,**c**,**e**,**g**) exhibited slightly higher activity compared to their endo (**6b**,**d**,**f**,**h**) counterparts (except for the pair of compounds **6g** and **6h** at 10 μM). The highest difference compared to the control group was found for compound **6a** (**QS-528**).

An interesting effect was observed for compounds **6c,h** at a concentration of 10 μM, with a noticeable increase in glucose uptake but little difference in lactate release compared to the control group, which may be explained by the accumulation of glucose in cells.

There was no significant difference between para and meta substitution in any of the two aromatic fragments. With the exception of compound **6a** (**QS-528)**, compounds **6b-h** appeared to have similar effects, with no significant difference in their effects on cellular glucose uptake and lactate release.

Analyzing the FFAR1 activation data (Figure 2), we observed that all the compounds studied were agonists of the FFA1 receptor at a concentration of 10 μM. The efficacy of compounds **6a**–**h** ranged from 76% to 120%. Compounds **6a**,**b**,**d**,**g** showed greater efficacy (104–121%) whereas compounds **6c**,**e**,**f**,**h** showed slightly less efficacy (76–84%). It seemed that the derivatives containing a para-substituted phenylpropanoic acid fragment tended to exhibit greater efficacy than their meta-substituted counterparts (except for compounds **6c** and **6g**).

Then we examined the hypoglycemic effect of the compounds in CD-1 mice (Table 2). The doses were chosen based on previous work [24]. In mice, derivative **6e** containing a meta-substituted fragment of phenylpropanoic acid, a para-substituted linker, and an exobornyl fragment showed the clearest effect in the OGTT, showing a dose-dependent hypoglycemic effect. Its analogue **6g** containing a meta-substituted fragment of phenylpropanoic acid, a meta-substituted linker, and an exobornyl fragment showed an effect only at the highest dose of 15 mg/kg studied. The other compounds did not demonstrate a hypoglycemic effect in the OGTT. The missing hypoglycemic effect induced by compound **6a** (**QS-528**) in the OGTT may have been due to the use of a different mouse line compared with previously published results [24]. Despite the absence of a clear correlation between the data on glucose uptake, lactate release, FFAR1 activation, and OGTT, we can make some assumptions about the relationship between the structure and activity of the compounds studied. Based on our results, we can suggest that the exobornyl fragment is a more privileged structure than the endobornyl fragment. The lack of hypoglycemic effect of compounds **6f**,**h**, which are endobornyl analogs of compounds **6e**,**g**, can be explained by the lower metabolic stability of these compounds due to the more reactive amino group, thus causing their higher steric accessibility.

Apparently, the discrepancy in the results of the OGTT, FFAR1 activation, and glucose consumption can be attributed to the differing pharmacokinetics of the studied compounds. Thus, the synthesized compounds demonstrated interesting properties in terms of the search for new antidiabetic agents, which indicates the need for further in-depth research.

## 4. Materials and Methods

### 4.1. Chemistry

#### 4.1.1. General

Dichloromethane was refluxed over phosphorus pentoxide and distilled. Tetrahydrofuran (THF) was refluxed over sodium/benzophenone and distilled in an inert atmosphere. 3-Hydroxybenzaldehyde and 4-hydroxybenzaldhyde were obtained from Acros Organics (Geel, Belgium). 3-Bromobenzaldhyde, 4-bromobenzaldehyde and diisopropyl azodicarboxylate was purchased from Fluorochem (Hadfield, UK). (*+*)-Camphor was purchased from TCI Co. (Tokyo, Japan). Triphenylphosphine was purchased from Alfa Aesar (Heysham, UK). Lithium hydroxide monohydrate was purchased from Fisher Scientific (Loughborough, UK). ^1^H and ^13^C NMR spectra were acquired on Bruker spectrometers AV-400 at 400.13 MHz (^1^H) and DRX-500 at 500.13 MHz (^1^H) and 125.76 MHz (^13^C) in deuterated chloroform (CDCl_3_) or deuterated dimethyl sulfoxide (DMSO-d_6_); chemical shifts in parts per million (ppm) were measured relative to residual CHCl_3_ [(CHCl_3_) 7.26, (CHCl_3_) 77.00 ppm] or DMSO-d_6_ [(DMSO-d_6_) 2.50, (DMSO-d_6_) 39.51 ppm], and *J* was measured in Hertz. The attached proton test (APT) modification of the *J*-modulated spin-echo (JMOD) experiment was performed. The structures of the products were determined by means of ^1^H and ^13^C NMR spectra (^1^H and ^13^C NMR spectra of compounds **3**–**6** (Figures S1–S63) are included in Supplementary Materials). High-resolution mass spectrometry (HRMS) was conducted using a DFS Thermo Scientific spectrometer in full scan mode (m/z 15–500, 70 eV electron impact ionization, and direct sample injection). Column chromatography was performed on silica gel (60–200 mesh, Macherey-Nagel, Düren, Germany).

#### 4.1.2. General Procedure for Mitsunobu Reaction

Diisopropyl azodicarboxylate (DIAD) (1.0 mL, 5.1 mmol) was added dropwise to an ice-cooled stirred solution of alcohol **1** (880 mg, 4.8 mmol), phenol **2** (914 mg, 5.1 mmol), and PPh_3_ (1.330 g, 5.1 mmol) in freshly distilled tetrahydrofuran (10 mL). After the addition of DIAD, the resulting solution was stirred for 48 h at room temperature. The solvent was distilled off under reduced pressure and the residue was purified by column chromatography using hexane–ethyl acetate–triethylamine (100:10:1) as the eluent.

Methyl 3-(4-(4-(dimethoxymethyl)benzyloxy)phenyl)propanoate (**3a**). White solid, yield 67%. ^1^H NMR (500 MHz, CDCl_3_): δ = 7.50–7.40 (m, 4H), 7.11 (d, *J* = 8.5 Hz, 2H), 6.90 (d, *J* = 8.5 Hz, 2H), 5.40 (s, 1H), 5.04 (s, 2H), 3.67 (s, 3H), 3.33 (s, 6H), 2.89 (t, *J* = 7.8 Hz, 2H), 2.60 (t, *J* = 7.8 Hz, 2H). ^13^C NMR (126 MHz, CDCl_3_): δ = 173.4, 157.2, 137.7, 137.3, 132.9, 129.2, 127.2, 126.9, 114.8, 102.8, 69.7, 52.7, 51.6, 35.9, 30.0. HRMS for C_20_H_24_O_5_^+^ calcd 344.1618, found 344.1613 [M]^+^.

Methyl 3-(4-(3-(dimethoxymethyl)benzyloxy)phenyl)propanoate (**3b**). Colorless oil, yield 76%. ^1^H NMR (300 MHz, CDCl_3_): δ = 7.52 (s, 1H), 7.45–7.35 (m, 3H), 7.11 (d, *J* = 8.5 Hz, 2H), 6.90 (d, *J* = 8.5 Hz, 2H), 5.41 (s, 1H), 5.05 (s, 2H), 3.67 (s, 3H), 3.34 (s, 6H), 2.94–2.85 (m, 2H), 2.65–2.56 (m, 2H). ^13^C NMR (101MHz, CDCl_3_): δ = 173.4, 157.2, 138.4, 137.1, 132.9, 129.2, 128.5, 127.5, 126.3, 125.7, 114.9, 102.9, 69.9, 52.7, 51.5, 35.9, 30.1. HRMS for C_20_H_24_O_5_^+^ calcd 344.1618, found 344.1613 [M]^+^.

Methyl 3-(3-(4-(dimethoxymethyl)benzyloxy)phenyl)propanoate (**3c**). Colorless oil, yield 75%. ^1^H NMR (300 MHz, CDCl_3_): δ = 7.51–7.40 (m, 4H), 7.24–7.16 (m, 1H), 6.85–6.78 (m, 3H), 5.41 (s, 1H), 5.05 (s, 2H), 3.67 (s, 3H), 3.34 (s, 6H), 2.93 (t, *J* = 7.6 Hz, 2H), 2.64 (t, *J* = 7.6 Hz, 2H). ^13^C NMR (101MHz, CDCl_3_): δ = 173.3, 158.9, 142.1, 137.8, 137.2, 129.5, 127.3 (2C), 126.9 (2C), 120.9, 115.0, 112.4, 102.9, 69.6, 52.6 (2C), 51.6, 35.5, 30.9. HRMS for C_20_H_24_O_5_^+^ calcd 344.1618, found 344.1619 [M]^+^.

Methyl 3-(3-(3-(dimethoxymethyl)benzyloxy)phenyl)propanoate (**3d**). Colorless oil, yield 72%. ^1^H NMR (400 MHz, CDCl_3_): δ = 7.53 (s, 1H), 7.44–7.37 (m, 3H), 7.23–7.18 (m, 1H), 6.85–6.79 (m, 3H), 5.42 (s, 1H), 5.06 (s, 2H), 3.67 (s, 3H), 3.34 (s, 6H), 2.93 (t, *J* = 7.9 Hz, 2H), 2.63 (t, *J* = 7.9 Hz, 2H). ^13^C NMR (101 MHz, CDCl_3_): δ = 173.3, 158.9, 142.1, 138.4, 137.0, 129.5, 128.5, 127.5, 126.3, 125.8, 120.9, 115.0, 112.5, 102.9, 69.8, 52.7 (2C), 51.6, 35.5, 30.9. HRMS for C_20_H_24_O_5_^+^ calcd 344.1618, found 344.1621 [M]^+^.

#### 4.1.3. General Procedure for Dimethylacetal Deprotection

To a solution of dimethyl acetal **3a**–**d** (0.98 g, 2.9 mmol) in tetrahydrofuran (10 mL), a solution of 3% sulfuric acid in water (4 mL) was added and the resulting solution was stirred at room temperature for 3 h. Ethyl acetate (20 mL) and water (20 mL) were added and the organic layer was separated. The aqueous layer was extracted with ethyl acetate (2 × 20 mL). The combined organic extracts were washed with brine (10 mL) and dried over anhydrous sodium sulfate overnight. The drying agent was filtered off and washed with a small amount of ethyl acetate. The combined solutions were evaporated under reduced pressure and the residue was used in the next step without further purification.

Methyl 3-(4-(4-formylbenzyloxy)phenyl)propanoate (**4a**). White solid, yield 95%. ^1^H NMR (400 MHz, CDCl_3_): δ = 10.02 (s, 1H), 7.90 (d, *J* = 8.1 Hz, 2H), 7.60 (d, *J* = 8.1 Hz, 2H), 7.13 (d, *J* = 8.6 Hz, 2H), 6.89 (d, *J* = 8.6 Hz, 2H), 5.13 (s, 2H), 3.66 (s, 3H), 2.90 (t, *J* = 7.7 Hz, 3H), 2.60 (t, *J* = 7.8 Hz, 3H).

Methyl 3-(4-(3-formylbenzyloxy)phenyl)propanoate (**4b**). Colorless oil, yield 92%. ^1^H NMR (300 MHz, CDCl_3_): δ = 10.03 (s, 1H), 7.95 (s, 1H), 7.83 (d, *J* = 7.6 Hz, 1H), 7.70 (d, *J* = 7.6 Hz, 1H), 7.59–7.51 (m, 1H), 7.12 (d, *J* = 8.5 Hz, 2H), 6.89 (d, *J* = 8.5 Hz, 2H), 5.11 (s, 2H), 3.66 (s, 3H), 2.94–2.85 (m, 2H), 2.65–2.56 (m, 2H).

Methyl 3-(3-(4-formylbenzyloxy)phenyl)propanoate (**4c**). Colorless oil that solidified upon standing, yield 94%. ^1^H NMR (500 MHz, CDCl_3_): δ = 10.02 (s, 1H), 7.90 (d, *J* = 7.9 Hz, 2H), 7.60 (d, *J* = 7.9 Hz, 2H), 7.24–7.20 (m, 1H), 6.85–6.79 (m, 3H), 5.14 (s, 2H), 3.67 (s, 3H), 2.93 (t, *J* = 7.8 Hz, 2H), 2.63 (t, *J* = 7.8 Hz, 2H).

Methyl 3-(3-(3-formylbenzyloxy)phenyl)propanoate (**4d**). Colorless oil, yield 92%. ^1^H NMR (400 MHz, CDCl_3_): δ = 10.04 (s, 1H), 7.96 (s, 1H), 7.84 (d, *J* = 7.7 Hz, 1H), 7.71 (d, *J* = 7.7 Hz, 1H), 7.59–7.53 (m, 1H), 7.25–7.19 (m, 1H), 6.86–6.80 (m, 3H), 5.12 (s, 2H), 3.67 (s, 3H), 2.93 (t, *J* = 7.9 Hz, 2H), 2.63 (t, *J* = 7.9 Hz, 2H).

#### 4.1.4. General Procedure for Reductive Amination

To an ice-cooled solution of aldehyde **4a**–**d** (1 equiv), exo- or endobornylamine (1.15 equiv), and acetic acid (1 equiv) in dichloromethane (5 mL per 1 mmol of aldehyde), sodium triacetoxyborohydride (1.5 equiv) was added portionwise in 5 min. Then, after 30 min, the cooling was removed and the resulting solution was stirred overnight at room temperature. Dichloromethane (20 mL) and 5% potassium carbonate (10 mL) solution were added. The organic layer was separated and the aqueous layer was extracted with dichloromethane (2 × 10 mL). The combined organic layers were washed with 5% potassium carbonate (10 mL) solution, water (10 mL), and brine (10 mL) and dried over anhydrous sodium sulfate. The drying agent was filtered off and washed with a small amount of ethyl acetate. The combined solutions were evaporated under reduced pressure and the residue was purified over silica gel column chromatography using chloroform–ethanol (100:1).

Methyl 3-(4-(4-(((1*R*,2*R*,4*R*)-1,7,7-trimethylbicyclo [2.2.1]heptan-2-ylamino)methyl)benzyloxy)phenyl)propanoate (**5a**). White solid, yield 71%. ^1^H NMR (400 MHz, CDCl_3_): δ = 7.40–7.31 (m, 4H), 7.11 (d, *J* = 8.6 Hz, 2H), 6.90 (d, *J* = 8.6 Hz, 2H), 5.01 (s, 2H), 3.78 (d, *J* = 13.4 Hz, 1H), 3.67 (s, 3H), 3.61 (d, *J* = 13.4 Hz, 1H), 2.89 (t, *J* = 7.8 Hz, 2H), 2.63–2.56 (m, 3H), 1.73–1.47 (m, 5H), 1.23–1.13 (m, 1H), 1.10–1.03 (m, 5H), 0.89 (s, 3H), 0.82 (s, 3H). ^13^C NMR (126 MHz, CDCl_3_): δ = 173.4, 157.3, 141.1, 135.4, 132.8, 129.2 (2C), 128.3 (2C), 127.5 (2C), 114.8 (2C), 69.9, 66.1, 52.2, 51.6, 48.4, 46.7, 45.3, 38.7, 36.8, 36.0, 30.1, 27.3, 20.6, 20.5, 12.2. HRMS for C_28_H_37_O_3_N^+^ calcd 435.2768, found 435.2760 [M]^+^.

Methyl 3-(4-(4-(((1*R*,2*S*,4*R*)-1,7,7-trimethylbicyclo [2.2.1]heptan-2-ylamino)methyl)benzyloxy)phenyl)propanoate (**5b**). White solid, yield 67%. ^1^H NMR (300 MHz, CDCl_3_): δ = 7.41–7.33 (m, 4H), 7.11 (d, *J* = 8.6 Hz, 2 H), 6.90 (d, *J* = 8.6 Hz, 2H), 5.02 (s, 2H), 3.86 (d, *J* = 13.3 Hz, 1H), 3.73 (d, *J* = 13.3 Hz, 1H), 3.67 (s, 3H), 2.95–2.84 (m, 3H), 2.60 (t, *J* = 8.2 Hz, 2H), 2.22–2.09 (m, 1H), 1.91–1.60 (m, 3H), 1.34–1.13 (m, 3H), 0.90–0.81 (m, 10H). ^13^C NMR (101MHz, CDCl_3_): δ = 173.4, 157.3, 141.1, 135.4, 132.8, 129.2 (2C), 128.2 (2C), 127.5 (2C), 114.8 (2C), 69.9, 62.6, 52.8, 51.6, 48.8, 48.3, 45.0, 38.0, 35.9, 30.1, 28.4, 27.4, 19.8, 18.7, 14.3. HRMS for C_28_H_37_O_3_N^+^ calcd 435.2768, found 435.2765 [M]^+^.

Methyl 3-(4-(3-(((1*R*,2*R*,4*R*)-1,7,7-trimethylbicyclo [2.2.1]heptan-2-ylamino)methyl)benzyloxy)phenyl)propanoate (**5c**). Slightly yellow oil, yield 71%. ^1^H NMR (300 MHz, CDCl_3_): δ = 7.40–7.27 (m, 4H), 7.11 (d, *J* = 8.7 Hz, 2H), 6.91 (d, *J* = 8.7 Hz, 2H), 5.03 (s, 2H), 3.79 (d, *J* = 13.4 Hz, 1H), 3.67 (s, 3H), 3.63 (d, *J* = 13.4 Hz, 1H), 2.90 (t, *J* = 7.5 Hz, 2H), 2.64–2.55 (m, 3H), 1.76–1.46 (m, 5H), 1.45–1.31 (m, 1H), 1.10–1.02 (m, 5H), 0.89 (s, 3H), 0.82 (s, 3H). ^13^C NMR (126 MHz, CDCl_3_): δ = 173.4, 157.3, 141.8, 136.9, 132.8, 129.2 (2C), 128.5, 127.7, 127.2, 125.8, 114.8 (2C), 70.1, 66.2, 52.4, 51.6, 48.4, 46.7, 45.3, 38.8, 36.8, 36.0, 30.1, 27.3, 20.6, 20.6, 12.2. HRMS for C_28_H_37_O_3_N^+^ calcd 435.2768, found 435.2764 [M]^+^.

Methyl 3-(4-(3-(((1*R*,2*S*,4*R*)-1,7,7-trimethylbicyclo [2.2.1]heptan-2-ylamino)methyl)benzyloxy)phenyl)propanoate (**5d**). Slightly yellow oil, yield 70%. ^1^H NMR (300 MHz, CDCl_3_): δ = 7.43–7.27 (m, 4H), 7.11 (d, *J* = 8.5 Hz, 2H), 6.91 (d, *J* = 8.5 Hz, 2H), 5.03 (s, 2H), 3.87 (d, *J* = 13.4 Hz, 1H), 3.74 (d, *J* = 13.4 Hz, 1H), 3.67 (s, 3H), 2.93–2.83 (m, 3H), 2.60 (t, *J* = 8.2 Hz, 2H), 2.21–2.09 (m, 1H), 1.90–1.80 (m, 1H), 1.70 (dt, *J* = 3.9, 7.7 Hz, 1H), 1.64–1.59 (m, 1H), 1.45–1.35 (m, 1H), 1.34–1.13 (m, 2H), 0.88–0.81 (m, 10H). ^13^C NMR (126 MHz, CDCl_3_): δ = 173.4, 157.3, 141.7, 137.0, 132.8, 129.2 (2C), 128.5, 127.6, 127.1, 125.9, 114.8 (2C), 70.1, 62.6, 52.9, 51.6, 48.8, 48.3, 45.0, 38.0, 36.0, 30.1, 28.4, 27.4, 19.8, 18.7, 14.3. HRMS for C_28_H_37_O_3_N^+^ calcd 435.2768, found 435.2764 [M]^+^.

Methyl 3-(3-(4-(((1*R*,2*R*,4*R*)-1,7,7-trimethylbicyclo [2.2.1]heptan-2-ylamino)methyl)benzyloxy)phenyl)propanoate (**5e**). Colorless oil, yield 80%. ^1^H NMR (400 MHz, CDCl_3_): δ = 7.41–7.33 (m, 4H), 7.24–7.19 (m, 1H), 6.86–6.79 (m, 3H), 5.03 (s, 2H), 3.79 (d, *J* = 13.4 Hz, 1H), 3.68 (s, 3H), 3.62 (d, *J* = 13.4 Hz, 1H), 2.94 (t, *J* = 7.9 Hz, 2H), 2.67–2.58 (m, 3H), 1.74–1.47 (m, 5H), 1.21 (br. s., 1H), 1.13–1.04 (m, 5H), 0.90 (s, 3H), 0.83 (s, 3H). ^13^C NMR (101 MHz, CDCl_3_) δ = 173.3, 158.9, 142.1, 141.3, 135.2, 129.5, 128.3 (2C), 127.5 (2C), 120.8, 114.9, 112.4, 69.8, 66.1, 52.2, 51.6, 48.4, 46.7, 45.3, 38.7, 36.8, 35.5, 30.9, 27.4, 20.6, 20.5, 12.2. HRMS for C_28_H_37_O_3_N^+^ calcd 435.2768, found 435.2770 [M]^+^.

Methyl 3-(3-(4-(((1*R*,2*S*,4*R*)-1,7,7-trimethylbicyclo [2.2.1]heptan-2-ylamino)methyl)benzyloxy)phenyl)propanoate (**5f**). Slightly yellow oil, yield 67%. ^1^H NMR (400 MHz, CDCl_3_): δ = 7.41–7.34 (m, 4H), 7.24–7.18 (m, H), 6.86–6.78 (m, 3H), 5.03 (s, 2H), 3.87 (d, *J* = 13.4 Hz, 1H), 3.75 (d, *J* = 13.4 Hz, 1H), 3.68 (s, 3H), 2.93 (t, *J* = 7.7 Hz, 2H), 2.87 (ddd, *J* = 1.6, 3.9, 9.9 Hz, 1H), 2.63 (t, *J* = 7.9 Hz, 2H), 2.20–2.11 (m, 1H), 1.89–1.81 (m, 1H), 1.76–1.66 (m, 1 H), 1.62 (s, 1H), 1.33–1.14 (m, 3H), 0.89–0.82 (m, 10H). ^13^C NMR (101MHz, CDCl_3_): δ = 173.3, 158.9, 142.1, 141.1, 135.3, 129.5, 128.2 (2C), 127.5 (2C), 120.8, 115.0, 112.4, 69.8, 62.6, 52.8, 51.6, 48.8, 48.3, 45.0, 38.0, 35.5, 30.9, 28.4, 27.4, 19.8, 18.7, 14.3. HRMS for C_28_H_37_O_3_N^+^ calcd 435.2768, found 435.2765 [M]^+^.

Methyl 3-(3-(3-(((1*R*,2*R*,4*R*)-1,7,7-trimethylbicyclo [2.2.1]heptan-2-ylamino)methyl)benzyloxy)phenyl)propanoate (**5g**). Slightly yellow oil, yield 81%. ^1^H NMR (300 MHz, CDCl_3_): δ = 7.41–7.27 (m, 4 H), 7.25–7.18 (m, 1 H), 6.87–6.78 (m, 3 H), 5.04 (s, 2 H), 3.84–3.77 (m, 1 H), 3.68 (s, 3 H), 3.67–3.60 (m, 1 H), 2.97−2.90 (t, 7.5 Hz 2 H), 2.67–2.56 (m, 3 H), 1.75–1.46 (m, 5 H), 1.33 (br. s., 1 H), 1.11–1.02 (m, 5 H), 0.89 (s, 3 H), 0.82 (s, 3 H). ^13^C NMR (126MHz, CDCl_3_): δ = 173.3, 158.9, 142.1, 141.8, 136.8, 129.5, 128.5, 127.7, 127.2, 125.9, 120.8, 114.9, 112.4, 69.9, 66.2, 52.4, 51.6, 48.4, 46.7, 45.3, 38.8, 36.8, 35.5, 30.9, 27.4, 20.6, 20.6, 12.2. HRMS for C_28_H_37_O_3_N^+^ calcd 435.2768, found 435.2773 [M]^+^.

Methyl 3-(3-(3-(((1*R*,2*S*,4*R*)-1,7,7-trimethylbicyclo [2.2.1]heptan-2-ylamino)methyl)benzyloxy)phenyl)propanoate (**5h**). Slightly yellow oil, yield 70%. ^1^H NMR (400 MHz, CDCl_3_): δ = 7.41 (s, 1 H), 7.38–7.28 (m, 3 H), 7.21 (t, *J* = 7.9 Hz, 1 H), 6.87–6.78 (m, 3 H), 5.04 (s, 2 H), 3.91–3.84 (d, *J* = 13.4 Hz, 1 H), 3.78–3.72 (d, *J* = 13.4 Hz, 1 H), 3.70–3.65 (s, 3 H), 2.96–2.90 (m, *J* = 7.9 Hz 2 H), 2.89−2.84 (ddd, 9.9, 4.0, 1.9 1 H), 2.63 (t, *J* = 7.9 Hz, 2 H), 2.20–2.10 (m, 1 H), 1.84 (m, 1 H), 1.70 (m, 1 H), 1.62 (m, 1 H), 1.49–1.35 (br. s, 1 H), 1.33–1.14 (m, 2 H), 0.88–0.82 (m, 10 H). ^13^C NMR (101MHz, CDCl_3_): δ = 173.3, 159.0, 142.1, 141.7, 136.9, 129.5, 128.5, 127.6, 127.1, 125.9, 120.8, 115.0, 112.4, 70.0, 62.7, 53.0, 51.6, 48.8, 48.4, 45.1, 38.1, 35.6, 30.9, 28.5, 27.4, 19.8, 18.7, 14.3. HRMS for C_28_H_37_O_3_N^+^ calcd 435.2768, found 435.2766 [M]^+^.

#### 4.1.5. General Procedure for Hydrolysis

To an ice-cooled stirred solution of methyl ester (0.195 mmol) in THF (3 mL) and methanol (1 mL), water (2 mL) was added followed by the addition of lithium hydroxide monohydrate (0.390 mmol). After completion of the reaction (monitored by TLC), the THF was evaporated under reduced pressure. Water (10 mL) was added and then 2M hydrochloric acid was added dropwise until pH 2–3 was reached. The resulting solution was left overnight in a refrigerator. The precipitate was filtered, washed with water, and dried in a vacuum desiccator over phosphorus pentoxide.

(1*R*,2*R*,4*R*)-*N*-(4-((4-(2-carboxyethyl)phenoxy)methyl)benzyl)-1,7,7-trimethylbicyclo [2.2.1]heptan-2-aminium chloride (**6a**). White solid, yield 71%. ^1^H NMR (300 MHz, CDCl_3_): δ = 7.63 (d, *J* = 8.1 Hz, 2H), 7.40 (d, *J* = 8.1 Hz, 2H), 7.12 (d, *J* = 8.5 Hz, 2H), 6.84 (d, *J* = 8.5 Hz, 2H), 4.93 (s, 2H), 4.22 (d, *J* = 13.7 Hz, 1H), 4.07 (d, *J* = 13.7 Hz, 1H), 2.88 (t, *J* = 7.3 Hz, 2H), 2.81 (dd, *J* = 5.0, 8.8 Hz, 1H), 2.63 (t, *J* = 7.3 Hz, 2H), 2.32 (dd, *J* = 2.9, 13.8 Hz, 1H), 1.81 (t, *J* = 3.7 Hz, 1H), 1.74–1.46 (m, 3H), 1.15 (s, 3H), 1.09 (s, 3H), 1.05–0.88 (m, 2 H), 0.82 (s, 3 H). ^13^C NMR (126 MHz, DMSO-d_6_): δ = 173.9, 156.6, 138.2, 133.2, 131.1, 131.0 (2C), 129.3 (2C), 127.8 (2C), 114.8 (2C), 68.8, 64.2, 50.1, 48.6, 47.0, 44.3, 36.3, 35.6, 34.7, 29.6, 26.3, 20.4, 19.9, 11.8. HRMS for C_27_H_35_O_3_N^+^ calcd 421.2612, found 421.2610 [M]^+^.

(1*R*,2*S*,4*R*)-*N*-(4-((4-(2-carboxyethyl)phenoxy)methyl)benzyl)-1,7,7-trimethylbicyclo [2.2.1]heptan-2-aminium chloride (**6b**). White solid, yield 62%. ^1^H NMR (400 MHz, DMSO-d_6_): δ = 12.14 (s, 1H), 9.53 (br. s., 1H), 8.93 (br. s., 1H), 7.65 (d, *J* = 8.1 Hz, 2H), 7.47 (d, *J* = 8.1 Hz, 2H), 7.12 (d, *J* = 8.6 Hz, 2H), 6.89 (d, *J* = 8.6 Hz, 2H), 5.09 (s, 2H), 4.25–4.16 (m, 1H), 4.12–4.03 (m, 1H), 3.10–3.01 (m, 1H), 2.73 (d, *J* = 7.5 Hz, 2H), 2.48–2.43 (m, 2H), 1.97–1.85 (m, 1H), 1.77–1.59 (m, 3H), 1.49–1.32 (m, 2H), 1.22 (dd, *J* = 4.2, 13.4 Hz, 1H), 0.88 (s, 3H), 0.80 (s, 3H), 0.69 (s, 3H). ^13^C NMR (126 MHz, DMSO-d_6_): δ = 173.9, 156.5, 138.2, 133.1, 131.1, 130.7 (2C), 129.2 (2C), 127.8 (2C), 114.7 (2C), 68.7, 62.2, 49.6, 48.9, 48.1, 43.6, 35.6, 32.1, 29.5, 27.3, 26.9, 19.1, 18.2, 13.2. HRMS for C_27_H_35_O_3_N^+^ calcd 421.2612, found 421.2608 [M]^+^.

(1*R*,2*R*,4*R*)-*N*-(3-((4-(2-carboxyethyl)phenoxy)methyl)benzyl)-1,7,7-trimethylbicyclo [2.2.1]heptan-2-aminium chloride (**6c**). White solid, yield 64%. ^1^H NMR (300 MHz, CDCl_3_): δ = 7.61 (s, 1H), 7.49–7.34 (m, 3H), 7.06 (d, *J* = 8.5 Hz, 2H), 6.83 (d, *J* = 8.5 Hz, 2H), 6.25 (br. s., 3H), 4.97 (s, 2H), 4.16–4.08 (m, 1H), 4.01–3.92 (m, 1H), 2.85 (d, *J* = 7.5 Hz, 2H), 2.76 (dd, *J* = 5.0, 8.7 Hz, 1H), 2.56 (t, *J* = 7.5 Hz, 2H), 2.17–2.06 (m, 1H), 1.79–1.44 (m, 4H), 1.09 (s, 3H), 1.02 (s, 3H), 0.99–0.86 (m, 2H), 0.81 (s, 3H). ^13^C NMR (126 MHz, CDCl_3_): δ = 177.6, 156.8, 138.0, 133.5, 132.9, 129.6, 129.3 (2C), 129.1, 129.0, 127.8, 114.7 (2C), 69.5, 64.4, 51.1, 48.9, 47.2, 44.8, 36.9, 36.7, 35.8, 30.4, 26.7, 20.5, 20.1, 12.4. HRMS for C_27_H_35_O_3_N^+^ calcd 421.2612, found 421.2607 [M]^+^.

(1*R*,2*S*,4*R*)-*N*-(3-((4-(2-carboxyethyl)phenoxy)methyl)benzyl)-1,7,7-trimethylbicyclo [2.2.1]heptan-2-aminium chloride (**6d**). White solid, yield 60%. ^1^H NMR (300 MHz, CDCl_3_): δ = 7.70 (s, 1H), 7.53 (d, *J* = 7.2 Hz, 1H), 7.46–7.34 (m, 2H), 7.07 (d, *J* = 8.5 Hz, 2H), 6.84 (d, *J* = 8.5 Hz, 2H), 5.00 (s, 2H), 4.38 (d, *J* = 13.6 Hz, 1H), 3.97 (d, *J* = 13.6 Hz, 1H), 2.94 (dd, *J* = 2.5, 9.8 Hz, 1H), 2.85 (t, *J* = 7.5 Hz, 2H), 2.59 (t, *J* = 7.5 Hz, 2H), 2.13–2.00 (m, 1H), 1.99–1.85 (m, 1H), 1.78–1.64 (m, 3H), 1.49 (m, 2H), 0.97 (s, 3H), 0.83 (s, 3H), 0.65 (s, 3H). ^13^C NMR (75MHz, CDCl_3_): δ = 177.9, 156.9, 138.3, 133.0, 131.0, 129.8, 129.3 (4C), 128.3, 114.8 (2C), 69.4, 62.1, 50.2, 49.1, 48.6, 44.3, 36.2, 32.2, 29.9, 28.1, 27.3, 19.2, 18.4, 13.5. HRMS for C_27_H_35_O_3_N^+^ calcd 421.2615, found 421.2610 [M]^+^.

(1*R*,2*R,*4*R*)-*N*-(4-((3-(2-carboxyethyl)phenoxy)methyl)benzyl)-1,7,7-trimethylbicyclo [2.2.1]heptan-2-aminium chloride (**6e**). White solid, yield 62%. ^1^H NMR (400 MHz, DMSO-d_6_): δ = 12.18 (br. s., 1H), 8.93–8.72 (m, 2H), 7.71 (d, *J* = 8.1 Hz, 2H), 7.49 (d, *J* = 8.2 Hz, 2H), 7.23–7.14 (m, 1H), 6.88 (s, 1H), 6.85−6.78 (m, 2H), 5.10 (s, 2H), 4.21–4.07 (m, 2H), 2.96–2.88 (m, 1H), 2.77 (t, *J* = 7.5 Hz, 2H), 2.55–2.51 (m, 2H), 2.20–2.11 (m, 1H), 1.78–1.69 (m, 1H), 1.66–1.56 (m, 1H), 1.54–1.42 (m, 2H), 1.04–0.93 (m, 5H), 0.92 (s, 3H), 0.78 (s, 3H).^13^C NMR (126 MHz, CDCl_3_): δ = 176.4, 158.2, 142.5, 138.3, 130.8 (3C), 129.4, 127.5 (2C), 121.3, 114.9, 113.5, 69.5, 65.0, 51.1, 49.0, 47.3, 44.7, 36.6, 36.0, 35.4, 30.8, 26.6, 20.5, 20.0, 12.5. HRMS for C_27_H_35_O_3_N^+^ calcd 421.2612, found 421.2608 [M]^+^.

(1*R*,2*S*,4*R*)-*N*-(4-((3-(2-carboxyethyl)phenoxy)methyl)benzyl)-1,7,7-trimethylbicyclo [2.2.1]heptan-2-aminium chloride (**6f**). White solid, yield 51%. ^1^H NMR (300 MHz, CDCl_3_): δ = 7.54 (d, *J* = 7.8 Hz, 2H), 7.34 (d, *J* = 7.8 Hz, 2H), 7.19–7.10 (m, 1H), 6.84–6.68 (m, 3H), 6.40 (br. s., 3H), 4.96 (s, 2H), 4.16 (d, *J* = 13.2 Hz, 1H), 3.89 (d, *J* = 13.2 Hz, 1H), 3.08–2.97 (m, 1H), 2.87 (t, *J* = 7.2 Hz, 2H), 2.54 (t, *J* = 7.2 Hz, 2H), 2.08–1.91 (m, 2H), 1.80–1.36 (m, 5H), 0.97 (s, 3H), 0.84 (s, 3H), 0.72 (s, 3H). ^13^C NMR (75 MHz, CDCl_3_): δ = 177.3, 158.4, 142.8, 138.3, 130.7 (2C), 130.4, 129.4, 127.7 (2C), 121.2, 115.0, 112.9, 69.3, 62.9, 50.5, 49.2, 48.6, 44.2, 36.5, 32.5, 31.1, 27.9, 27.4, 19.3, 18.4, 13.7. HRMS for C_27_H_35_O_3_N^+^ calcd 421.2612, found 421.2606 [M]^+^.

(1*R*,2*R*,4*R*)-*N*-(3-((3-(2-carboxyethyl)phenoxy)methyl)benzyl)-1,7,7-trimethylbicyclo [2.2.1]heptan-2-aminium chloride (**6g**). White solid, yield 57%. ^1^H NMR (300 MHz, CDCl_3_): δ = 7.57 (s, 1H), 7.42–7.29 (m, 3H), 7.20–6.99 (m, 4H), 6.84 (s, 1H), 6.79–6.71 (m, 2H), 5.11 (s, 2H), 4.18–4.10 (m, 1H), 4.04–3.95 (m, 1H), 2.90–2.81 (m, 3H), 2.54 (t, *J* = 6.7 Hz, 2H), 2.14 (m, 1H), 1.82–1.48 (m, 4H), 1.10–0.99 (m, 8H), 0.82 (s, 3H). ^13^C NMR (126 MHz, CDCl_3_): δ = 176.9, 158.2, 142.8, 138.6, 132.5, 129.7, 129.4, 129.1 (2C), 128.1, 121.2, 114.5, 114.2, 69.4, 65.1, 51.2, 49.0, 47.2, 44.8, 36.8, 35.9, 35.8, 30.7, 26.7, 20.5, 20.1, 12.3. HRMS for C_27_H_35_O_3_N^+^ calcd 421.2612, found 421.2615 [M]^+^.

(1*R*,2*S*,4*R*)-*N*-(3-((3-(2-carboxyethyl)phenoxy)methyl)benzyl)-1,7,7-trimethylbicyclo [2.2.1]heptan-2-aminium chloride (**6h**). White solid, yield 61%. ^1^H NMR (300 MHz, CDCl_3_): δ = 7.50 (s, 1H), 7.32–7.18 (m, 3H), 7.05 (t, *J* = 7.8 Hz, 1H), 6.82 (br. s., 1H), 6.68 (d, *J* = 7.7 Hz, 2H), 5.84 (br. s., 3H), 5.00 (s, 2H), 3.84 (d, *J* = 12.9 Hz, 1H), 3.68 (d, *J* = 13.0 Hz, 1H), 2.96 (dd, *J* = 2.5, 9.6 Hz, 1H), 2.85 (t, *J* = 6.8 Hz, 2H), 2.47 (t, *J* = 6.8 Hz, 2H), 2.10–1.98 (m, 1H), 1.91–1.79 (m, 1H), 1.75–1.60 (m, 2H), 1.42–1.29 (m, 2H), 1.18 (dd, *J* = 3.7, 13.1 Hz, 1H), 0.90 (s, 3H), 0.83 (s, 3H), 0.77 (s, 3H). ^13^C NMR (75 MHz, CDCl_3_): δ = 158.1, 143.5, 137.9, 129.2, 129.1 (3C), 128.7, 127.6, 121.0, 113.8, 113.4, 69.0, 63.1, 51.2, 49.0, 48.5, 44.3, 37.7, 34.1, 31.4, 27.6, 27.5, 19.3, 18.4, 13.9. HRMS for C_27_H_35_O_3_N^+^ calcd 421.2612, found 421.2607 [M]^+^.

### 4.2. In Vitro

#### 4.2.1. Cell Culture

HepG2 and HEK293T cells were obtained from the “Vertebrate Cell Culture Collection” of the shared research facility at the Institute of Cytology, RAS. Cells were routinely cultured in DMEM High Glucose (Servicebio, Wuhan, China) containing 10% FBS (Sigma-Aldrich, São Paulo, Brazil), 100 μg/mL streptomycin, 100 U/mL penicillin, and 0.25 μg/mL amphotericin B (Sigma-Aldrich, St. Louis, MO, USA) at 37 °C in a 5% CO_2_ incubator (NuAire, Inc., Plymouth, MN, USA). Cells were passaged when cell fusion was over 80%, about twice a week.

#### 4.2.2. The Design of the Experiment on HepG2 Cells

Cells were seeded into a 96-well plates (TPP, Trasadingen, Switzerland) at a density of 3 × 10^5^ cells/mL in DMEM High Glucose containing 10% FBS. After culturing for 24 h, the cells were washed twice with HBSS (Gibco, Paisley, UK) and the medium was replaced with serum-free low-glucose (5.5 mM glucose) DMEM (Servicebio, Wuhan, China) with compounds **6a**–**h** at various concentrations (2.5, 5, 10, 25 μM) and **GW9508** (10, 25 μM) (CAS 1115-70-4 Cayman Chemical Company, Michigan, USA). The compounds were pre-dissolved in DMSO. The final concentration of DMSO in the cell medium was 0.1% (*v*/*v*). There were three to four replicate wells for each treatment. After 24 h, the supernatant was collected for the glucose consumption and lactate release assays, while cell viability was measured using the MTT assay.

#### 4.2.3. Glucose Consumption and Lactate Release Assays

Glucose levels were assayed using a commercial kit (Vector-Best, Novosibirsk, Russia) based on the glucose oxidase method and the Multiscan Ascent photometer (Thermo Labsystems, Helsinki, Finland). Glucose consumption was calculated by subtracting the glucose concentration in the supernatant of the blank well (DMEM without cells) from the glucose concentration in the supernatant of the well containing cells. Data were presented as the percentage of maximum glucose consumption (5.5 mM) and normalized to the control (treated with vehicle) for each plate. Meanwhile, the concentration of lactate in the supernatant was also determined using a commercial kit (Vector-Best, Novosibirsk, Russia). Lactate release data were presented as percentage of the maximum value (11 mM) and normalized to the control.

#### 4.2.4. MTT Assay for Cell Viability

A solution of MTT reagent (Thiazolyl Blue Tetrazolium Bromide; Panreac AppliChem, Darmstadt, Germany) (5 mg/mL) was added to the medium in a 1:10 volume ratio, and the mixture was incubated for 4 h at 37 °C in a CO_2_ incubator. Then, the supernatant was removed and the precipitate was dissolved with DMSO (Reagent Component, Moscow, Russia) for determination of the optical density at 570 nm (630 nm was used as the reference). Cell viability was calculated as percentage of the control group.

#### 4.2.5. In Vitro FFA1 Activation Assay

For determination of the FFAR1 activation of compounds **6a**–**h**, the FFAR1 (GPR40) Reporter Assay Kit (Cayman chemical) was used. A known FFAR1 agonist, GW9508, was used as a positive control as part of the kit. The assay kit was based on a transfection complex containing DNA constructs for FFAR1, an engineered G protein that directs Gαq activation signals to the Gαs pathway, and a cAMP response element-regulated secreted alkaline phosphatase (SEAP) reporter. HEK293T cells were seeded at a density of 5 × 10^4^ cells/well into 96-well plate coated with transfection complex in DMEM (Servicebio) containing 10% FBS (Sigma-Aldrich). After culturing for 24 h, the medium was replaced by serum-free Opti-MEM (Gibco) with the test compounds at a concentration 10 μM. After 24 h, part of the supernatant was collected to assay for SEAP activity, which was measured following the addition of a luminescence-based substrate. Luminescence was measured using a SuPerMax 3100 Multi-Mode microplate reader (Shanghai Flash Spectrum Biotechnology, Shanghai, China). The luminescence value of GW9508 was taken as 100%.

### 4.3. In Vivo

#### 4.3.1. Animals

Male CD-1 mice weighing 20–25 g were used in the experiment. Animals were obtained from the vivarium of the Institute of Cytology and Genetics of the Siberian Branch of the Russian Academy of Sciences. The mice were kept under standard vivarium conditions with free access to water and standard granulated chow in a humidity- and temperature-controlled room under a 12/12-h light–dark cycle. All manipulations with animals were carried out in strict accordance with the legislation of the Russian Federation, a decree of the Ministry of Health of the Russian Federation No. 199n of 1 April 2016, and the provisions of Directive 2010/63/EU of the European Parliament, and of the Council of the European Union of 22 September 2010 on the protection of animals used for scientific purposes. The experiment was approved by the Ethics Committee of the N.N. Vorozhtsov Institute of Organic Chemistry Siberian Branch of Russian Academy of Sciences (protocol no. P-06-09.2022-14).

#### 4.3.2. Oral Glucose Tolerance Test (OGTT)

Animals were randomized by weight and divided into groups of 6 mice after quarantine. Mice were fasted for 12 h before the test. The test compounds were introduced by oral gavage in a Tween-80–water suspension. Glucose was given orally at a dose of 2.5 g/kg 30 min after introduction of the compounds. Vildagliptin (Galvus, Novartis Farmaceutica SA, Barcelona, Spain) was used as a positive control at a dose of 10 mg/kg. Blood samples were obtained via tail incision before dosing (time 0) and at 30, 60, 90, and 120 min after the glucose load. The blood glucose concentration was evaluated with a ONE TOUCH Select blood glucose meter (LIFESCAN Inc., Milpitas, CA, USA). The area under the glycemic curve (AUC) was calculated using Tai’s model [43].

## 5. Conclusions

In this study, we proposed a new synthetic approach for structural analogs of the FFAR1 agonist **QS-528**. Seven novel compounds were synthesized, varying the positions of the substituents in the aromatic fragments as well as the configuration of the asymmetric center in the bornyl moiety. The obtained compounds did not exhibit cytotoxic properties against HepG2 cells but caused cells to increase their glucose uptake in a dose-dependent manner. The studied compounds were shown to have the ability to activate the receptor at a concentration of 10 μM. Only two compounds were active in the OGTT with CD-1 mice, which allowed us to identify privileged motifs in the molecules, that is, meta-substituted (hydroxyphenyl)propanoic acid and the exobornyl fragment.

## Figures and Tables

**Table 1:** Glucose consumption and lactate release in HepG2 cells. The data are represented as mean ± SEM.

Group		Glucose Consumption, %	Lactate Release, %
**Control**		25.00 ± 0.51	15.00 ± 0.72
**6a** (PPX)	10 μM	33.94 ± 0.24 * ^#^	26.52 ± 1.33 * ^#^
	25 μM	47.08 ± 0.87 * ^#^	37.58 ± 1.03 * ^#^
**6b** (PPN)	10 μM	30.34 ± 2.21 * ^#^	20.54 ± 0.53 * ^#^
	25 μM	38.55 ± 1.28 * ^#^	24.77 ± 0.36 * ^#^
**6c** (PMX)	10 μM	35.82 ± 1.94 * ^#^	18.72 ± 0.60 * ^#^
	25 μM	40.88 ± 1.09 * ^#^	26.56 ± 0.68 * ^#^
**6d** (PMN)	10 μM	29.94 ± 1.64 * ^#^	18.81 ± 0.40 * ^#^
	25 μM	35.16 ± 2.15 * ^#^	20.44 ± 1.89 * ^#^
**6e** (MPX)	10 μM	27.20 ± 1.79 ^#^	21.31 ± 0.70 * ^#^
	25 μM	40.03 ± 1.35 * ^#^	29.19 ± 0.71 * ^#^
**6f** (MPN)	10 μM	27.53 ± 0.60 ^#^	16.51 ± 0.41 ^#^
	25 μM	35.55 ± 0.24 * ^#^	22.25 ± 0.50 * ^#^
**6g** (MMX)	10 μM	28.16 ± 1.75 * ^#^	21.28 ± 0.69 * ^#^
	25 μM	39.73 ± 0.47 * ^#^	25.87 ± 1.07 * ^#^
**6h** (MMN)	10 μM	32.57 ± 0.73 * ^#^	15.23 ± 0.34 ^#^
	25 μM	35.55 ± 0.83 * ^#^	20.65 ± 0.31 * ^#^
**GW9508**	10 μM	44.66 ± 0.35 *	34.57 ± 0.38 *
	25 μM	54.21 ± 0.83 *	46.43 ± 0.43 *

* *p* < 0.05 as compared to control; ^#^ *p* < 0.05 as compared to GW9508 at the same dose.

**Table 2:** Hypoglycemic effect of compounds **6a**–**h** as a difference between their AUC and AUC of corresponding control group in OGTT, presented in percentage.

Compound	AUC Difference (5 mg/kg), %	AUC Difference (15 mg/kg), %
**6a** **(QS-528)**	- ^1^	-
**6b**	-	-
**6c**	-	-
**6d**	-	-
**6e** (MPX)	14.0	20.8
**6f**	-	-
**6g** (MMX)	-	23.4
**6h**	-	-
**Vildagliptin**	25.4 ^2^	

^1^ No significant difference. ^2^ At a dose 10 mg/kg.

## Data Availability

Not applicable.

## Supplementary Materials

The following supporting information can be downloaded at: https://www.mdpi.com/article/10.3390/ijms24098022/s1, ^1^H and ^13^C NMR spectra of compounds **3**–**6** (Figures S1–S63). Synthetic procedures for the compounds **1a,b**, **2a,b** and bornylamines.

## References

[1] Saeedi P., Petersohn I., Salpea P., Malanda B., Karuranga S., Unwin N., Colagiuri S., Guariguata L., Motala A.A., Ogurtsova K. (2019). IDF Diabetes Atlas Committee. Global and regional diabetes prevalence estimates for 2019 and projections for 2030 and 2045: Results from the International Diabetes Federation Diabetes Atlas, 9th edition. Diabetes Res. Clin. Pract..

[2] Khan M.A.B., Hashim M.J., King J.K., Govender R.D., Mustafa H., Kaabi J.A. (2020). Epidemiology of Type 2 Diabetes—Global Burden of Disease and Forecasted Trends. J. Epidemiol. Glob. Health.

[3] Roden M., Shulman G.I. (2019). The integrative biology of type 2 diabetes. Nature.

[4] Galicia-Garcia U., Benito-Vicente A., Jebari S., Larrea-Sebal A., Siddiqi H., Uribe K.B., Ostolaza H., Martín C. (2020). Pathophysiology of Type 2 Diabetes Mellitus. Int. J. Mol. Sci..

[5] Hu Q., Chen Y., Deng X., Li Y., Ma X., Zeng J., Zhao Y. (2023). Diabetic nephropathy: Focusing on pathological signals, clinical treatment, and dietary regulation. Biomed. Pharmacother..

[6] Scanlon P.H. (2022). Diabetic retinopathy. Medicine.

[7] Laakso M. (1999). Hyperglycemia and cardiovascular disease in type 2 diabetes. Diabetes.

[8] Srinivasan B.T., Davies M. (2019). Glycaemic management of type 2 diabetes. Medicine.

[9] Fuh M.T., Tseng C.C., Li S.M., Tsai S.E., Chuang T.J., Lu C.H., Yang Y.C., Tsai H.J., Wong F.F. (2021). Design, synthesis and biological evaluation of glycolamide, glycinamide, and β-amino carbonyl 1,2,4-triazole derivatives as DPP-4 inhibitors. Bioorg. Chem..

[10] Vo D.V., Hong K.H., Lee J., Park H. (2021). Synthesis, in vitro evaluation, and computational simulations studies of 1,2,3-triazole analogues as DPP-4 inhibitors. Bioorg. Med. Chem..

[11] Sun J., Liu H.Y., Zhang Y.H., Fang Z.Y., Lv P.C. (2021). Design, synthesis and bioactivity evaluation of thiazolidinedione derivatives as partial agonists targeting PPARγ. Bioorg. Chem..

[12] Shakour N., Sahebkar A., Karimi G., Paseban M., Tasbandi A., Mosaffa F., Tayarani-Najaran Z., Ghodsi R., Hadizadeh F. (2021). Design, synthesis and biological evaluation of novel 5-(imidazolyl-methyl) thiazolidinediones as antidiabetic agents. Bioorg. Chem..

[13] Tseng P.S., Ande C., Moremen K.W., Crich D. (2023). Influence of Side Chain Conformation on the Activity of Glycosidase. Inhibitors. Angew. Chem. Int. Ed..

[14] Rajasekaran P., Ande C., Vankar Y.D. (2022). Synthesis of (5,6 & 6,6)-oxa-oxa annulated sugars as glycosidase inhibitors from 2-formyl galactal using iodocyclization as a key step. Arkivoc.

[15] Chennaiah A., Bhowmick S., Vankar Y.D. (2017). Conversion of glycals into vicinal-1,2-diazides and 1,2-(or 2,1)-azidoacetates using hypervalent iodine reagents and Me*_3_*SiN*_3_*. Application in the synthesis of N-glycopeptides, pseudo-trisaccharides and an iminosugar. RSC Adv..

[16] Putapatri S.R., Kanwal A., Banerjee S.K., Kantevari S. (2014). Synthesis of novel l-rhamnose derived acyclic C-nucleosides with substituted 1,2,3-triazole core as potent sodium-glucose co-transporter (SGLT) inhibitors. Bioorg. Med. Chem. Lett..

[17] Xu G., Gaul M.D., Kuo G.H., Du F., Xu J.Z., Wallace N., Hinke S., Kirchner T., Silva J., Huebert N.D. (2018). Design, synthesis and biological evaluation of (2S,3R,4R,5S,6R)-5-fluoro-6-(hydroxymethyl)-2-aryltetrahydro-2H-pyran-3,4-diols as potent and orally active SGLT dual inhibitors. Bioorg. Med. Chem. Lett..

[18] Poitout V., Lin D.C. (2013). Modulating GPR40: Therapeutic promise and potential in diabetes. Drug Discov. Today.

[19] Prentki M., Tornheim K., Corkey B.E. (1997). Signal transduction mechanisms in nutrient-induced insulin secretion. Diabetologia.

[20] Kolczynska K., Loza-Valdes A., Hawro I., Sumara G. (2020). Diacylglycerol-evoked activation of PKC and PKD isoforms in regulation of glucose and lipid metabolism: A review. Lipids Health Dis..

[21] Governa P., Caroleo M.C., Carullo G., Aiello F., Cione E., Manetti F. (2021). FFAR1/GPR40: One target, different binding sites, many agonists, no drugs, but a continuous and unprofitable tug-of-war between ligand lipophilicity, activity, and toxicity. Bioorg. Med. Chem. Lett..

[22] Briscoe C.P., Tadayyon M., Andrews J.L., Benson W.G., Chambers J.K., Eilert M.M., Ellis C., Elshourbagy N.A., Goetz A.S., Minnick D.T. (2003). The orphan G protein-coupled receptor GPR40 is activated by medium and long chain fatty acids. J. Biol. Chem..

[23] Kuranov S.O., Luzina O.A., Salakhutdinov N.F. (2020). FFA1 (GPR40) Receptor Agonists Based on Phenylpropanoic Acid as Hypoglycemic Agents: Structure–Activity Relationship. Russ. J. Bioorg. Chem..

[24] Kuranov S.O., Luzina O.A., Onopchenko O., Pishel I., Zozulya S., Gureev M., Salakhutdinov N.F., Krasavin M. (2020). Exploring bulky natural and natural-like periphery in the design of p-(benzyloxy)phenylpropionic acid agonists of free fatty acid receptor 1 (GPR40). Bioorg. Chem..

[25] Kuranov S., Luzina O., Khvostov M., Baev D., Kuznetsova D., Zhukova N., Vassiliev P., Kochetkov A., Tolstikova T., Salakhutdinov N. (2020). Bornyl Derivatives of *p*-(Benzyloxy)Phenylpropionic Acid: In Vivo Evaluation of Antidiabetic Activity. Pharmaceuticals.

[26] Pon`kina D., Kuranov S., Khvostov M., Zhukova N., Meshkova Y., Marenina M., Luzina O., Tolstikova T., Salakhutdinov N. (2023). Hepatoprotective Effect of a New FFAR1 Agonist—N-Alkylated Isobornylamine. Molecules.

[27] Plummer C.W., Clements M.J., Chen H., Rajagopalan M., Josien H., Hagmann W.K., Miller M., Trujillo M.E., Kirkland M., Kosinski D. (2017). Design and Synthesis of Novel, Selective GPR40 AgoPAMs. ACS Med. Chem. Lett..

[28] Edfalk S., Steneberg P., Edlund H. (2008). GPR40 is expressed in enteroendocrine cells and mediates free fatty acid stimulation of incretin secretion. Diabetes.

[29] Mitsunobu O. (1981). The Use of Diethyl Azodicarboxylate and Triphenylphosphine in Synthesis and Transformation of Natural Products. Synthesis.

[30] Hamdouchi C., Kahl S.D., Patel Lewis A., Cardona G.R., Zink R.W., Chen K., Eessalu T.E., Ficorilli J.V., Marcelo M.C., Otto K.A. (2016). The Discovery, Preclinical, and Early Clinical Development of Potent and Selective GPR40 Agonists for the Treatment of Type 2 Diabetes Mellitus (LY2881835, LY2922083, and LY2922470). J. Med. Chem..

[31] Carullo G., Mazzotta S., Vega-Holm M., Iglesias-Guerra F., Vega-Pérez J.M., Aiello F., Brizzi A. (2021). GPR120/FFAR4 Pharmacology: Focus on Agonists in Type 2 Diabetes Mellitus Drug Discovery. J. Med. Chem..

[32] Nevin D.K., Lloyd D.G., Fayne D. (2011). Rational targeting of peroxisome proliferating activated receptor subtypes. Curr. Med. Chem..

[33] Miyachi H., Uchiki H. (2003). Analysis of the critical structural determinant(s) of species-selective peroxisome proliferator-activated receptor alpha (PPAR alpha)-activation by phenylpropanoic acid-type PPAR alpha agonists. Bioorg. Med. Chem. Lett..

[34] Colín-Lozano B., Estrada-Soto S., Chávez-Silva F., Gutiérrez-Hernández A., Cerón-Romero L., Giacoman-Martínez A., Almanza-Pérez J.C., Hernández-Núñez E., Wang Z., Xie X. (2018). Design, Synthesis and in Combo Antidiabetic Bioevaluation of Multitarget Phenylpropanoic Acids. Molecules.

[35] Tang Y.B., Liu J.Z., Zhang S.E., Du X., Nie F., Tian J.Y., Ye F., Huang K., Hu J.P., Li Y. (2014). 3-Phenylpropanoic acid-based phosphotyrosine (pTyr) mimetics: Hit evolution to a novel orally active protein tyrosine phosphatase 1B (PTP1B) inhibitor. ChemMedChem.

[36] Bianchini G., Nigro C., Sirico A., Novelli R., Prevenzano I., Miele C., Beguinot F., Aramini A. (2021). A new synthetic dual agonist of GPR120/GPR40 induces GLP-1 secretion and improves glucose homeostasis in mice. Biomed. Pharmacother..

[37] Hidalgo-Figueroa S., Rodríguez-Luévano A., Almanza-Pérez J.C., Giacoman-Martínez A., Ortiz-Andrade R., León-Rivera I., Navarrete-Vázquez G. (2021). Synthesis, molecular docking, dynamic simulation and pharmacological characterization of potent multifunctional agent (dual GPR40-PPARγ agonist) for the treatment of experimental type 2 diabetes. Eur. J. Pharmacol..

[38] Sanchez M.B., Miranda-Perez E., Verjan J.C.G., de Los Angeles Fortis Barrera M., Perez-Ramos J., Alarcon-Aguilar F.J. (2017). Potential of the chlorogenic acid as multitarget agent: Insulin-secretagogue and PPAR α/γ dual agonist. Biomed. Pharmacother..

[39] Bhurruth-Alcor Y., Røst T., Jorgensen M.R., Kontogiorgis C., Skorve J., Cooper R.G., Sheridan J.M., Hamilton W.D., Heal J.R., Berge R.K. (2011). Synthesis of novel PPARα/γ dual agonists as potential drugs for the treatment of the metabolic syndrome and diabetes type II designed using a new de novo design program protobuild. Org. Biomol. Chem..

[40] Brown S.P., Dransfield P.J., Vimolratana M., Jiao X., Zhu L., Pattaropong V., Sun Y., Liu J., Luo J., Zhang J. (2012). Discovery of AM-1638: A Potent and Orally Bioavailable GPR40/FFA1 Full Agonist. ACS Med. Chem. Lett..

[41] Yin J., Hu R., Chen M., Tang J., Li F., Yang Y., Chen J. (2002). Effects of berberine on glucose metabolism in vitro. Metab. Clin. Exp..

[42] Cheng Z., Pang T., Gu M., Gao A.H., Xie C.M., Li J.Y., Nan F.J., Li J. (2006). Berberine-stimulated glucose uptake in L6 myotubes involves both AMPK and p38 MAPK. Biochim. Biophys. Acta.

[43] Tai M.M. (1994). A mathematical model for the determination of total area under glucose tolerance and other metabolic curves. Diabetes Care.

