# Supplementary material for: Synthesis and Evaluation of Hypoglycemic Activity of Structural Isomers of ((Benzyloxy)phenyl)propanoic Acid Bearing an Aminobornyl Moiety
Source: Int J Mol Sci. 2023 Apr 28;24(9):8022. doi: 10.3390/ijms24098022 (PMC10178661; doi:10.3390/ijms24098022)
Supplement: Supplementary file 1 [file ijms-24-08022-s001.zip › ijms-2356190-supplementary.pdf]

Synthesis and Evaluation of Hypoglycemic Activity of Structural Isomers of ((Benzyloxy)phenyl)propanoic Acid Bearing an Aminobornyl Moiety

Sergey O. Kuranov, Darya A. Pon`kina, Yulia V. Meshkova, Mariya K. Marenina, Mikhail V. Khvostov, Olga A. Luzina, Tatiana G. Tolstikova and Nariman F. Salakhutdinov

Content

Synthesis of compounds 1a,b 6
General procedure for dimethyl acetal synthesis 6
1-bromo-4-(dimethoxymethyl)benzene 6
1-bromo-3-(dimethoxymethyl)benzene 6
General procedure for bromoarene formylation 6
4-(dimethoxymethyl)benzaldehyde 7
3-(dimethoxymethyl)benzaldehyde 7
General procedure for aldehyde reduction 7
(4-(Dimethoxymethyl)phenyl)methanol 7
(3-(Dimethoxymethyl)phenyl)methanol 7
Synthesis of compounds 2a,b 7
General procedure for Knoevenagel-Doebner reaction 8
(E)-3-(4-hydroxyphenyl)acrylic acid 8
(E)-3-(3-hydroxyphenyl)acrylic acid 8

General procedure for cinnamic acids hydrogenation 8
3-(4-Hydroxyphenyl)propanoic acid 8
3-(3-Hydroxyphenyl)propanoic acid 9
General procedure for esterification of (hydroxyphenyl)propanoic acid 9
Methyl 3-(4-hydroxyphenyl)propanoate 9
Methyl 3-(3-hydroxyphenyl)propanoate 9
Synthesis of bornylamines 9
Camphor oxime 9
Nitroimine 10
Camphor imine 10
Bornylamine 10
(1R,2R,4R)-1,7,7-trimethylbicyclo[2.2.1]heptan-2-amine (Exobornylamine) 10
(1R,2S,4R)-1,7,7-trimethylbicyclo[2.2.1]heptan-2-amine (Endobornylamine) 11
^{1}H and ^{13}C NMR spectra of compounds 3-6 12
Figure S1. ^{1}H NMR spectrum of 3a 12
Figure S2. ^{13}C NMR spectrum of 3a 13
Figure S3. ^{13}C NMR spectrum of 3a (JMOD) 14
Figure S4. ^{1}H NMR spectrum of 3b 15
Figure S5. ^{13}C NMR spectrum of 3b 16
Figure S6. ^{13}C NMR spectrum of 3b. (JMOD) 17
Figure S7. ^{1}H NMR spectrum of 3c 18
Figure S8. ^{13}C NMR spectrum of 3c 19

Figure S9. ^{13}C NMR spectrum of 3c (JMOD)....20
Figure S10. ^{1}H NMR spectrum of 3d....21
Figure S11. ^{13}C NMR spectrum of 3d....22
Figure S12. ^{13}C NMR spectrum of 3d (JMOD)....23
Figure S13. ^{1}H NMR spectrum of 4a....24
Figure S14. ^{1}H NMR spectrum of 4b....25
Figure S15. ^{1}H NMR spectrum of 4c....26
Figure S16. ^{1}H NMR spectrum of 4d....27
Figure S17. ^{1}H NMR spectrum of 5a....28
Figure S18. ^{13}C NMR spectrum of 5a (JMOD)....29
Figure S19. ^{1}H NMR spectrum of 5b....30
Figure S20. ^{13}C NMR spectrum of 5b....31
Figure S21. ^{13}C NMR spectrum of 5b (JMOD)....32
Figure S22. ^{1}H NMR spectrum of 5c....33
Figure S23. ^{13}C NMR spectrum of 5c....34
Figure S24. ^{13}C NMR spectrum of 5c (JMOD)....35
Figure S25. ^{1}H NMR spectrum of 5d....36
Figure S26. ^{13}C NMR spectrum of 5d....37
Figure S27. ^{13}C NMR spectrum of 5d (JMOD)....38
Figure S28. ^{1}H NMR spectrum of 5e....39
Figure S29. ^{13}C NMR spectrum of 5e....40
Figure S30. ^{13}C NMR spectrum of 5e (JMOD)....41

Figure S31. ^{1}H NMR spectrum of 5f....................42
Figure S32. ^{13}C NMR spectrum of 5f....................43
Figure S33. ^{13}C NMR spectrum of 5f (JMOD)....................44
Figure S32. ^{1}H NMR spectrum of 5g....................45
Figure S35. ^{13}C NMR spectrum of 5g....................46
Figure S36. ^{13}C NMR spectrum of 5g (JMOD)....................47
Figure S37. ^{1}H NMR spectrum of 5h....................48
Figure S38. ^{13}C NMR spectrum of 5h....................49
Figure S39. ^{13}C NMR spectrum of 5h (JMOD)....................50
Figure S40. ^{1}H NMR spectrum of 6a....................51
Figure S41. ^{13}C NMR spectrum of 6a....................52
Figure S42. ^{13}C NMR spectrum of 6a (JMOD)....................53
Figure S43. ^{1}H NMR spectrum of 6b....................54
Figure S44. ^{13}C NMR spectrum of 6b....................55
Figure S45. ^{13}C NMR spectrum of 6b (JMOD)....................56
Figure S46. ^{1}H NMR spectrum of 6c....................57
Figure S47. ^{13}C NMR spectrum of 6c....................58
Figure S48. ^{13}C NMR spectrum of 6c (JMOD)....................59
Figure S49. ^{1}H NMR spectrum of 6d....................60
Figure S50. ^{13}C NMR spectrum of 6d....................61
Figure S51. ^{13}C NMR spectrum of 6d (JMOD)....................62
Figure S52. ^{1}H NMR spectrum of 6e....................63

Figure S53. ^{13}C NMR spectrum of 6e.......64
Figure S54. ^{13}C NMR spectrum of 6e (JMOD).......65
Figure S55. ^{1}H NMR spectrum of 6f.......66
Figure S56. ^{13}C NMR spectrum of 6f.......67
Figure S57. ^{13}C NMR spectrum of 6f (JMOD).......68
Figure S58. ^{1}H NMR spectrum of 6g.......69
Figure S59. ^{13}C NMR spectrum of 6g.......70
Figure S60. ^{13}C NMR spectrum of 6g (JMOD).......71
Figure S61. ^{1}H NMR spectrum of 6h.......72
Figure S62. ^{13}C NMR spectrum of 6h.......73
Figure S63. ^{13}C NMR spectrum of 6h.......74

Synthesis of compounds 1a,b

General procedure for dimethyl acetal synthesis

Bromoaldehyde (33.3 g, 180.0 mmol) and trimethyl orthoformate (40 mL, 366.0 mmol) were dissolved in 200 mL of anhydrous methanol. To the obtained stirred solution paratoluenesulfonic acid monohydrate (0.5 g, 2.6 mmol) was added. After 30 minutes of stirring the reaction was termindated by adding triethylamine (2 mL, 27.1 mmol). Methanol and excess of triethylamine were distilled off, and the residue was purified by vacuum distillation.

1-bromo-4-(dimethoxymethyl)benzene

Colorless liquid, 87%. Bp 97°C at 4 mm Hg. ^{1}H NMR (400 MHz, $CDCl_3$): δ = 7.47 - 7.52 (m, 2H, m), 7.30 - 7.35 (m, 2H), 5.36 (s, 1H), 3.30 (s, 6H). ^{13}C NMR (101 MHz, $CDCl_3$): δ = 137.1, 131.3, 128.5, 122.4, 102.2, 52.5.

1-bromo-3-(dimethoxymethyl)benzene

Colorless liquid, 85%. Bp 112°C at 8 mm Hg. ^{1}H NMR (600 MHz, $CDCl_3$): δ = 7.62 (s, 1 H), 7.45 (dd, J = 1.7, 8.1 Hz, 1H), 7.37 (d, J = 7.5 Hz, 1H), 7.25 - 7.22 (m, 1H), 5.36 (s, 1H), 3.32 (s, 6H). ^{13}C NMR (151 MHz, $CDCl_3$): δ = 140.4, 131.5, 129.9, 129.8, 125.4, 122.4, 102.0, 52.6.

General procedure for bromoarene formylation

A 100 ml four-necked round-bottom flask with magnetic stirrer was flame dried and cooled in a desiccator over phosphorus pentoxide. The flask was equipped with an argon inlet, a pressure equalizing dropping funnel with PTFE stopcock connected to an outlet to the bubbler with mineral oil, thermometer and rubber septum. A stream of argon is passed through the reaction vessel until the synthesis is complete. After several minutes of purging the system with argon **bromo(dimethoxymethyl)benzene** (7.87 g, 34.0 mmol) and freshly-distilled tetrahydrofuran (40 mL) were added to the flask and n-butyllithium 2.5M solution in hexane (16 mL, 41.0 mmol) was added to the dropping funnel in a countercurrent of argon. The intensively stirred solution was cooled to -78°C then n-butyllithium solution in hexane was added drop-wise for about 30 minutes without allowing the temperature to rise above -60 °C. After 20 minutes, dry DMF was added drop wise to the obtained solution through a septum using a syringe without allowing the temperature of the reaction mixture to rise above -60°C. After 30 minutes from the start of the addition of DMF, cooling was removed and the reaction mixture was allowed to warm up to -10°C then poured in a 5% NH_4Cl solution (150 mL) and ethyl acetate * (with 1% v/v of triethylamine) (60 mL). The organic layer was separated and the aqueous layer was extracted with ethyl acetate* (with 1% v/v of triethylamine) (2 x 60 mL). The combined organic extracts were washed with 10% sodium bicarbonate solution (30 mL), brine (30 mL) and dried over anhydrous sodium sulfate overnight. The drying agent was filtered off and washed

with a small portion of ethyl acetate* (with 1% v/v of triethylamine). The combined solutions were evaporated under reduced pressure and the residue was used in the next step without further purification.

4-(dimethoxymethyl)benzaldehyde

^{1}H NMR (400 MHz, $CDCl_3$): δ = 10.03 (s, 1H), 7.89 (d, *J* = 8.3 Hz, 2H), 7.63 (d, *J* = 8.3 Hz, 2H), 5.45 (s, 1H), 3.34 (s, 6H).

3-(dimethoxymethyl)benzaldehyde

^{1}H NMR (400 MHz, $CDCl_3$): δ = 10.03 (s, 1H), 7.98 (s, 1H), 7.85 (d, *J* = 7.5 Hz, 1H), 7.72 (d, *J* = 7.7 Hz, 1H), 7.57 - 7.51 (m, 1H), 5.46 (s, 1H), 3.33 (s, 6H).

General procedure for aldehyde reduction

To an ice-cooled solution of crude **(dimethoxymethyl)benzaldehyde** (34 mmol, based on previous step) in methanol (70 mL) sodium borohydride (1.55 g, 40.8 mmol) was added portion wise in 30 minutes. After two hours an aqueous ammonia solution (2 mL) was added and the obtained solution was concentrated under reduced pressure. To the residue an aqueous ammonia solution (60 mL) and then ethyl acetate* (50 mL) was added. The organic layer was separated and the aqueous layer was extracted with ethyl acetate* (with 1% v/v of triethylamine) (2 x 50 mL). The combined organic extracts were washed with brine (30 mL) and dried over anhydrous sodium sulfate overnight. The drying agent was filtered off and washed with a small portion of ethyl acetate (with 1% v/v of triethylamine)*. The combined solutions were evaporated under reduced pressure and the residue was purified over silica gel column chromatography using hexane-ethyl acetate-triethylamine 100:20:1 as eluent.

(4-(Dimethoxymethyl)phenyl)methanol

Colorless oil, 72%. ^{1}H NMR (400 MHz, $CDCl_3$): δ = 7.44 - 7.40 (m, 2H), 7.37 - 7.32 (m, 2H), 5.38 (s, 1H), 4.66 (br. s., 2H), 3.31 (s, 6H), 2.20 (br. s., 1H). ^{13}C NMR (126MHz, $CDCl_3$): δ = 141.1, 137.2, 126.8, 126.7, 102.8, 64.8, 52.6.

(3-(Dimethoxymethyl)phenyl)methanol

Colorless oil, 68%. ^{1}H NMR (300MHz, $CDCl_3$): δ = 7.45 (s, 1 H), 7.41 - 7.33 (m, 3 H), 5.38 (s, 1 H), 4.70 (d, *J* = 5.7 Hz, 2 H), 3.33 (s, 6 H). ^{13}C NMR (126MHz, $CDCl_3$): δ = 140.9, 138.3, 128.5, 127.0, 125.9, 125.1, 103.0, 65.2, 52.8.

Synthesis of compounds 2a,b

General procedure for Knoevenagel-Doebner reaction

3- or 4-Hydroxybenzaldehyde (1.512 g, 12.3 mmol) and malonic acid (2.83 g, 27.2 mmol) were placed in a 25 ml flask and dissolved in 6.8 ml of pyridine. Piperidine (0.115 ml, 1.2 mmol) was added, and the reaction was placed in pre-heated to 67 °C bath for 5 days (controlled by TLC CH_2Cl_2-ethyl acetate 4:1). Then, the mixture was transferred to a beaker, and 100 ml of water was added. Concentrated HCl was added drop-wise until pH 2-3. A precipitate was filtered off, washed with distilled water and dried in a vacuum desiccator over phosphorus pentoxide.

(E)-3-(4-hydroxyphenyl)acrylic acid
White solid, 83%. ^{1}H NMR (400 MHz ,DMSO-d_6): δ = 12.14 (br. s., 1H), 9.97 (br. s., 1H), 7.57 - 7.45 (m, 3H), 6.78 (d, *J* = 8.3 Hz, 2H), 6.29 (d, *J* = 15.8 Hz, 1H).

(E)-3-(3-hydroxyphenyl)acrylic acid
White solid, 57%. ^{1}H NMR (400 MHz, DMSO-d6): δ = 12.39 (br. s., 1H), 9.62 (br. s., 1H), 7.49 (d, *J* = 16.0 Hz, 1H), 7.17 - 7.26 (m, 1H), 7.10 (d, *J* = 7.7 Hz, 1H), 7.01 (s, 1H), 6.82 (dd, *J* = 8.0, 1.7 Hz, 1H), 6.40 (d, *J* = 16.0 Hz, 1H).

General procedure for cinnamic acids hydrogenation

A solution of **3-(hydroxyphenyl)acrylic acid** (11.815 g, 72.0 mmol) and 10% Pd/C (1.82 g, 10 wt%) in methanol (120 mL) in a 500 mL flask was cooled in an ice bath. The air was evacuated in a vacuum then hydrogen gas filled the system. The reaction vessel was degassed two times, replacing each time the vacuum by hydrogen. The reaction mixture connected with balloon filled with hydrogen was stirred at room temperature overnight. A reaction was cooled in an ice bath, then unreacted hydrogen was removed under vacuum and the flask was filled with argon. Pd/C was filtered off and washed with small amount of methanol (10-20 mL). Combined methanol solutions were combined and used in the next step.

3-(4-Hydroxyphenyl)propanoic acid
^{1}H NMR (400 MHz, DMSO-d_6): δ = 12.08 (br. s., 1H), 9.17 (br. s., 1H), 7.00 (d, J = 8.5 Hz, 2H), 6.65 (d, J = 8.5 Hz, 2H), 2.69 (t, J = 7.6, 2H), 2.40 - 2.48 (m, 2H).

3-(3-Hydroxyphenyl)propanoic acid
^{1}H NMR (400 MHz, DMSO-d$_6$): δ = 12.15 (br. s, 1H), 9.28 (br. s, 1H), 7.05 (t, *J* = 7.7, 1H), 6.64 - 6.54 (m, 3H), 2.72 (t, *J* = 7.7 Hz, 11 H), 2.49 - 2.44 (m, 2 H).

General procedure for esterification of (hydroxyphenyl)propanoic acid

To a solution of **(hydroxyphenyl)propanoic acid** obtained after hydrogenation 2-3 drops of conc. sulfuric acid were added and the solution obtained was refluxed for a several hours until disappearing of starting material (controlled by TLC CH_2Cl_2-acetic acid 100:1). After completion of the reaction methanol was evaporated under vacuum. The residue was re-dissolved in ethyl acetate (40 mL) and washed with water (10 mL), sodium bicarbonate solution (10 mL), brine (10 mL) and dried over magnesium sulfate. The drying agent was filtered off and washed with a small portion of ethyl acetate. The combined ethyl acetate solutions were evaporated under reduced pressure and the residue was purified over silica gel column chromatography using hexane-ethyl acetate 7:3 (v/v) as eluent.

Methyl 3-(4-hydroxyphenyl)propanoate
Yellow oil, 81%. ^{1}H NMR (400 MHz, $CDCl_3$): δ = 7.03 (d, J = 8.5, 2H), 6.73 - 6.81 (d, J = 8.5, 2H), 6.19 (br. s., 1H), 3.68 (s, 3H), 2.88 (t, J = 7.7, 2H), 2.58 - 2.65 (t, J = 7.7, 2H).

Methyl 3-(3-hydroxyphenyl)propanoate
Yellow oil, 83%. ^{1}H NMR (400 MHz, $CDCl_3$): δ = 7.18 - 7.12 (m, 1H), 6.77 - 6.66 (m, 3H), 5.64 (s, 1H), 3.68 (s, 3H), 2.90 (t, *J* = 7.7 Hz, 2H), 2.63 (t, *J* = 7.7 Hz, 2H)

Synthesis of bornylamines

Camphor oxime

Hydroxylamine hydrochloride (22.36 g, 0.32 mol), (1*R*)-(+)-camphor (22.38 g, 0.15 mol) and pyridine (18 mL, 0.22 mol) were heated under reflux in ethanol (220 mL) for 5 hours. After cooling, most of the ethanol in the reaction mixture was removed *in vacuo*. Water was then added, causing crude oxime to precipitate from the solution as colorless crystals, which were isolated by filtration and washed with distilled water. The crystalline material was collected, dried under vacuum and recrystallized from ethanol. Colorless crystals, yield 62%.

^{1}H NMR (400 MHz, DMSO-d$_6$): δ = 10.02 (s, 1H), 2.35 (td, *J* = 3.7, 17.5 Hz, 1H), 1.86 - 1.82 (m, 1H), 1.81 - 1.71 (m, 1H), 1.70 - 1.61 (m, 1H), 1.33 - 1.25 (m, 1H), 1.21 - 1.13 (m, 1H), 0.91 (s, 3H), 0.87 (s, 3H), 0.71 (s, 3H)

Nitroimine

Camphor oxime (12.30 g, 73 mmol) in glacial acetic acid (360 mL) was treated with 5% aqueous sodium nitrite solution (180 mL). A bright yellow color developed and dispersed over 30 minutes. After a further 1.5 hours, the crude product was precipitated as a colorless solid by the addition of water and isolated by filtration. After drying under vacuum, the crude product was recrystallized from ethanol to afford camphor nitroimine. White solid, yield 76%.

^{1}H NMR (400 MHz, $CDCl_3$) δ = 2.73 - 2.64 (m, 1H), 2.13 (d, *J* = 18.5 Hz, 1H), 2.03 (t, *J* = 4.3 Hz, 1H), 1.97 - 1.80 (m, 2H), 1.60 - 1.52 (m, 1H), 1.36 - 1.28 (m, 1H), 1.04 (s, 3H), 0.98 (s, 3H), 0.88 (s, 3H)

Camphor imine

A solution of **camphor nitroimine** (11.0 g, 56 mmol) in dry tetrahydrofuran (100 mL) was treated at 0°C with a slow stream of ammonia gas for 4 hours. The solvent was removed *in vacuo* (keeping the bath below 30 °C) to give the imine as a pale yellow solid which was used immediately in the next step.

Bornylamine

To an ice-cooled solution of crude **camphor imine** (56 mmol, based on nitroimine load in previous step) in methanol (120 mL) sodium borohydride (3.5 g, 92.1 mmol) was added portion-wise in 30 minutes. After ten hours water (2 mL) was added and the resulting solution was concentrated under reduced pressure. To the residue an aqueous 5% ammonium chloride solution (60) and chloroform (50) were added. The organic layer was separated and the aqueous layer was extracted with chloroform (2 x 50 mL). The combined organic extracts were washed with water (30 mL), brine (30 mL) and dried over anhydrous sodium sulfate overnight. The drying agent was filtered off and washed with a small portion of chloroform. The combined solutions were evaporated under reduced pressure and the residue was purified over silica gel column chromatography using chloroform-ethanol-$NH_{3(aq)}$ 100:5:1 as eluent.

(1R,2R,4R)-1,7,7-trimethylbicyclo[2.2.1]heptan-2-amine (Exobornylamine)

Slightly yellow oil, yield 38%. ^{1}H NMR (300 MHz, $CDCl_3$): δ = 2.68 (dd, *J* = 5.1, 8.6 Hz, 1H), 1.78 - 1.59 (m, 3H), 1.57 - 1.43 (m, 2H), 1.34 (br. s., 2H), 1.12 - 0.91 (m, 5H), 0.84 (s, 3H), 0.79 (s, 3H). ^{13}C NMR (75MHz, $CDCl_3$): δ = 60.4, 48.0, 46.5, 44.9, 40.5, 36.3, 27.2, 20.8, 20.3, 11.8.

(1R,2S,4R)-1,7,7-trimethylbicyclo[2.2.1]heptan-2-amine (Endobornylamine)

Slightly yellow oil, yield 10%. ^{1}H NMR (400 MHz, $CDCl_3$): δ = 3.03 (ddd, *J* = 1.9, 4.5, 10.6 Hz, 1H), 2.28 - 2.18 (m, 1H), 1.78 - 1.60 (m, 4H), 1.56 (t, *J* = 4.4 Hz, 1H), 1.30 - 1.08 (m, 2H), 0.88 - 0.80 (m, 6H), 0.76 (s, 3H), 0.70 (dd, *J* = 4.5, 13.1 Hz, 1H). ^{13}C NMR (126 MHz, $CDCl_3$): δ = 56.6, 49.0, 48.2, 44.9, 39.4, 28.4, 26.3, 20.3, 18.4, 13.3

^{1}H and ^{13}C NMR spectra of compounds 3-6

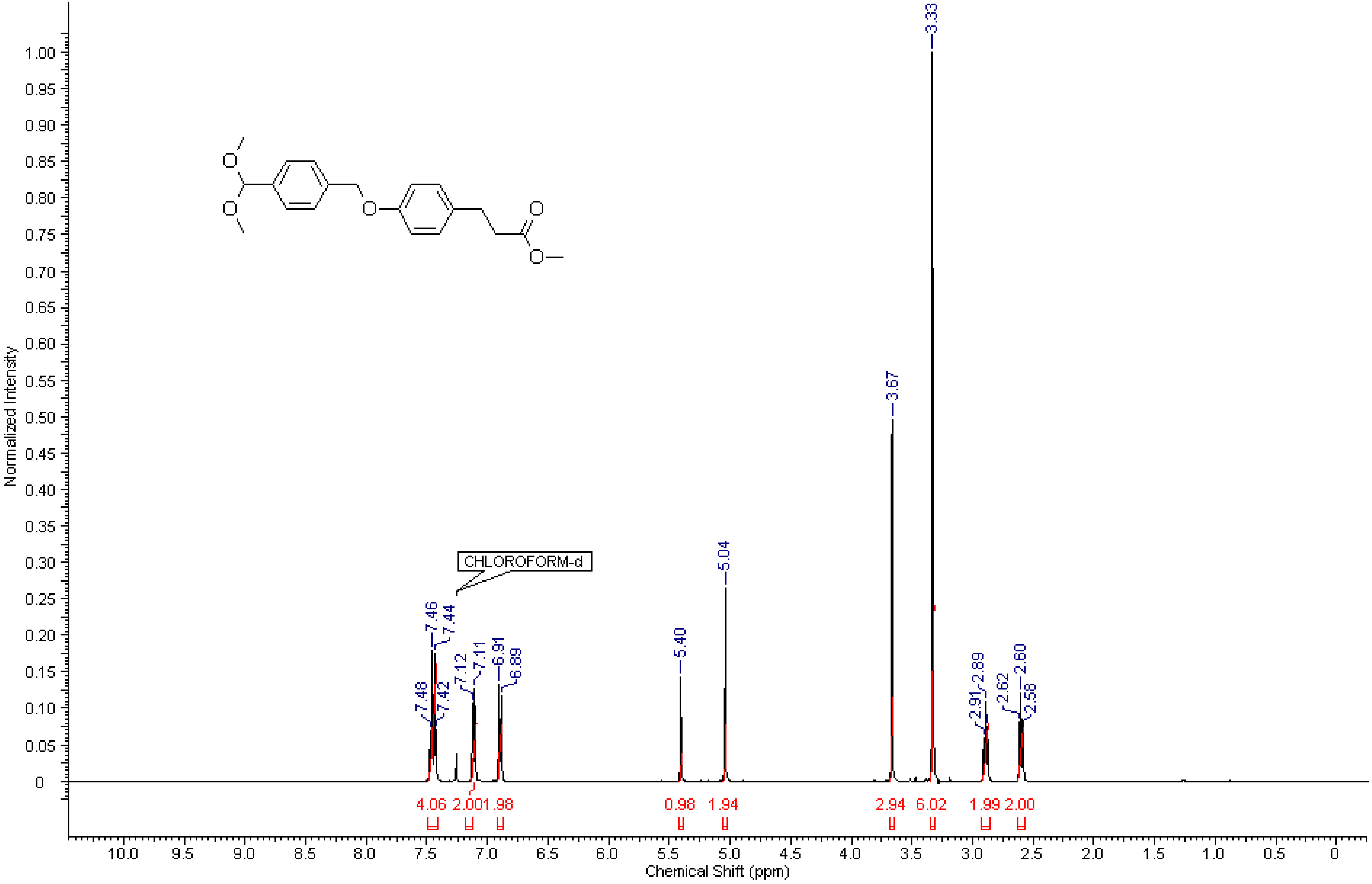

Figure S1. **^{1}H NMR spectrum of 3a.**

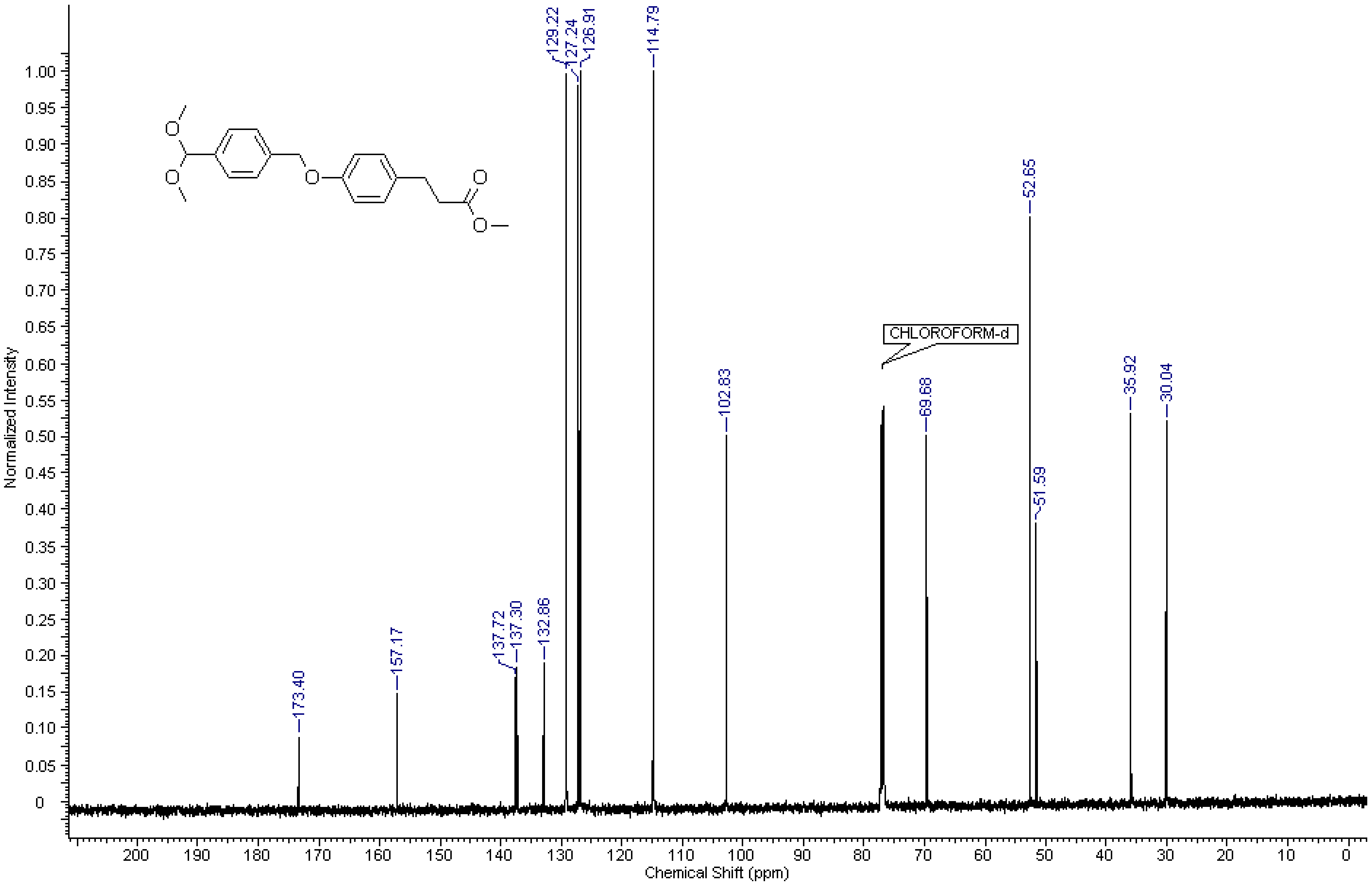

Figure S2. **^{13}C NMR spectrum of 3a.**

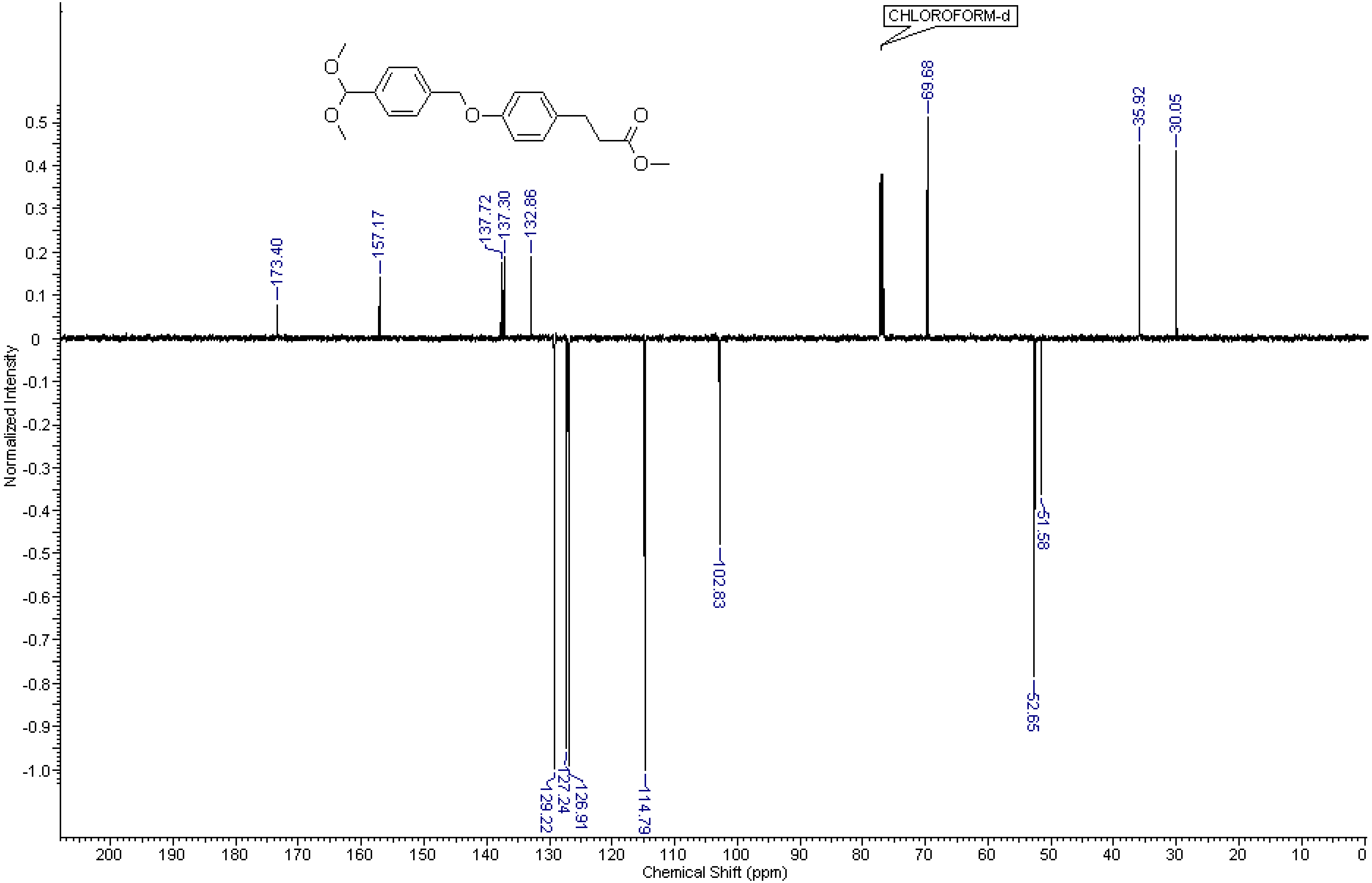

Figure S3. **^{13}C NMR spectrum of 3a (JMOD).**

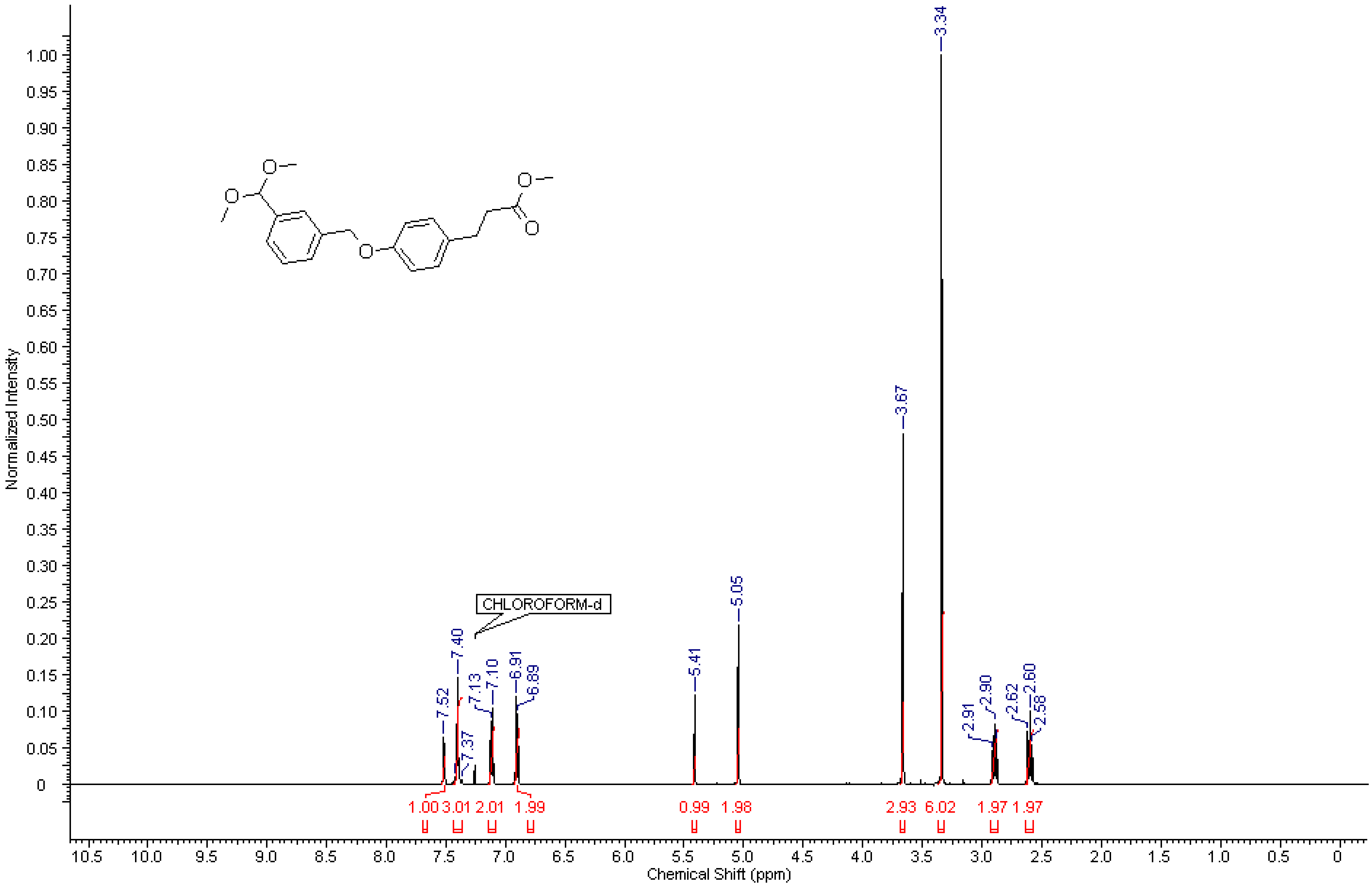

Figure S4. ¹**H NMR spectrum of 3b.**

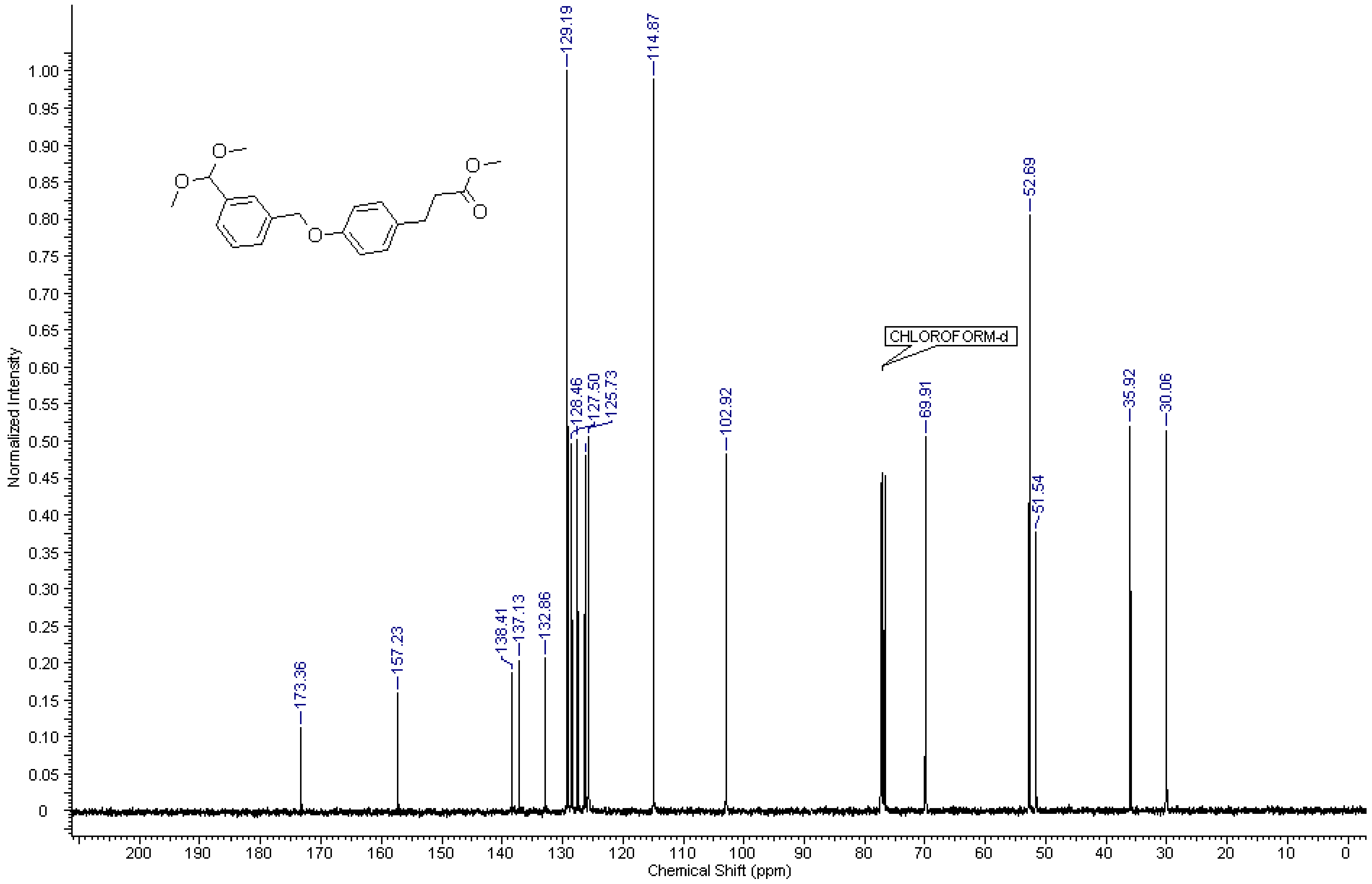

Figure S5. ^{13}**C NMR spectrum of 3b.**

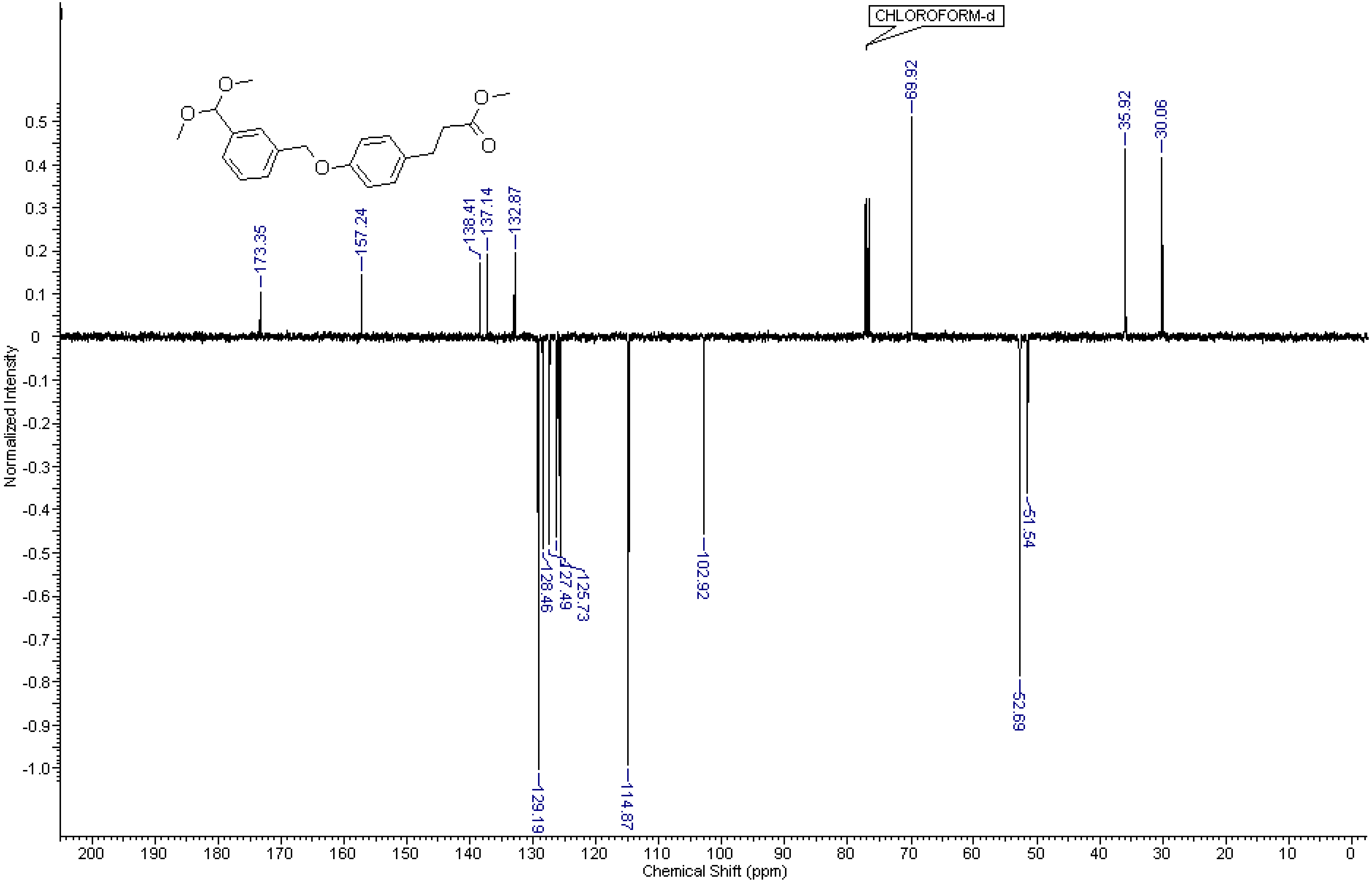

Figure S6. **^{13}C NMR spectrum of 3b. (JMOD)**

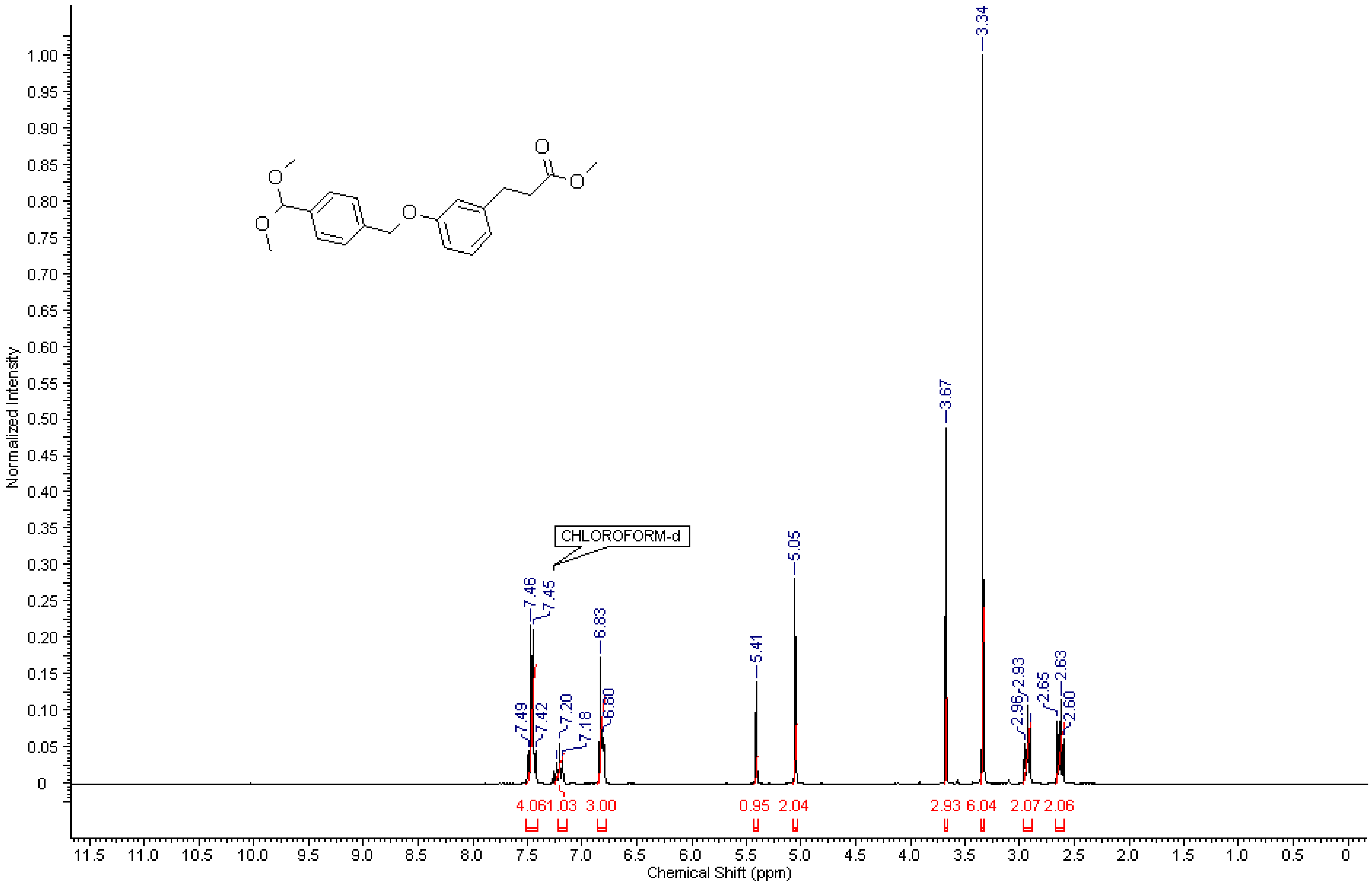

Figure S7. ¹H NMR spectrum of 3c.

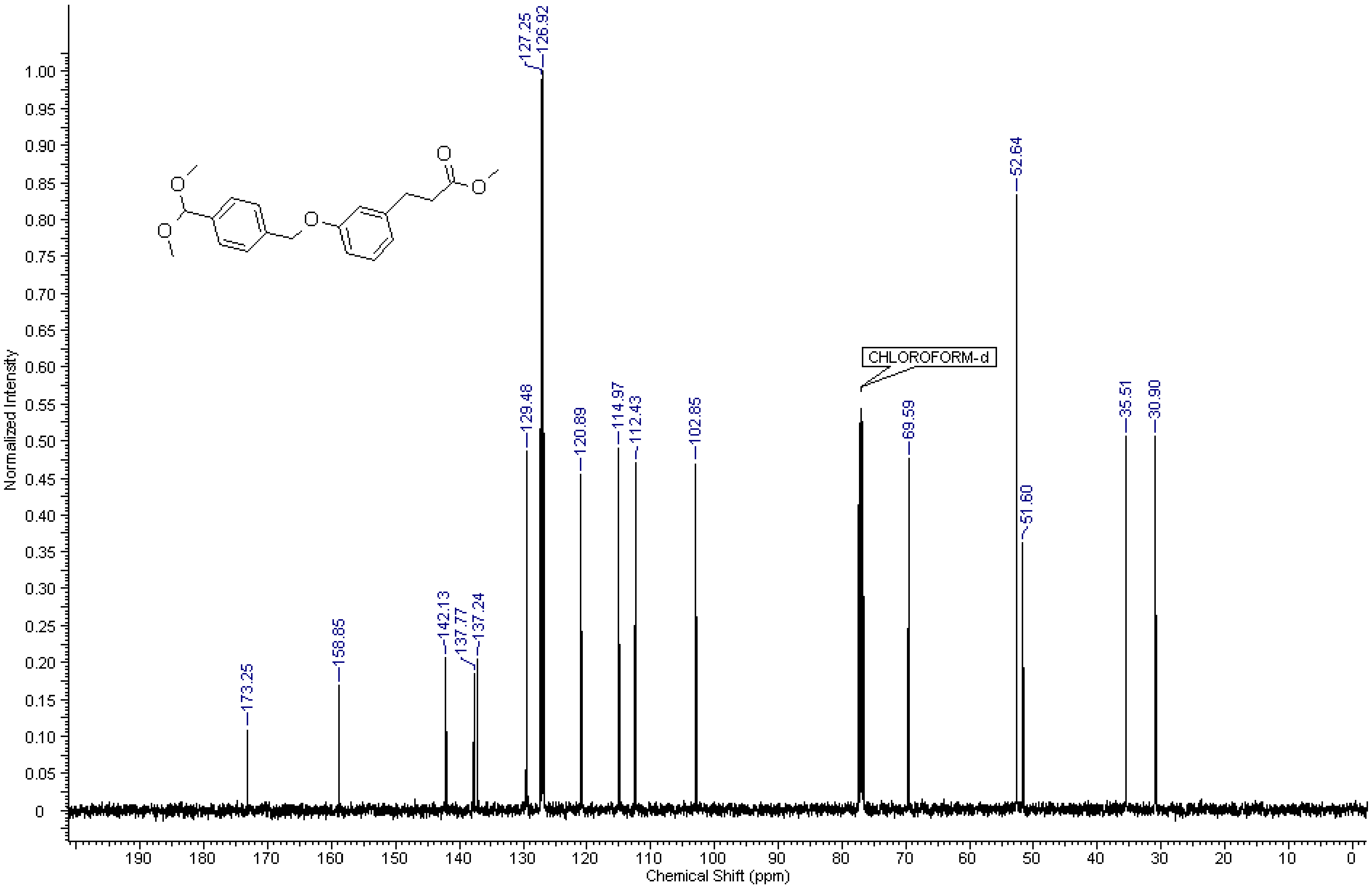

Figure S8. ^{13}C NMR spectrum of 3c.

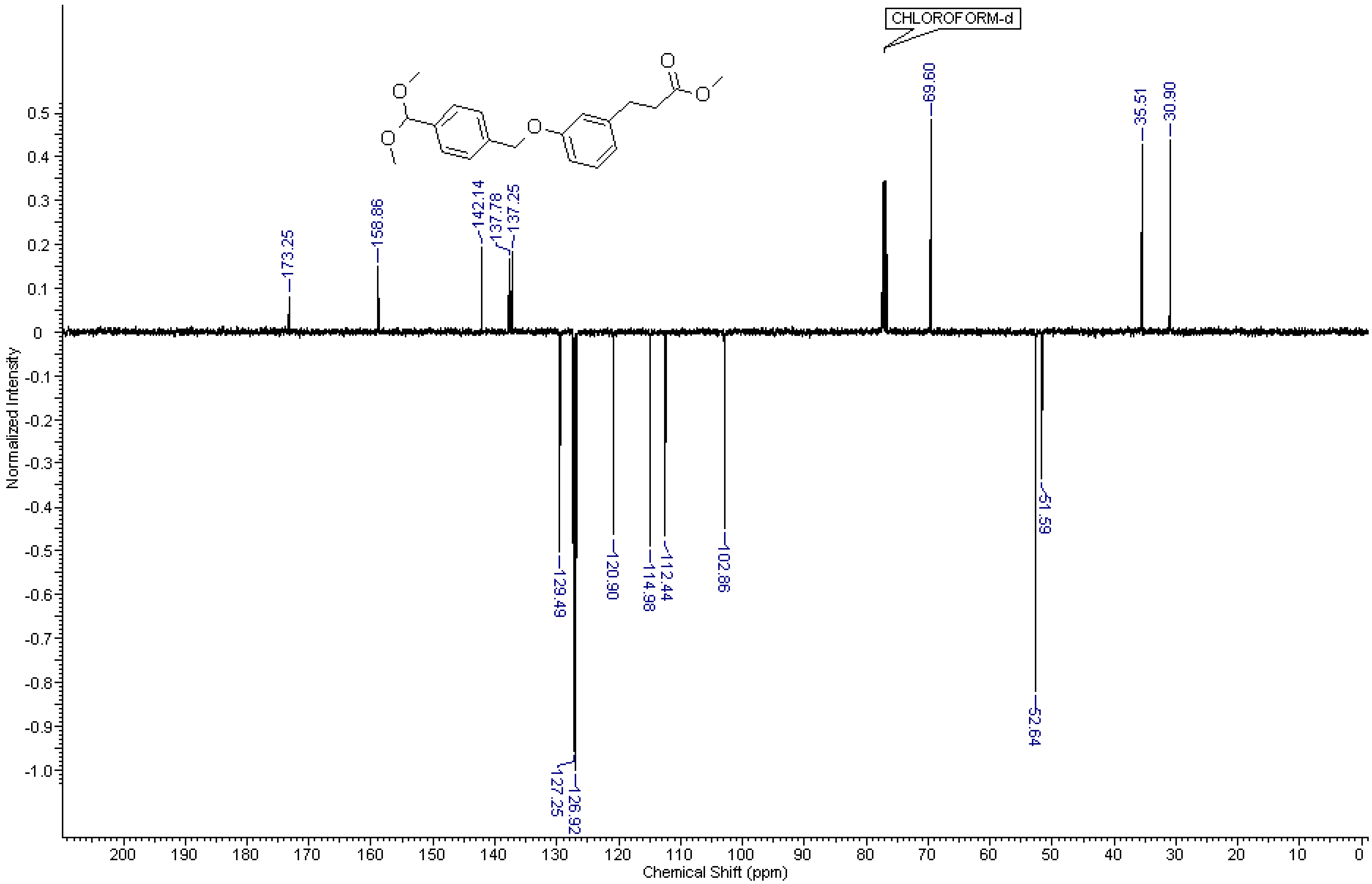

Figure S9. ^{13}C NMR spectrum of 3c (JMOD).

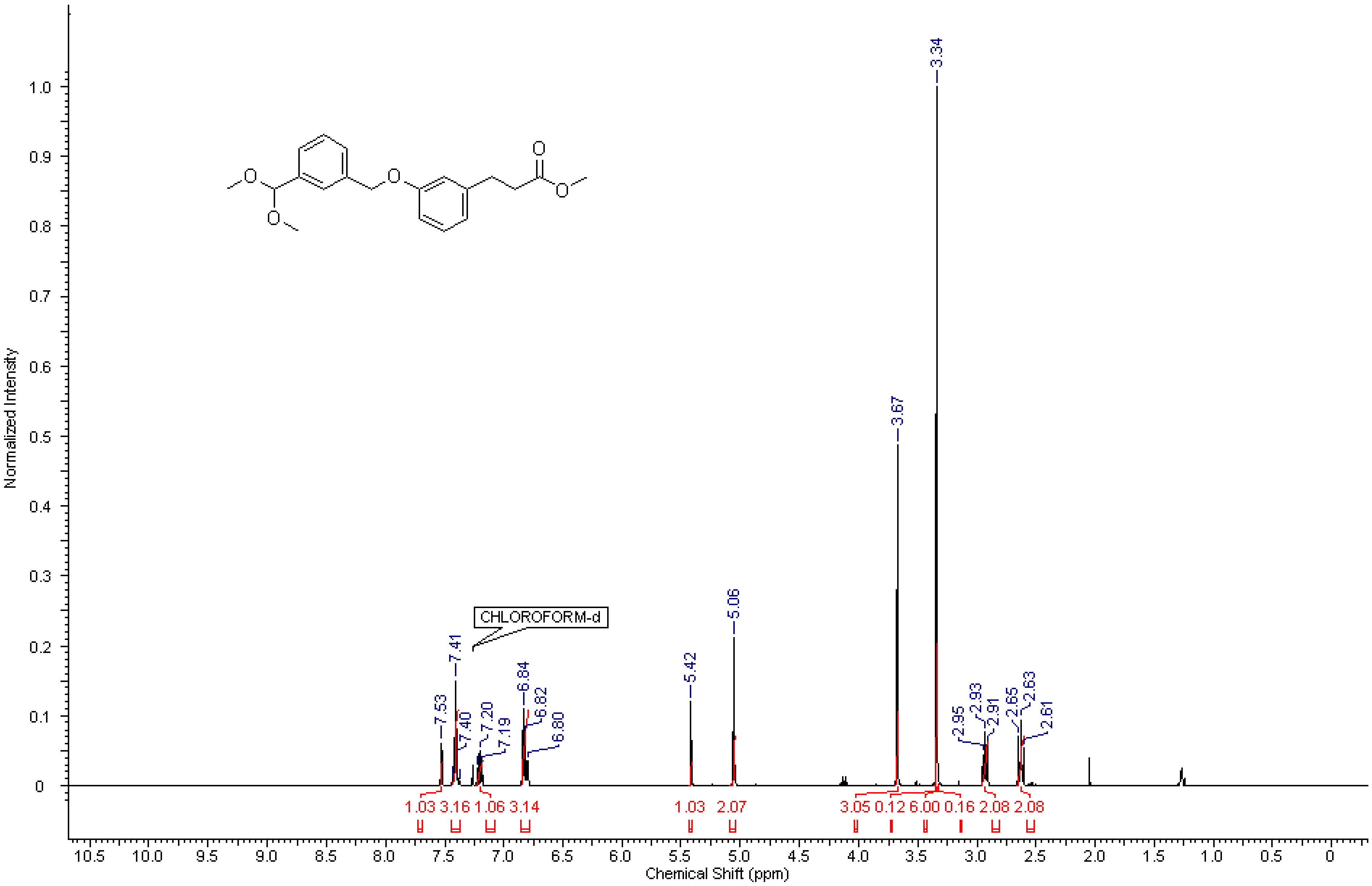

Figure S10. ¹H NMR spectrum of 3d.

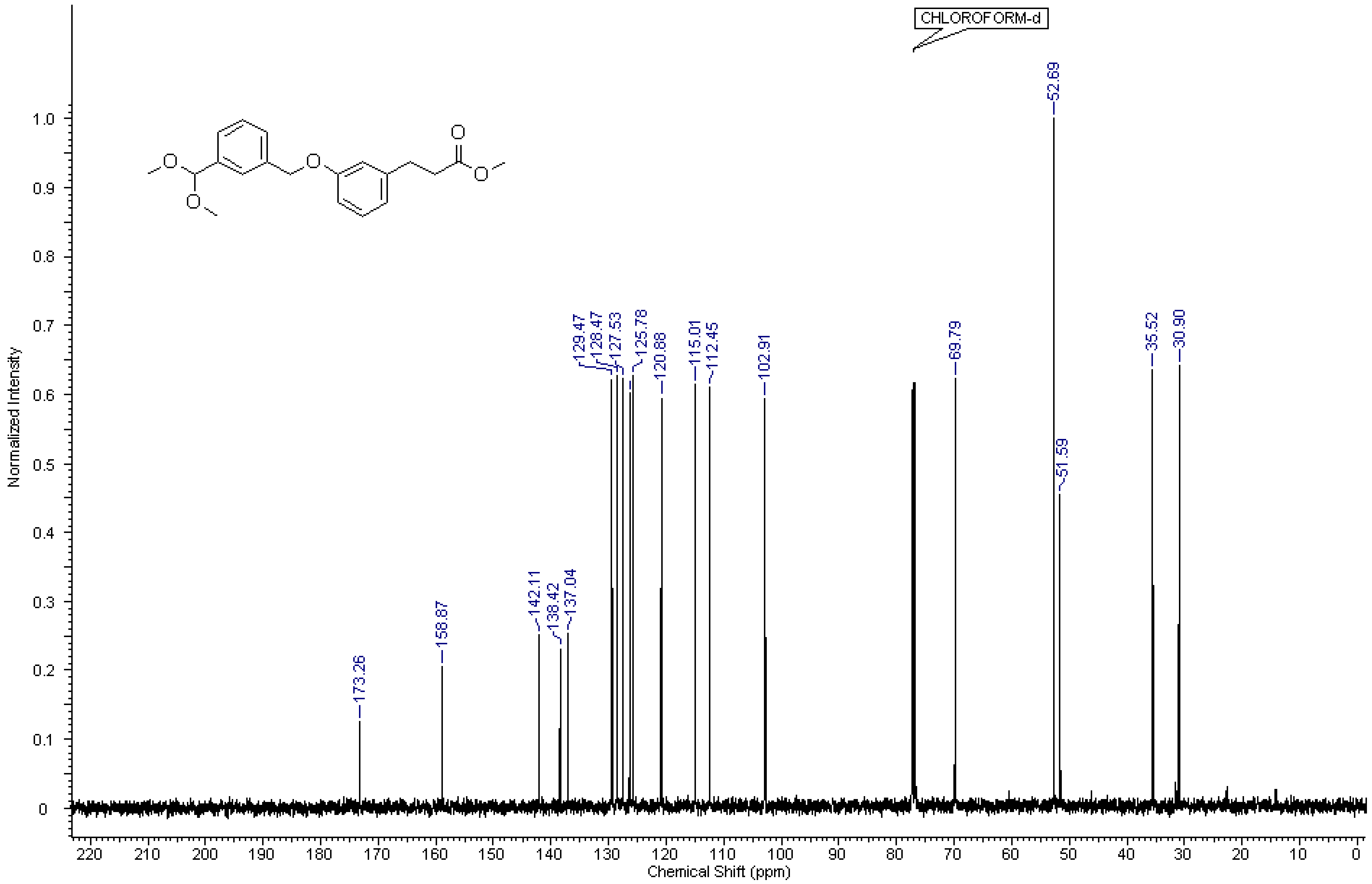

Figure S11. ^{13}C **NMR spectrum of 3d.**

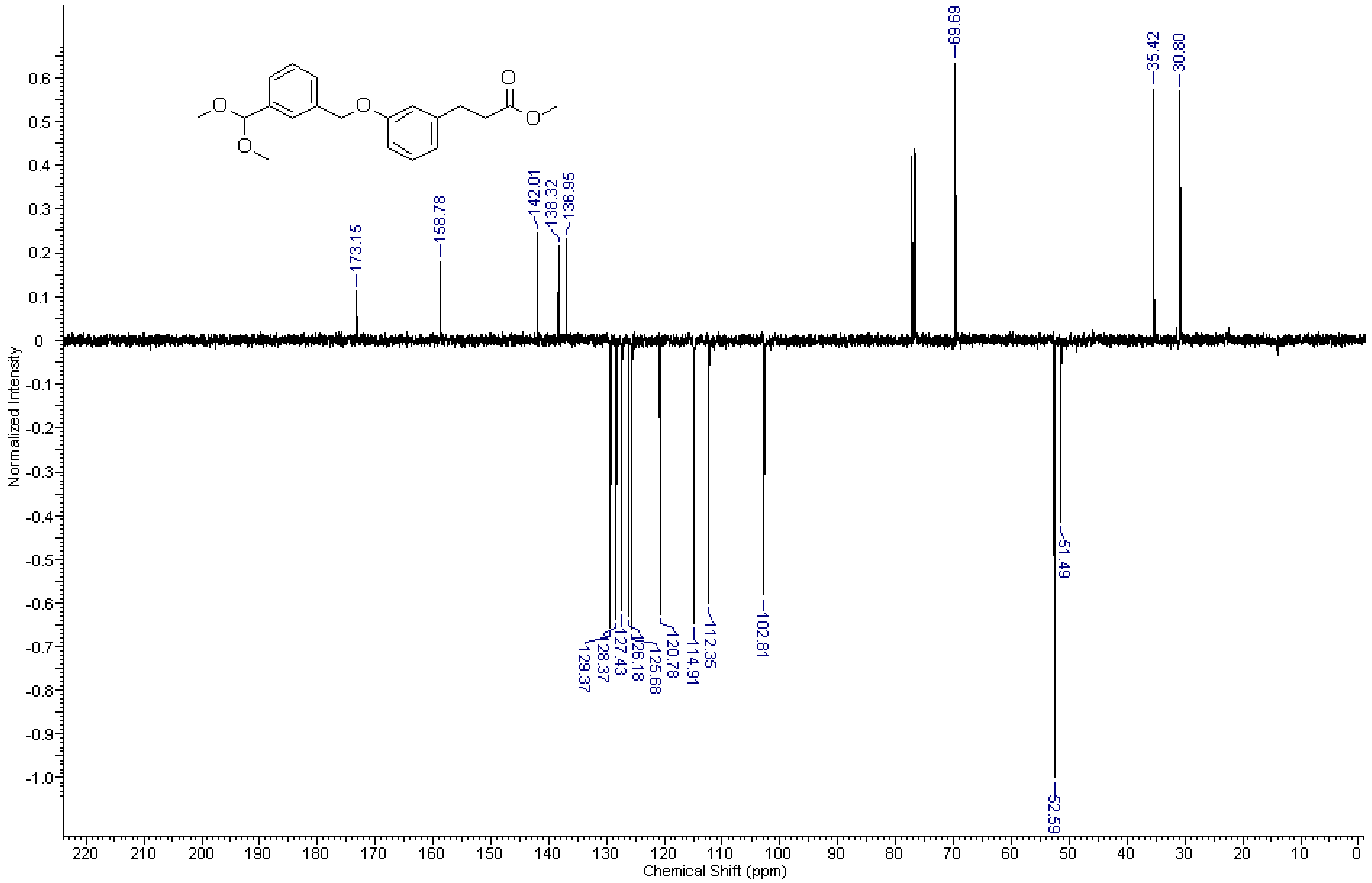

Figure S12. ^{13}C **NMR spectrum of 3d (JMOD).**

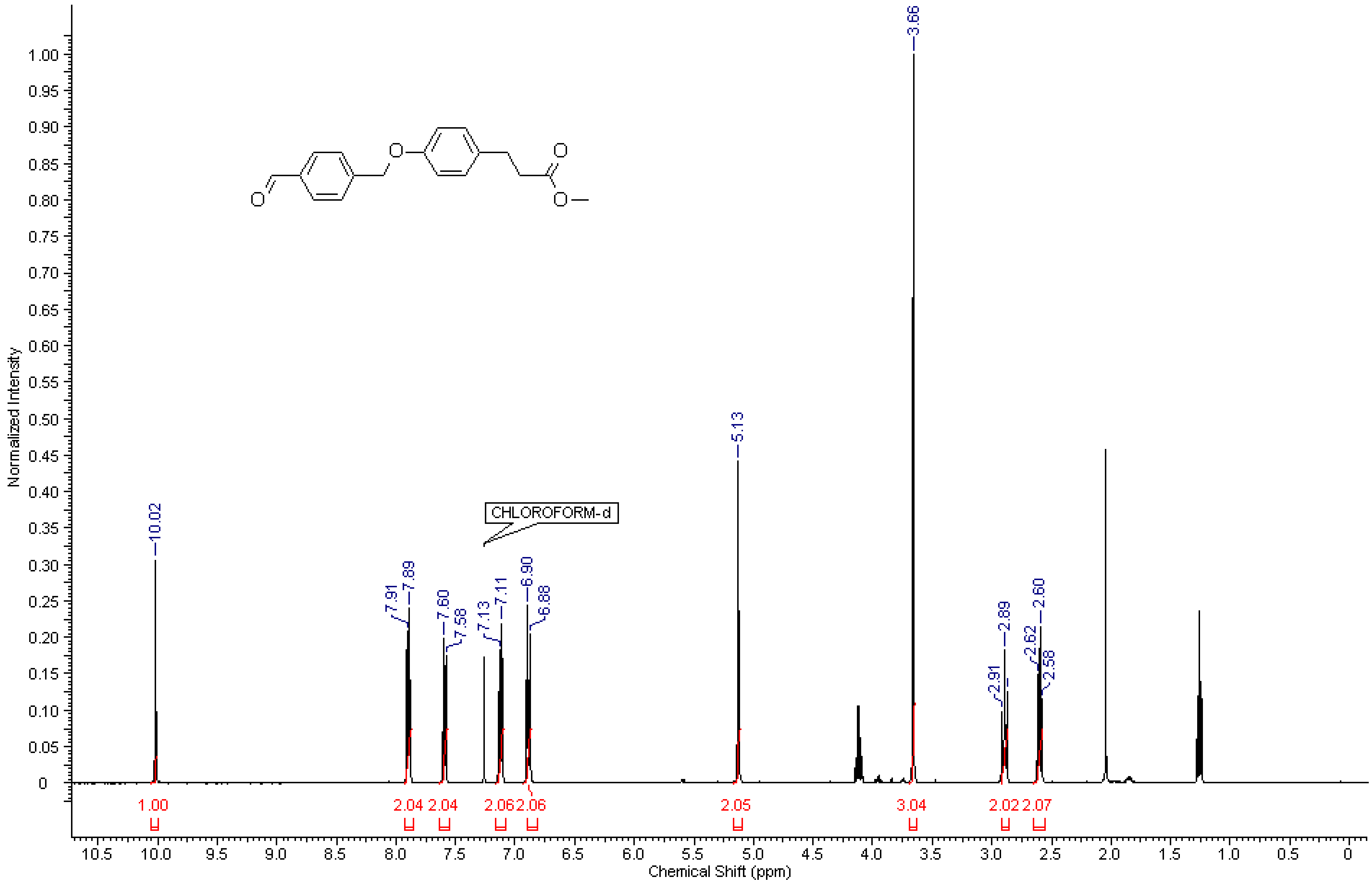

Figure S13. **^{1}H NMR spectrum of 4a.**

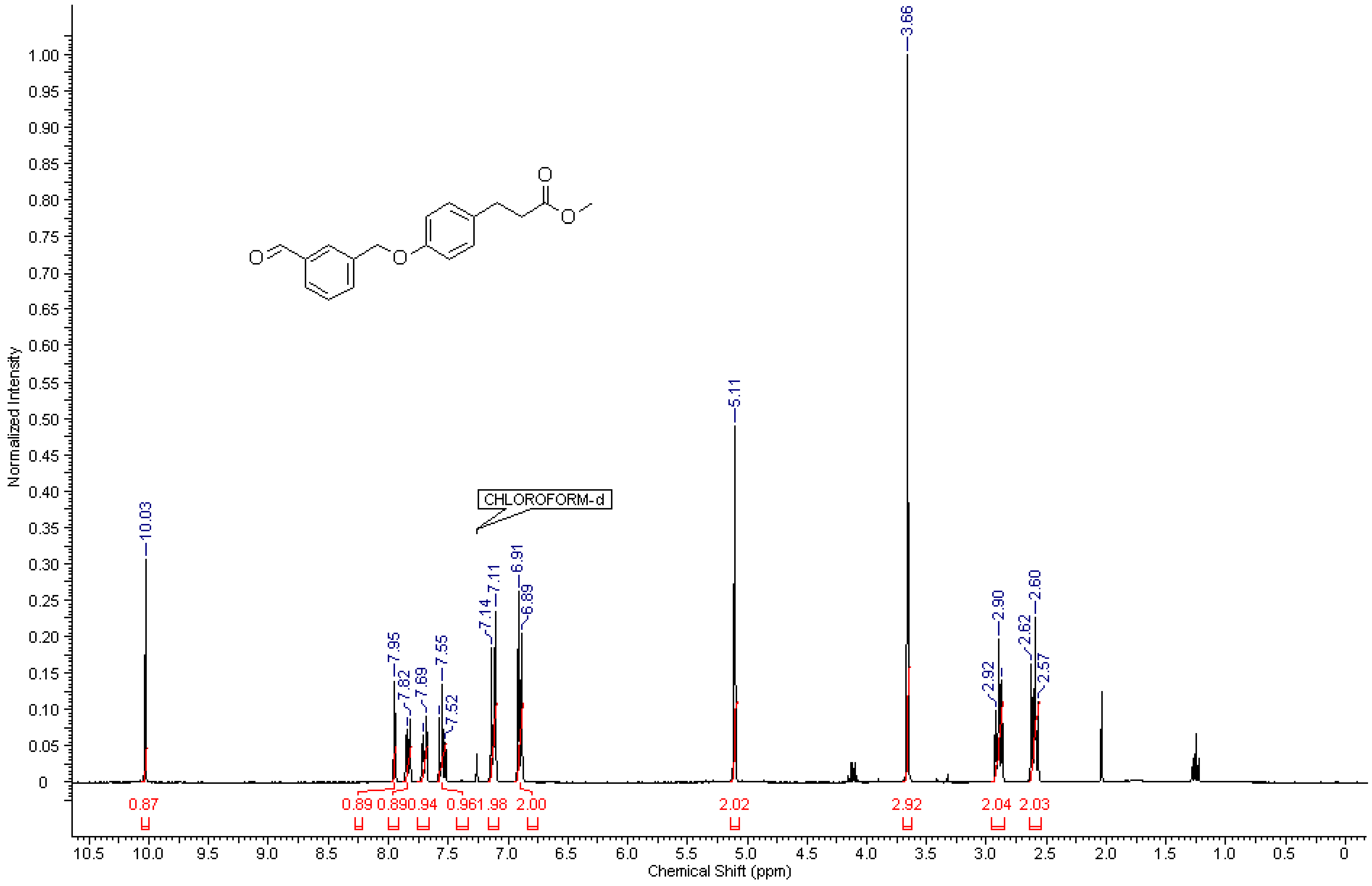

Figure S14. **^{1}H NMR spectrum of 4b.**

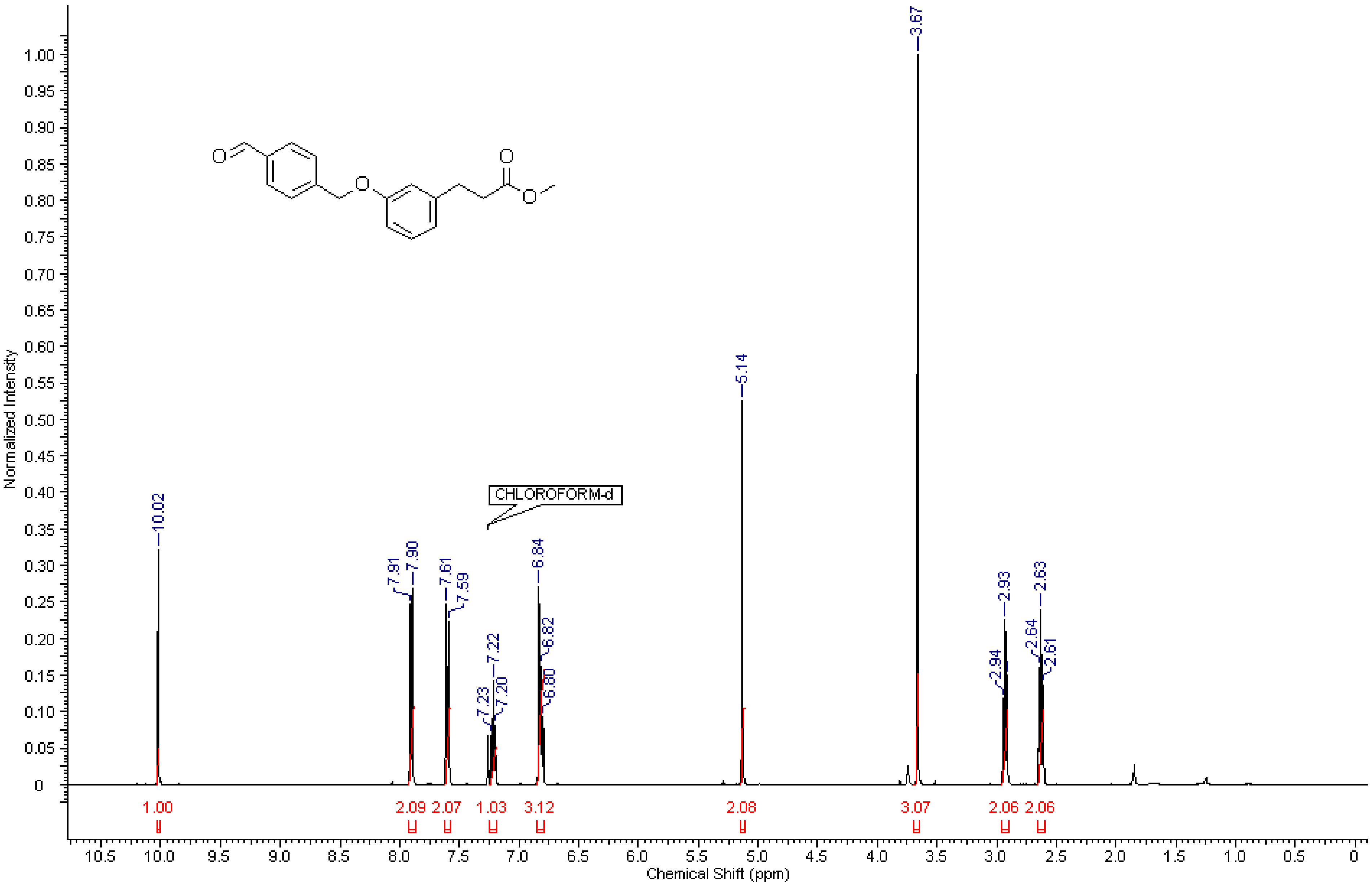

Figure S15. **^{1}H NMR spectrum of 4c.**

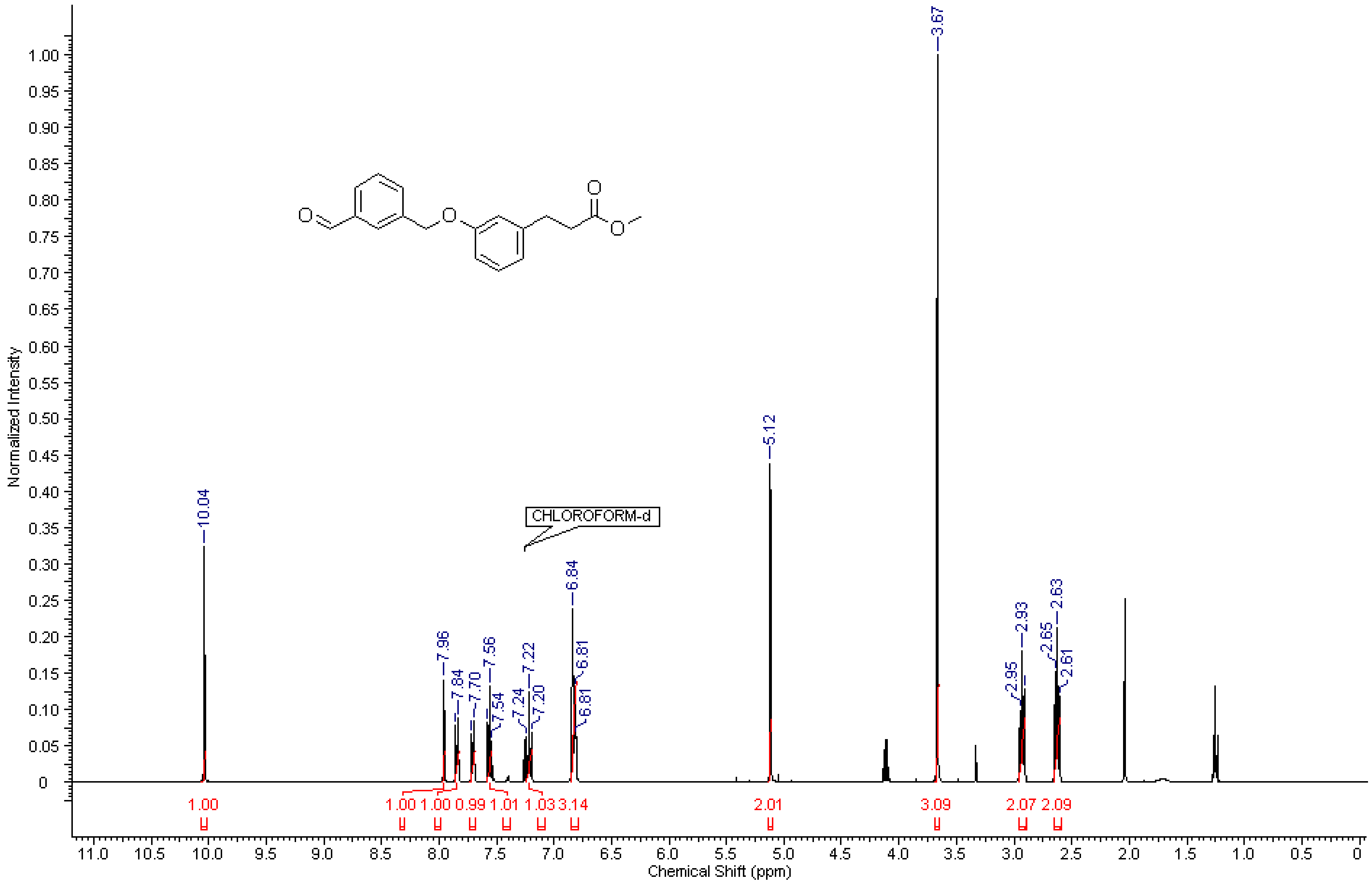

Figure S16. **¹H NMR spectrum of 4d.**

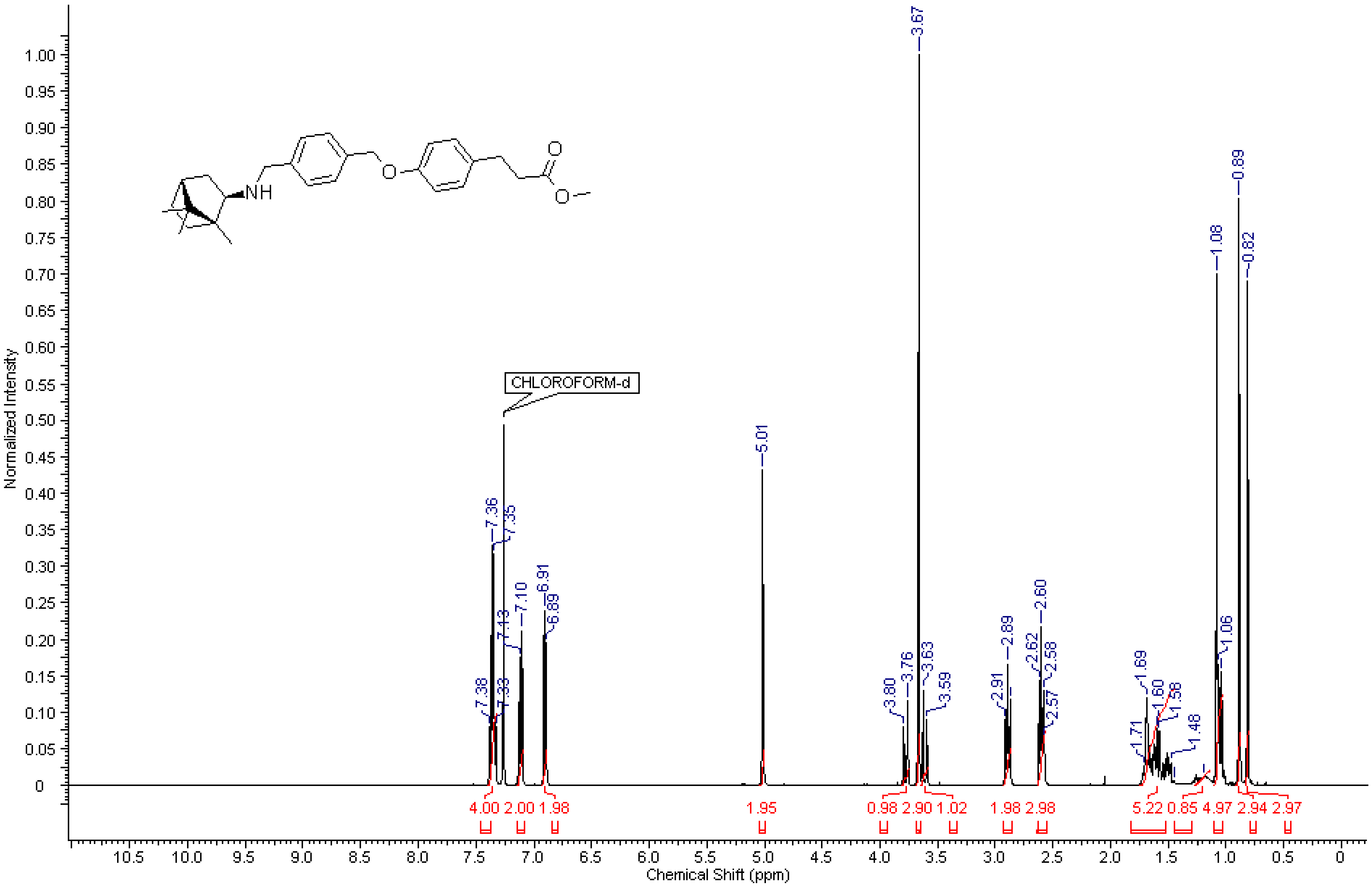

Figure S17. **^{1}H NMR spectrum of 5a.**

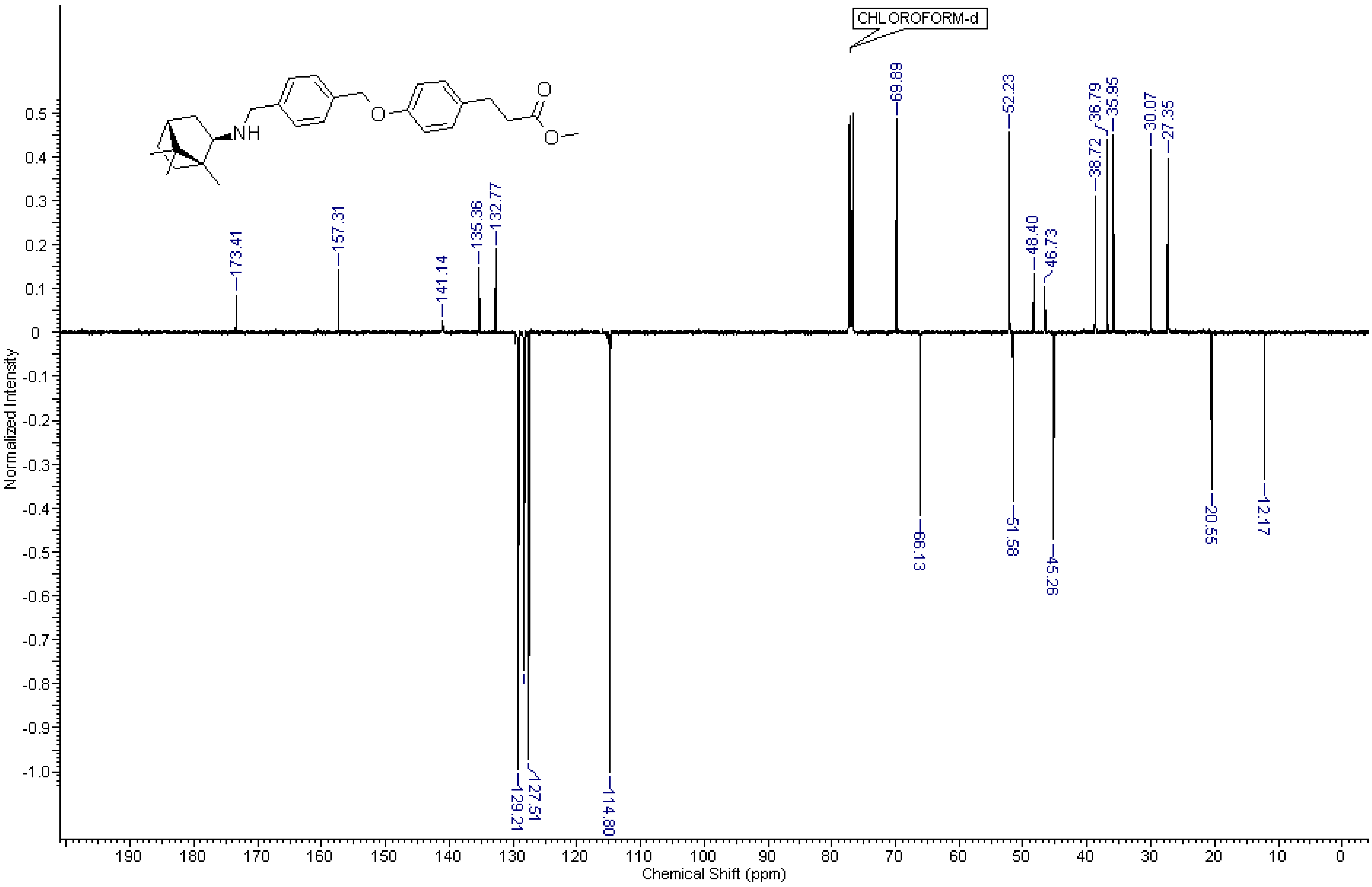

Figure S18. ^{13}**C NMR spectrum of 5a (JMOD).**

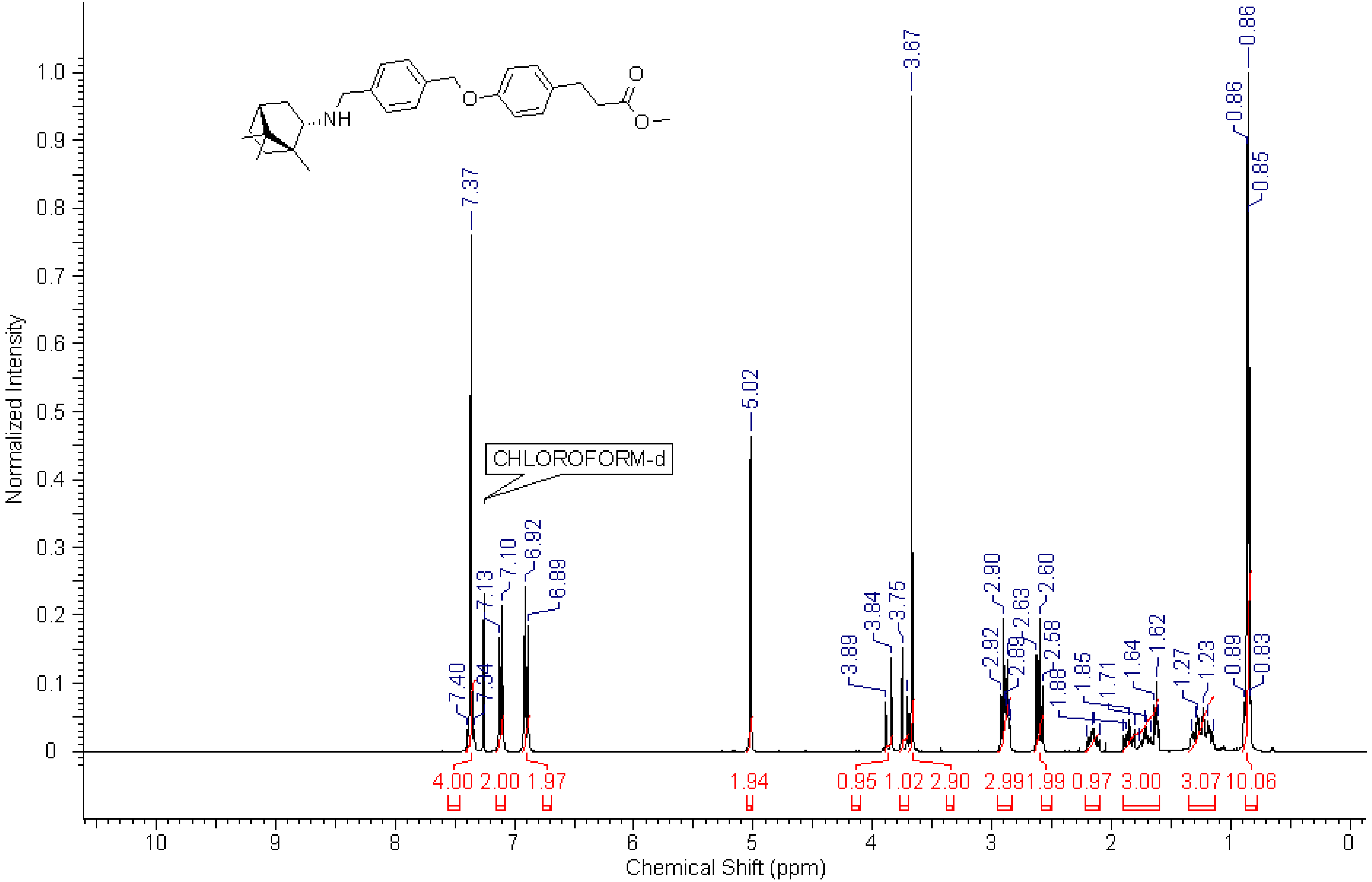

Figure S19. ^{1}H NMR spectrum of **5b**.

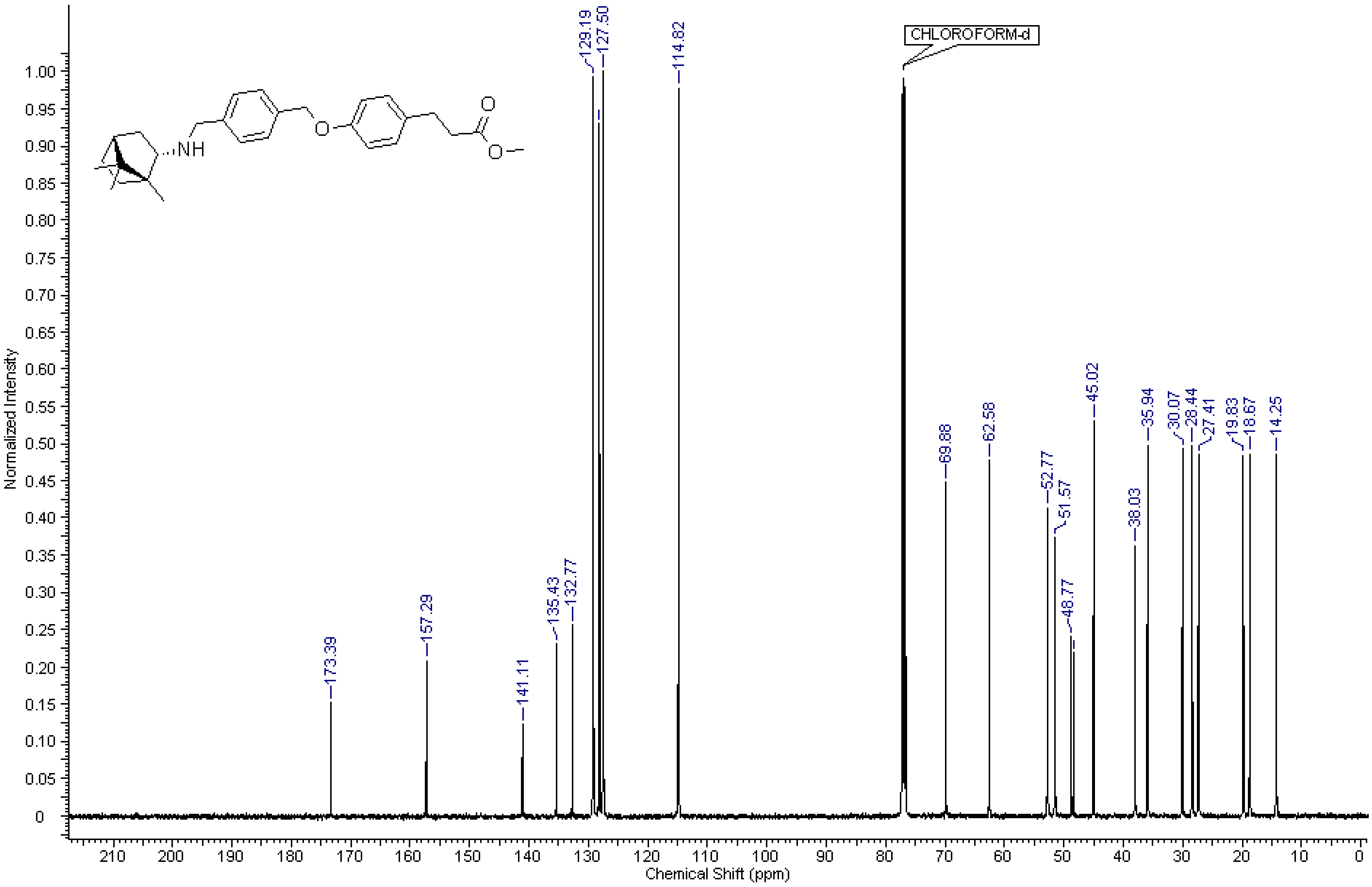

Figure S20. ^{13}C NMR spectrum of 5b.

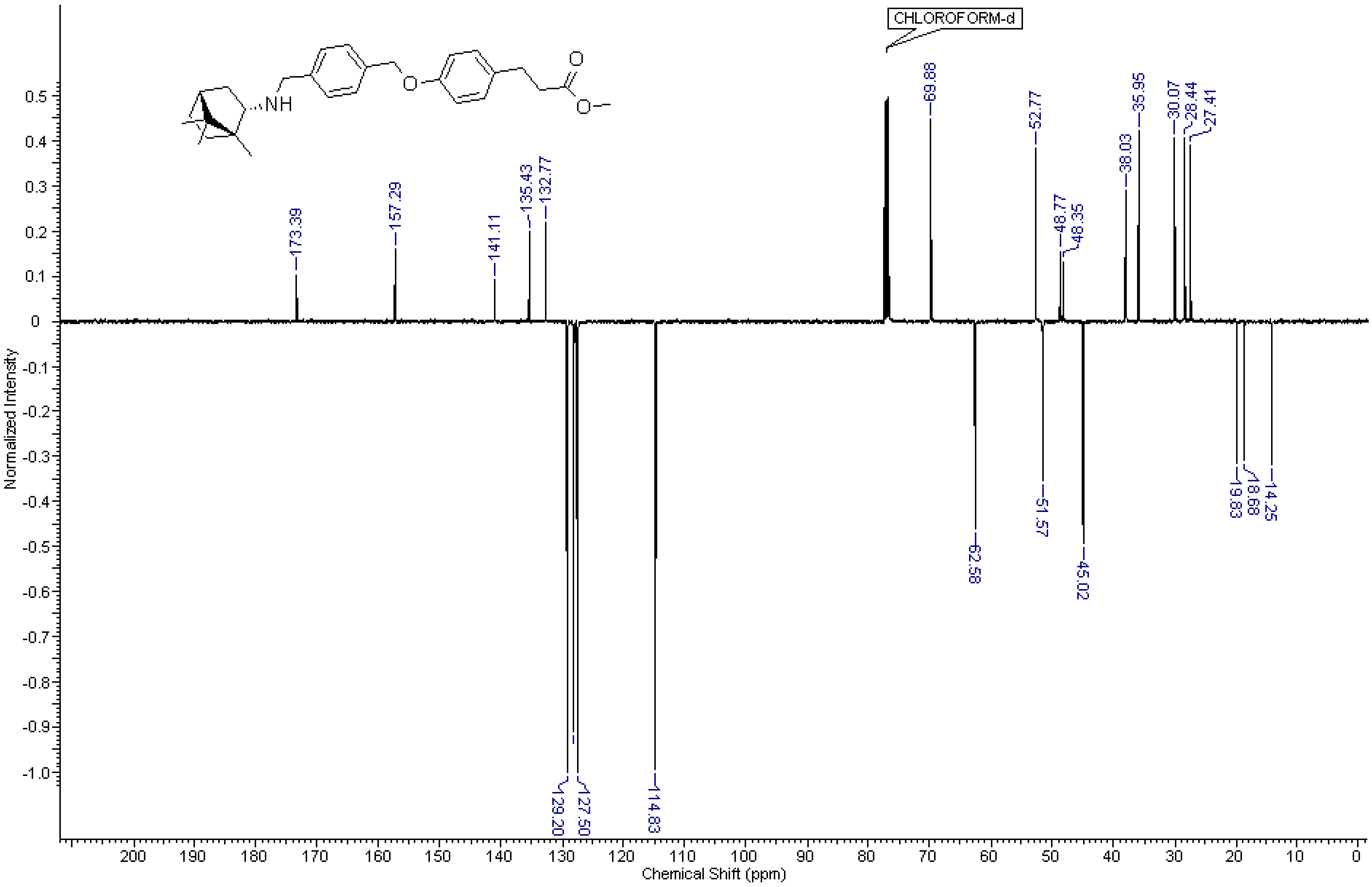

Figure S21. ¹³C NMR spectrum of 5b (JMOD).

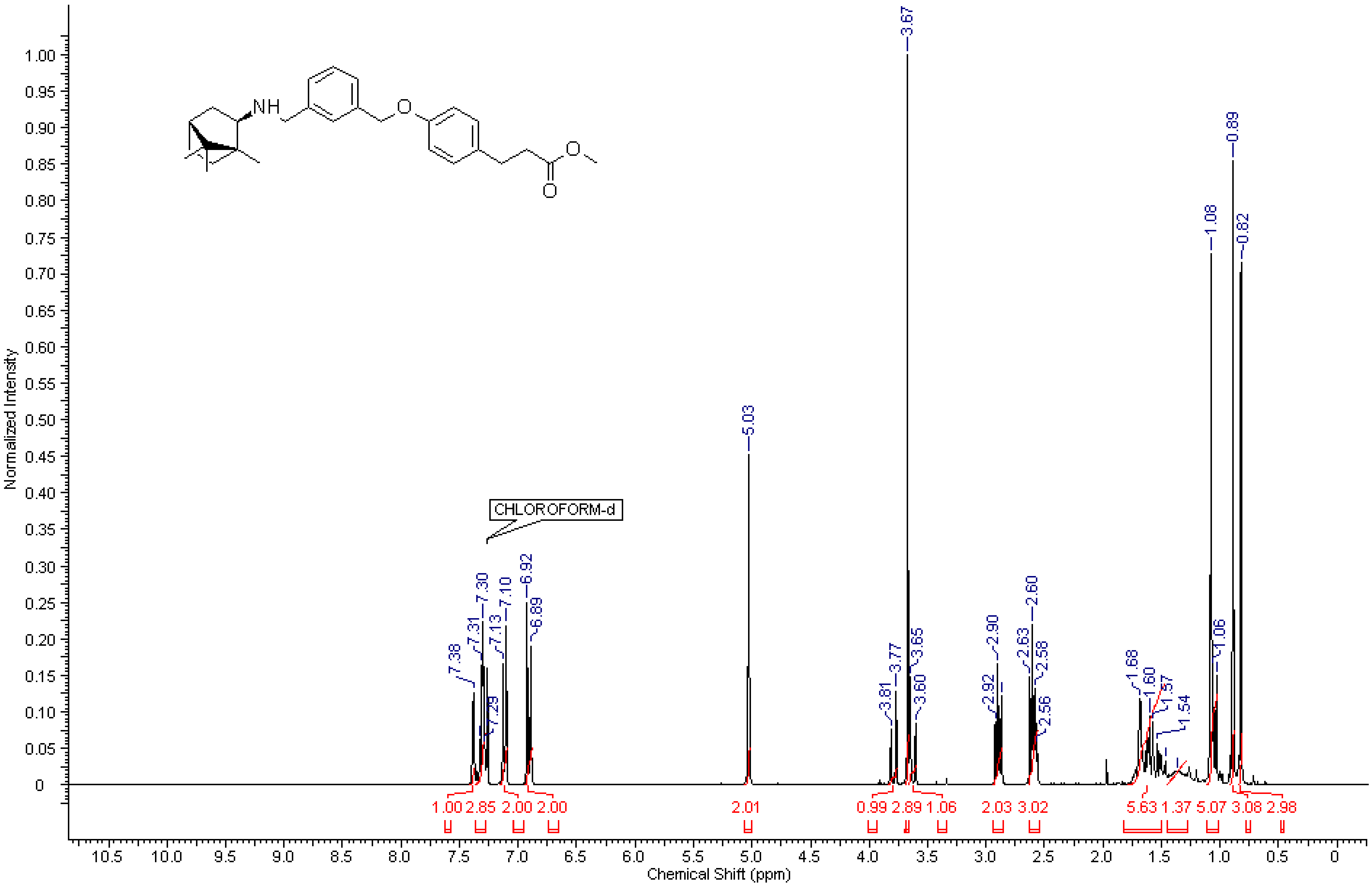

Figure S22. ^{1}**H NMR spectrum of 5c.**

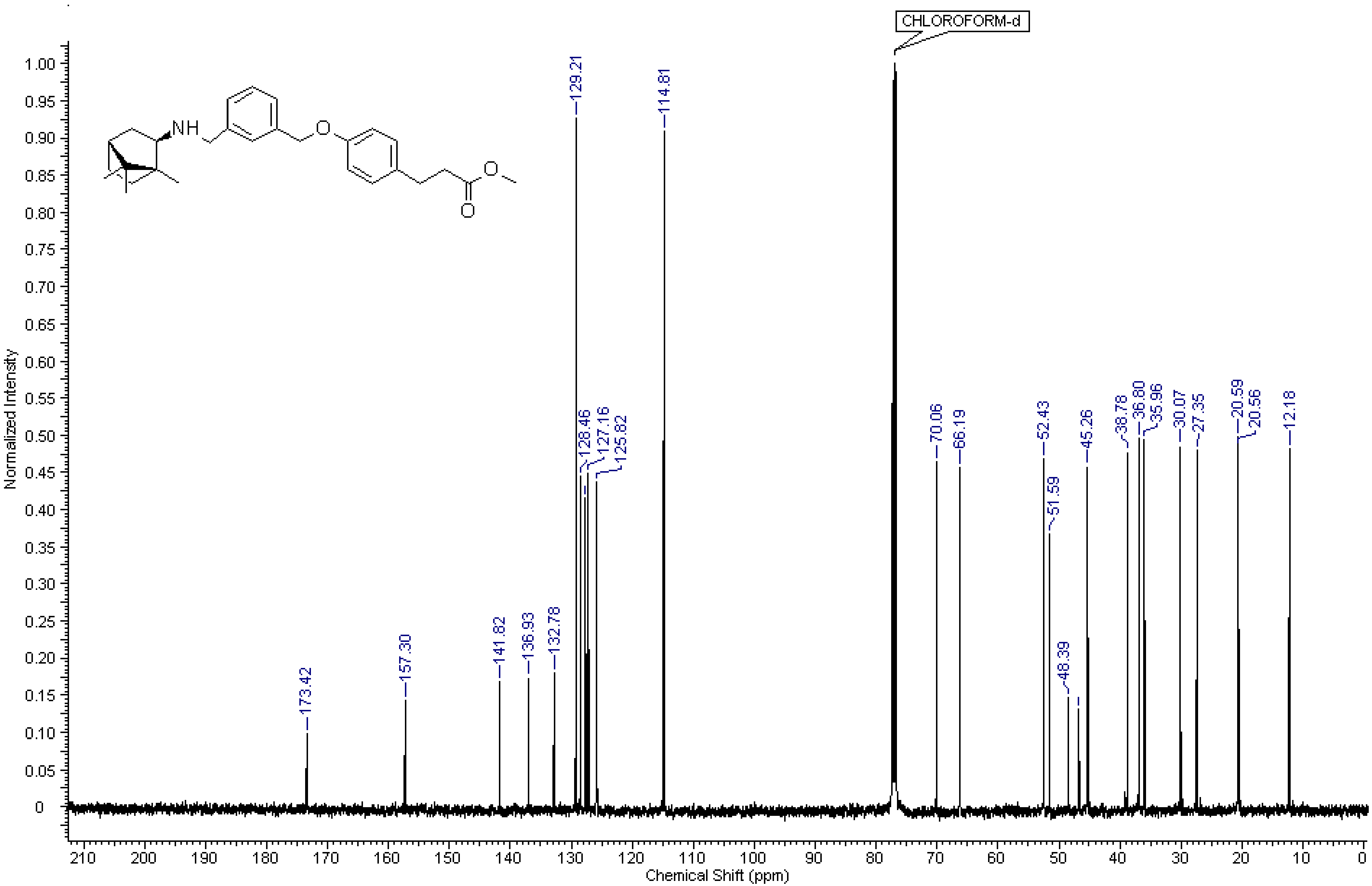

Figure S23. **^{13}C NMR spectrum of 5c.**

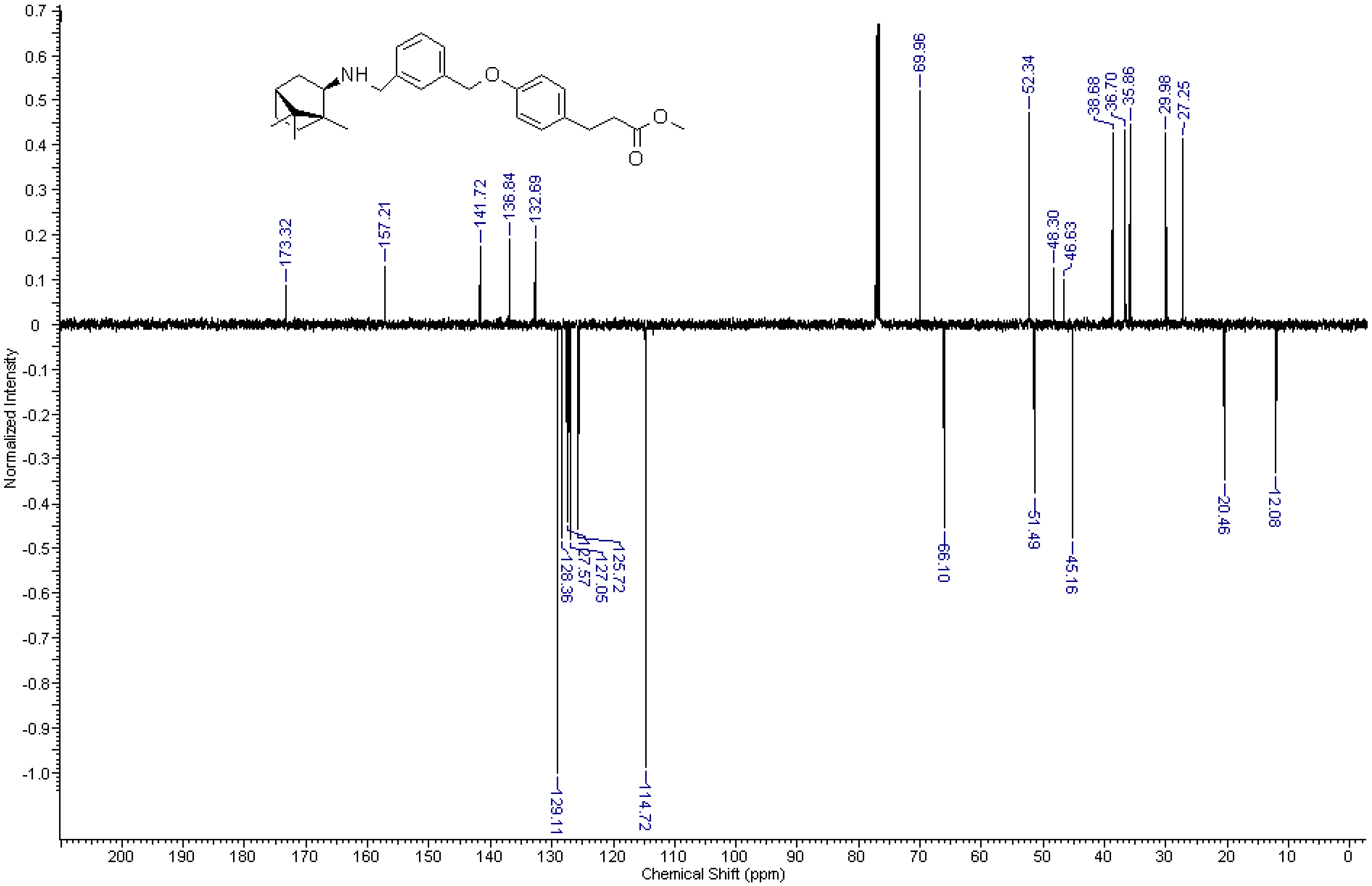

Figure S24. **^{13}C NMR spectrum of 5c (JMOD).**

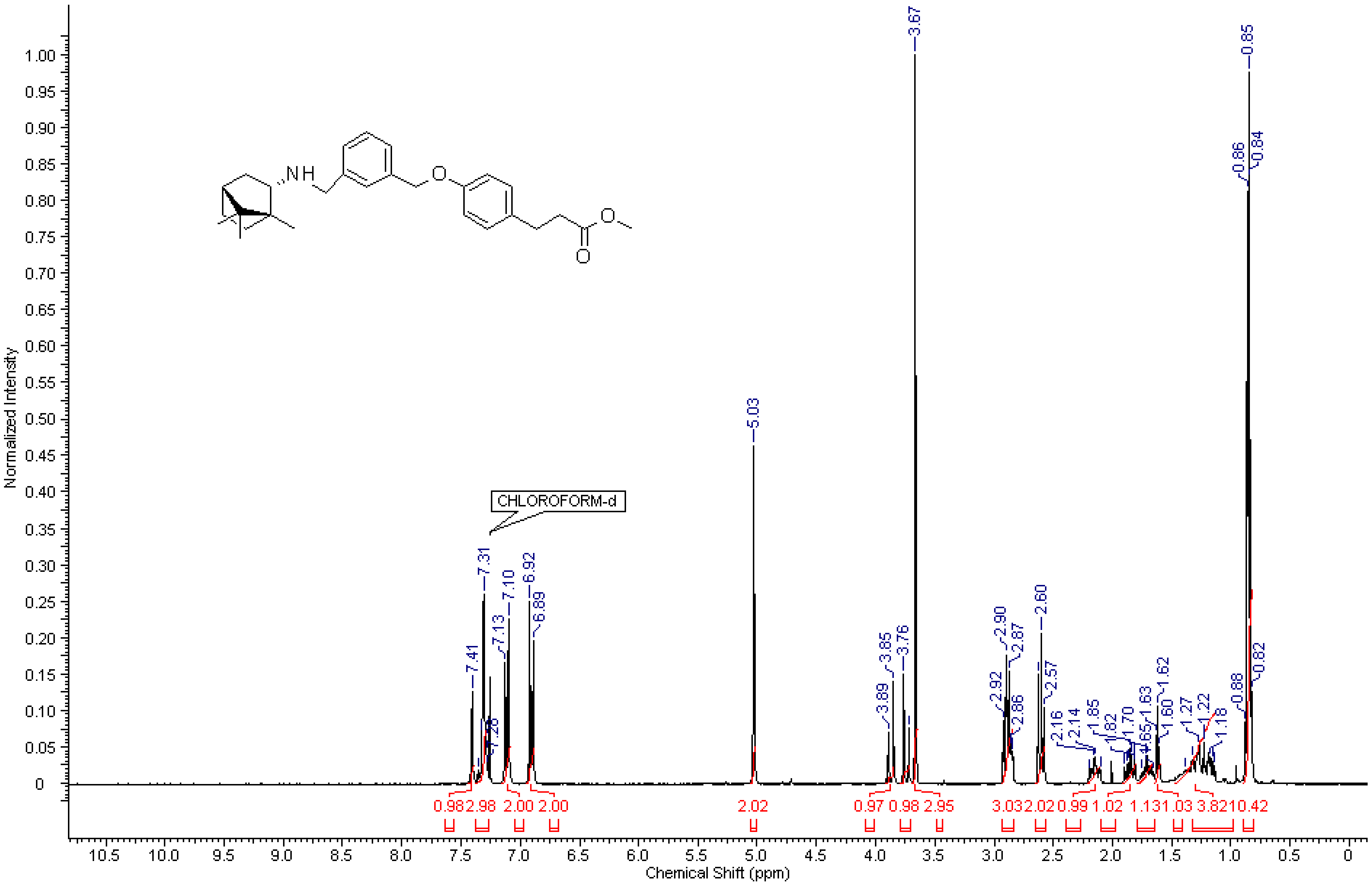

Figure S25. ^{1}**H NMR spectrum of 5d.**

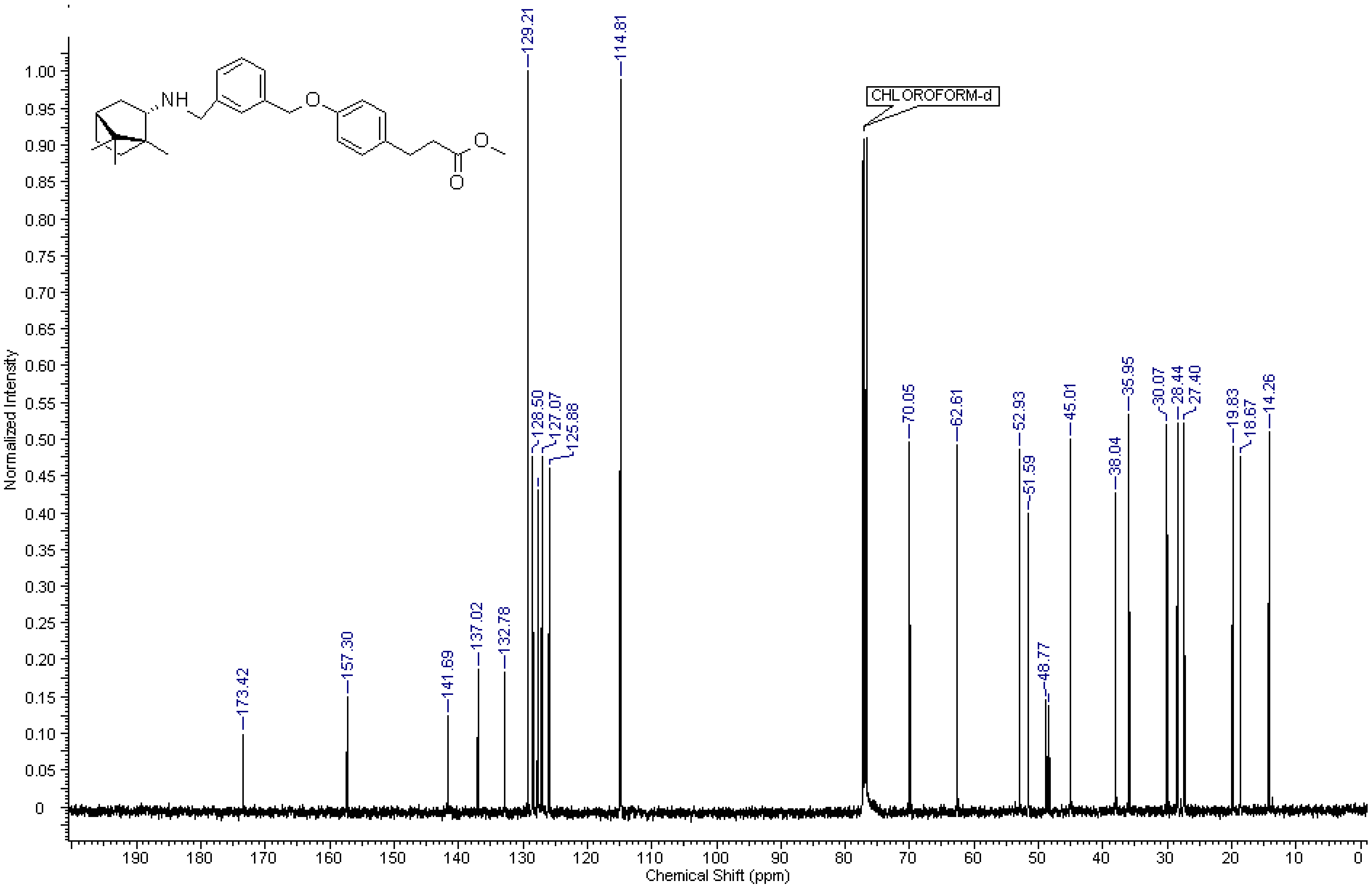

Figure S26. **^{13}C NMR spectrum of 5d.**

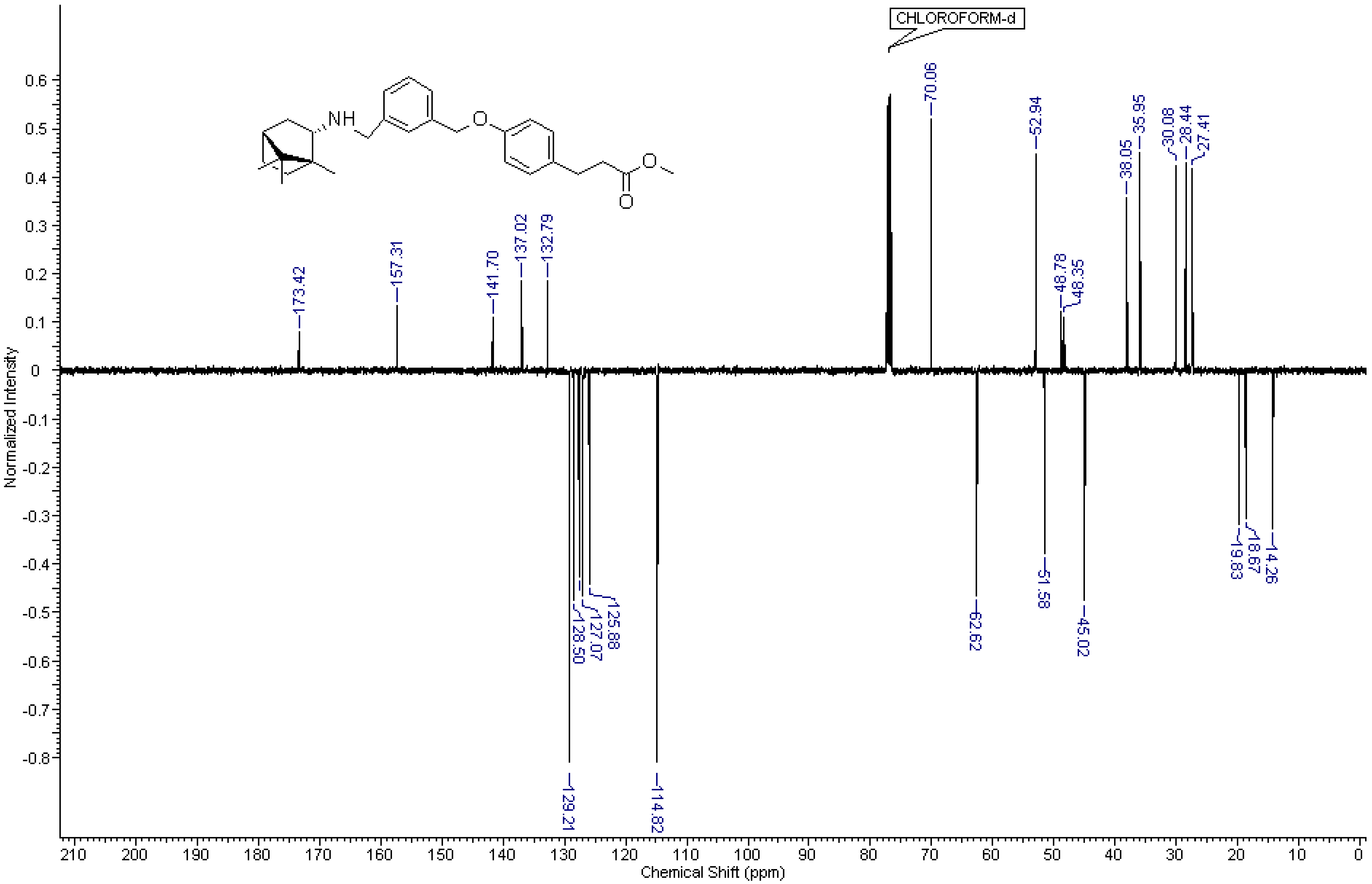

Figure S27. **^{13}C NMR spectrum of 5d (JMOD).**

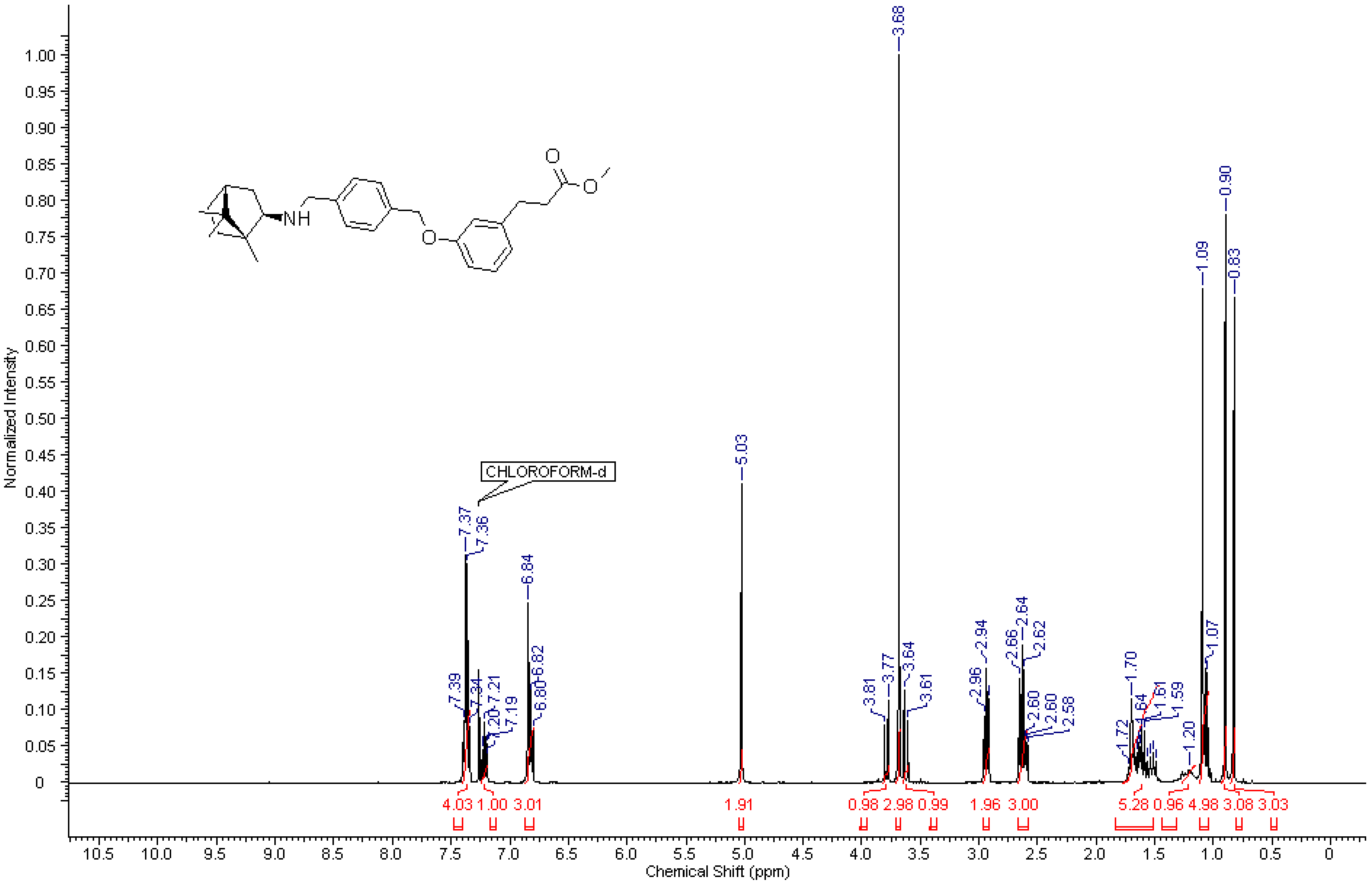

Figure S28. [1]**H NMR spectrum of 5e.**

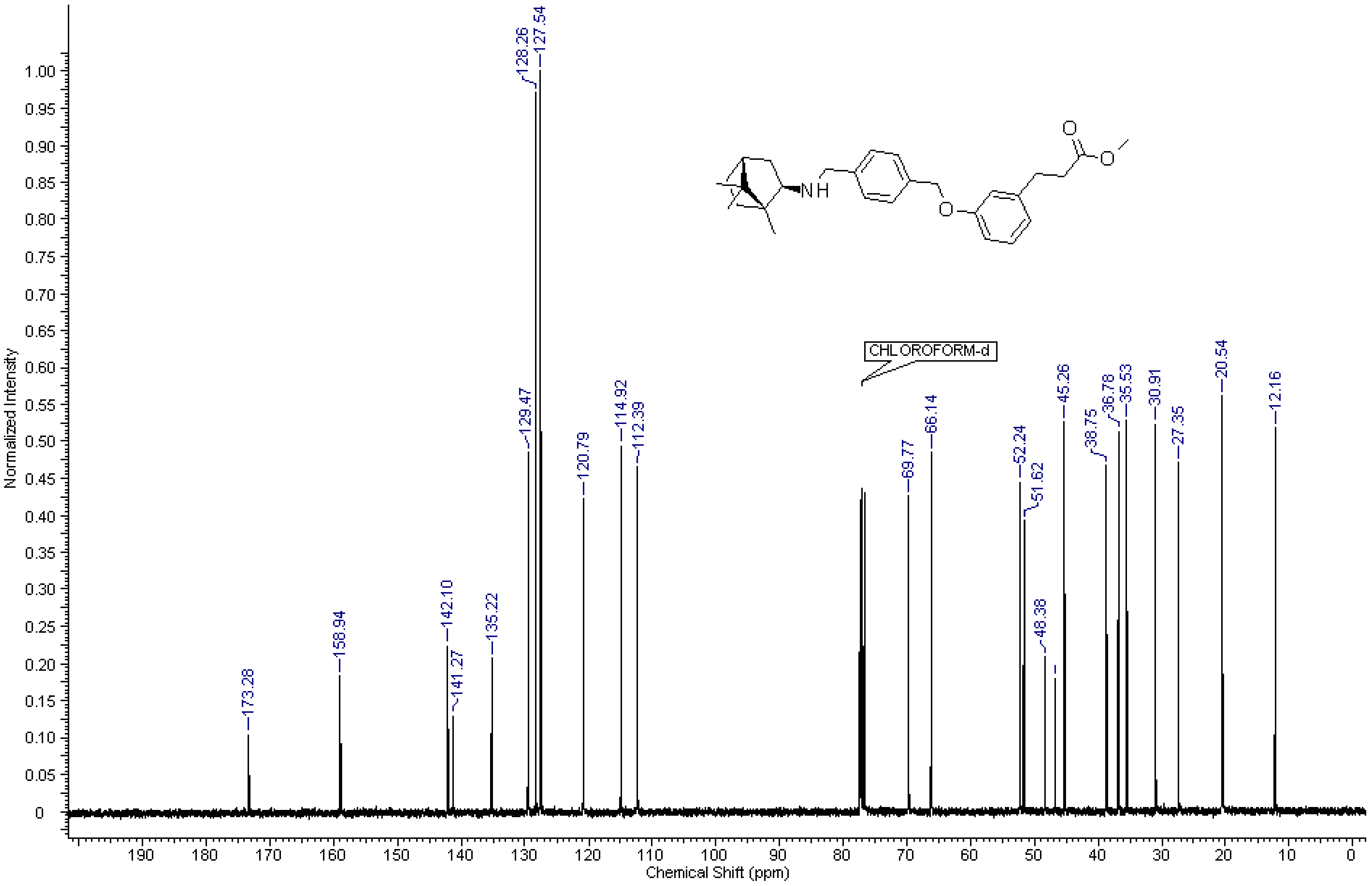

Figure S29. **^{13}C NMR spectrum of 5e.**

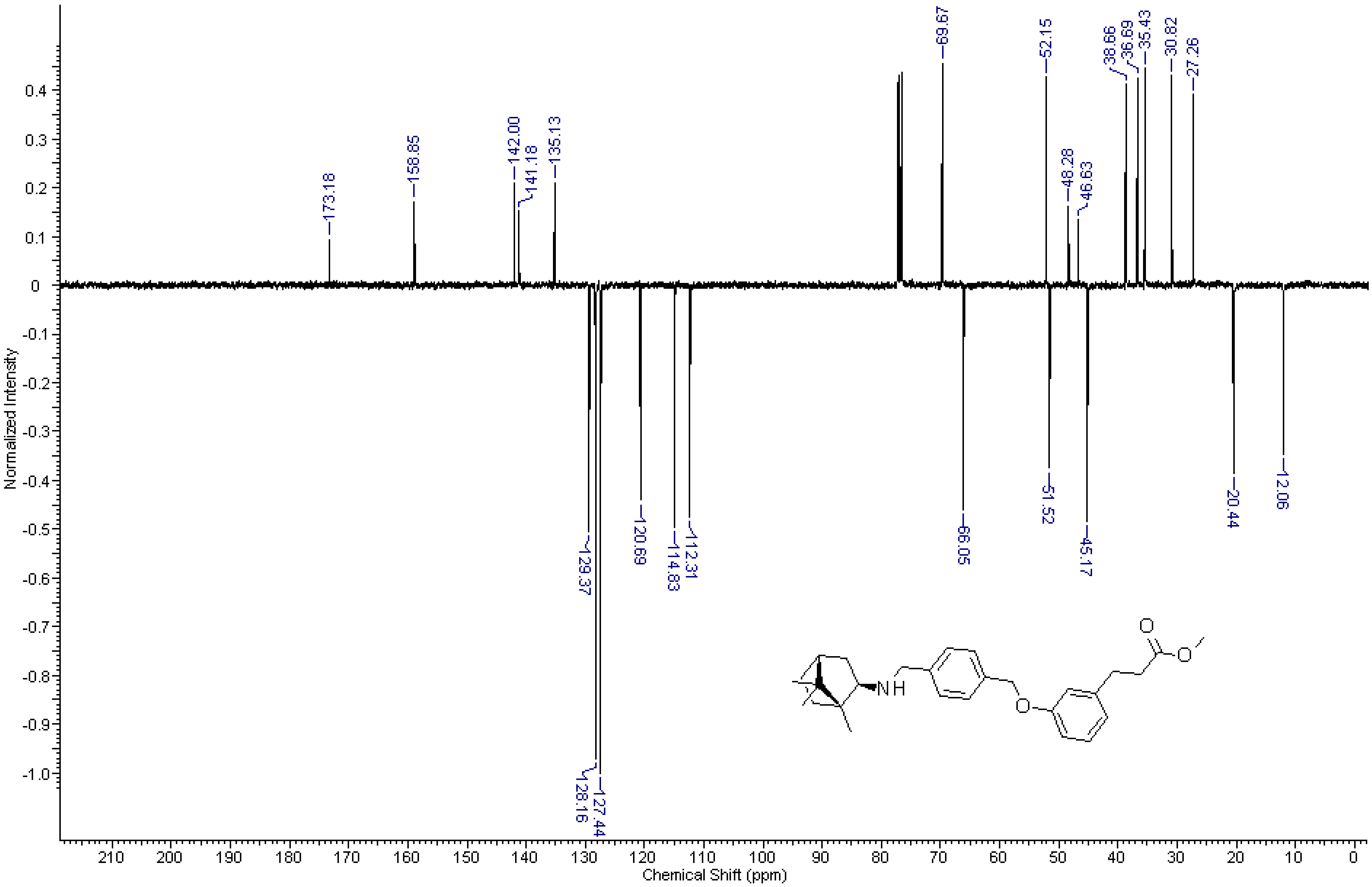

Figure S30. **^{13}C NMR spectrum of 5e (JMOD).**

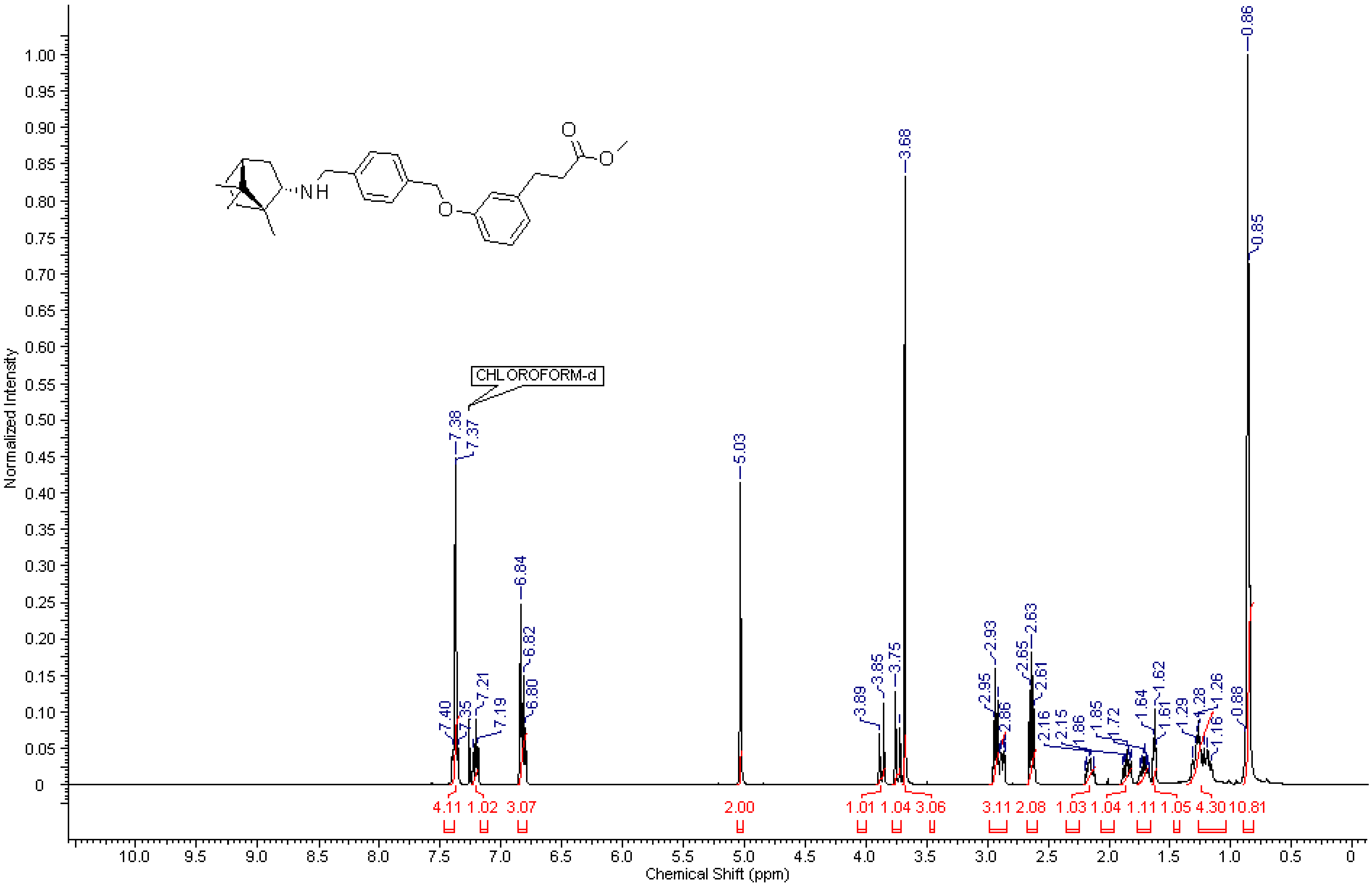

Figure S31. ^{1}H NMR spectrum of 5f.

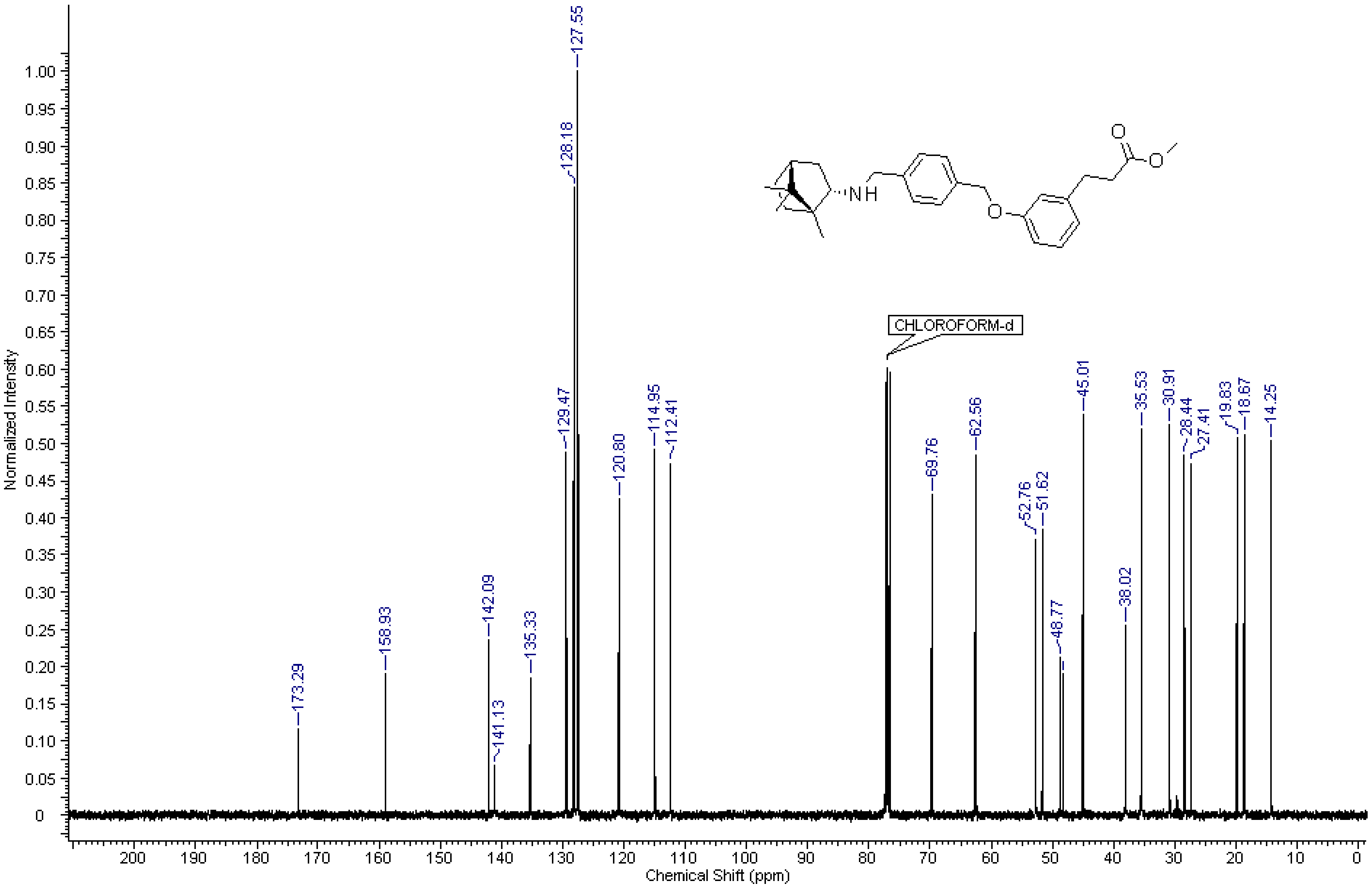

Figure S32. ^{13}**C NMR spectrum of 5f.**

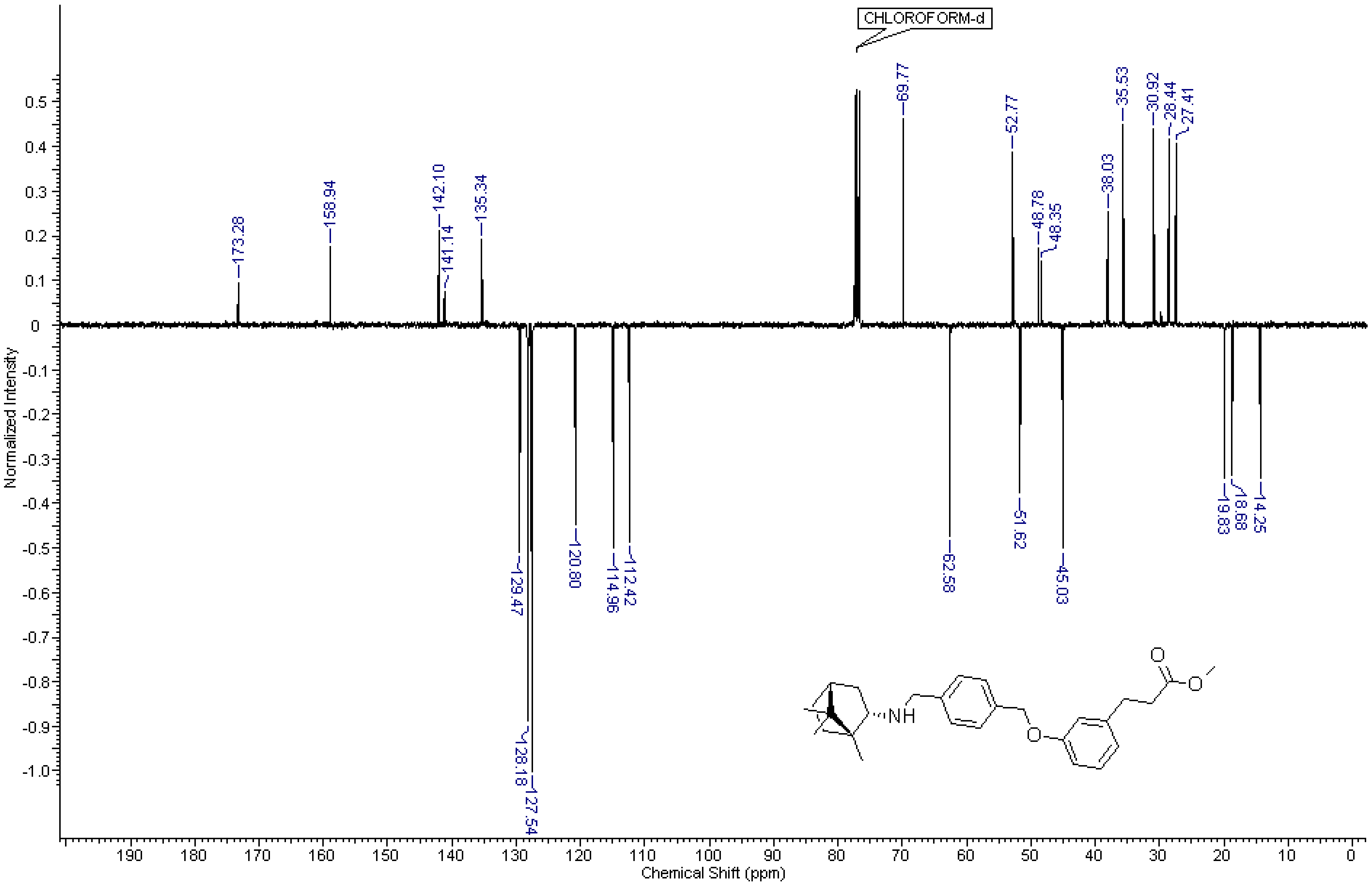

Figure S33. **^{13}C NMR spectrum of 5f (JMOD).**

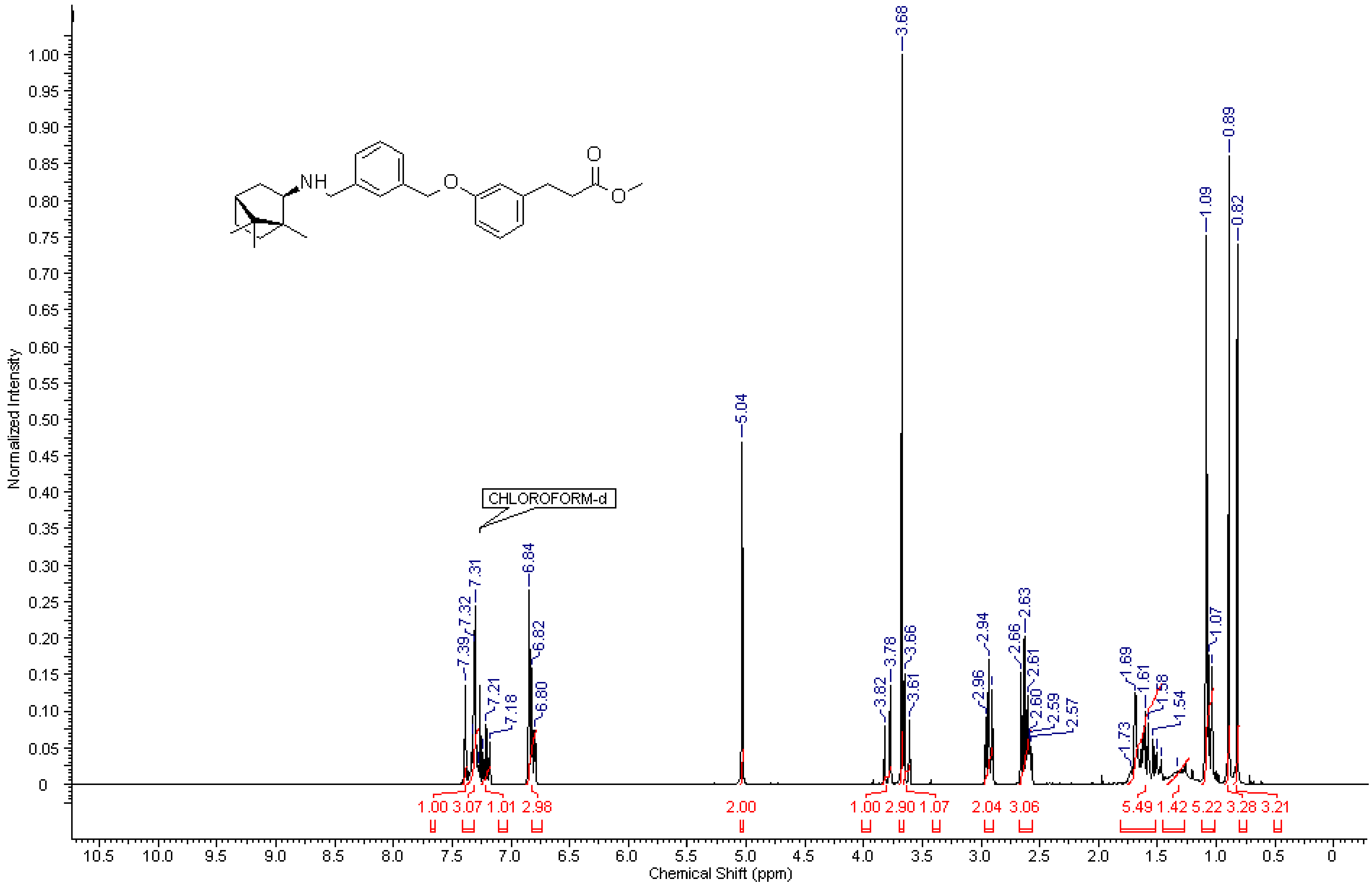

Figure S34. **¹H NMR spectrum of 5g.**

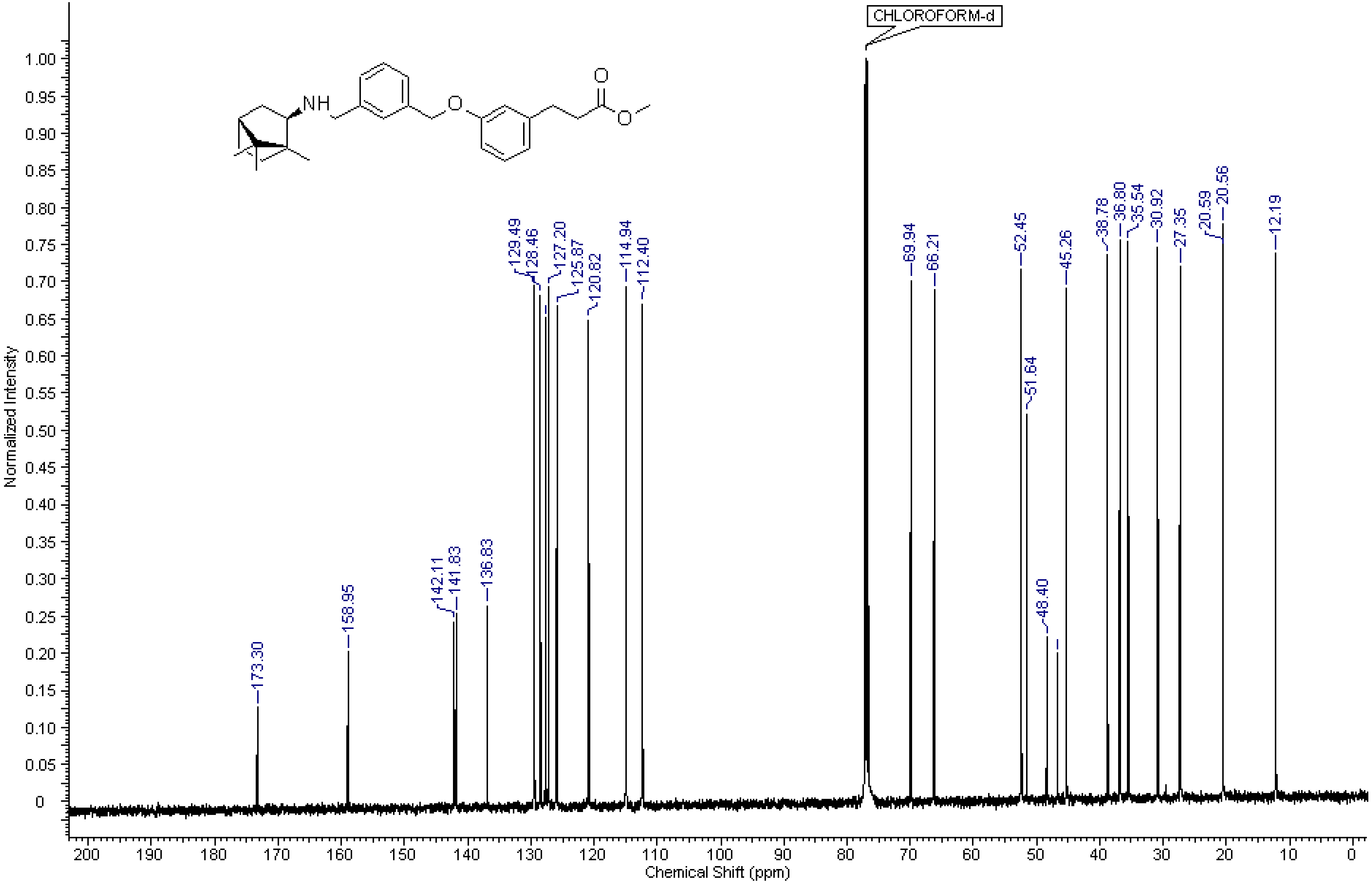

Figure S35. [13]**C NMR spectrum of 5g.**

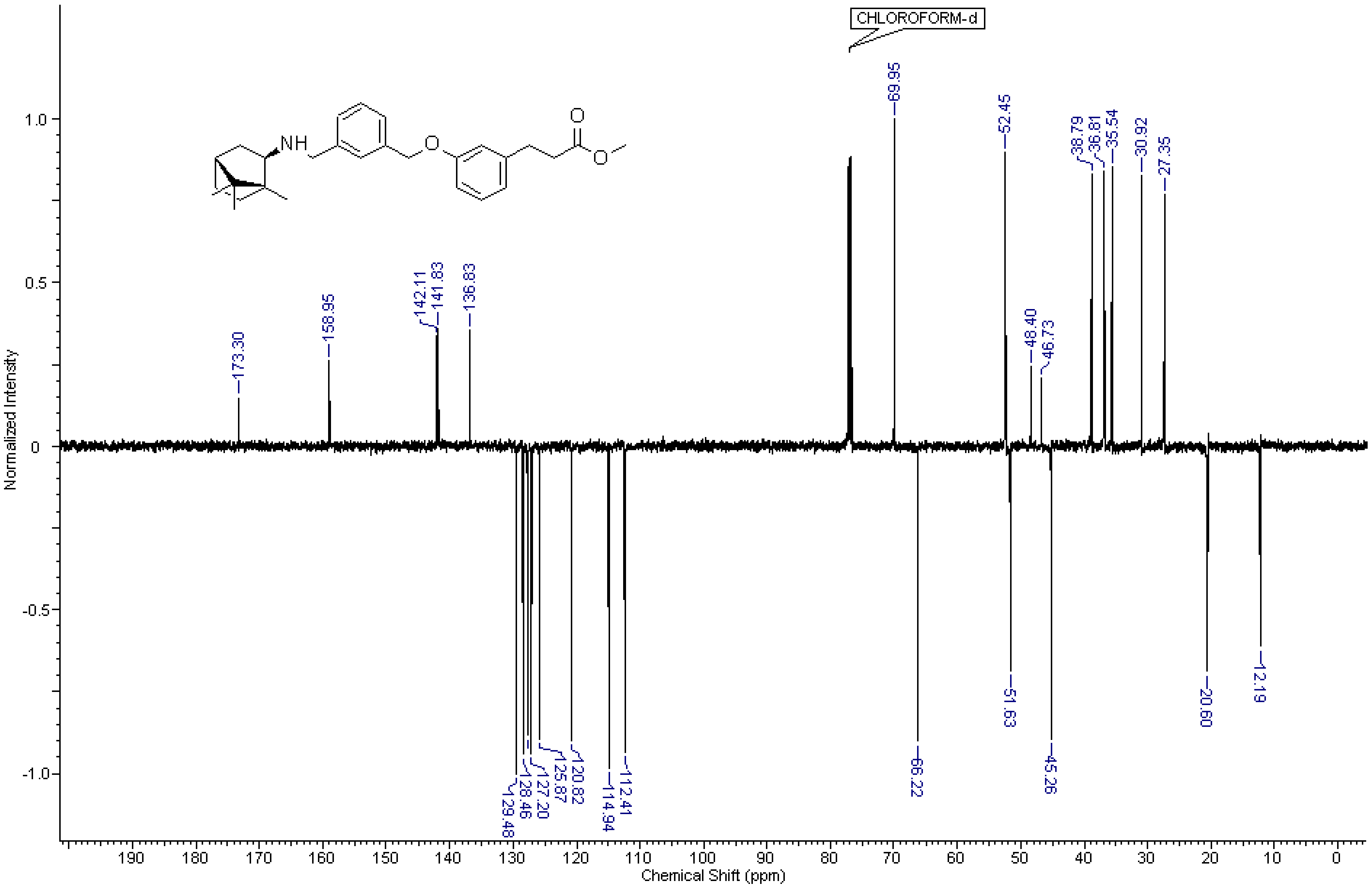

Figure S36. [13]**C NMR spectrum of 5g (JMOD).**

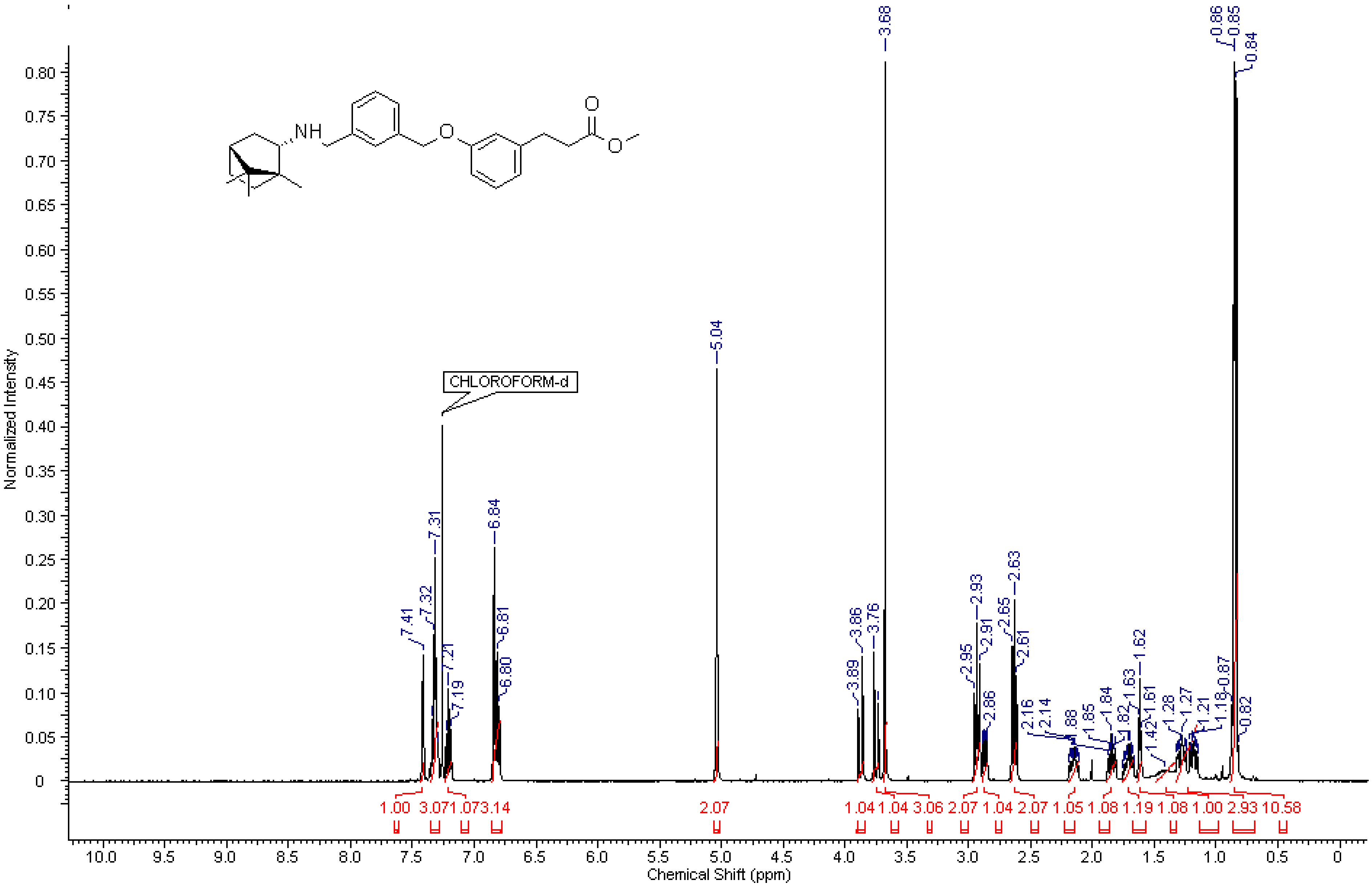

Figure S37. [1]**H NMR spectrum of 5h.**

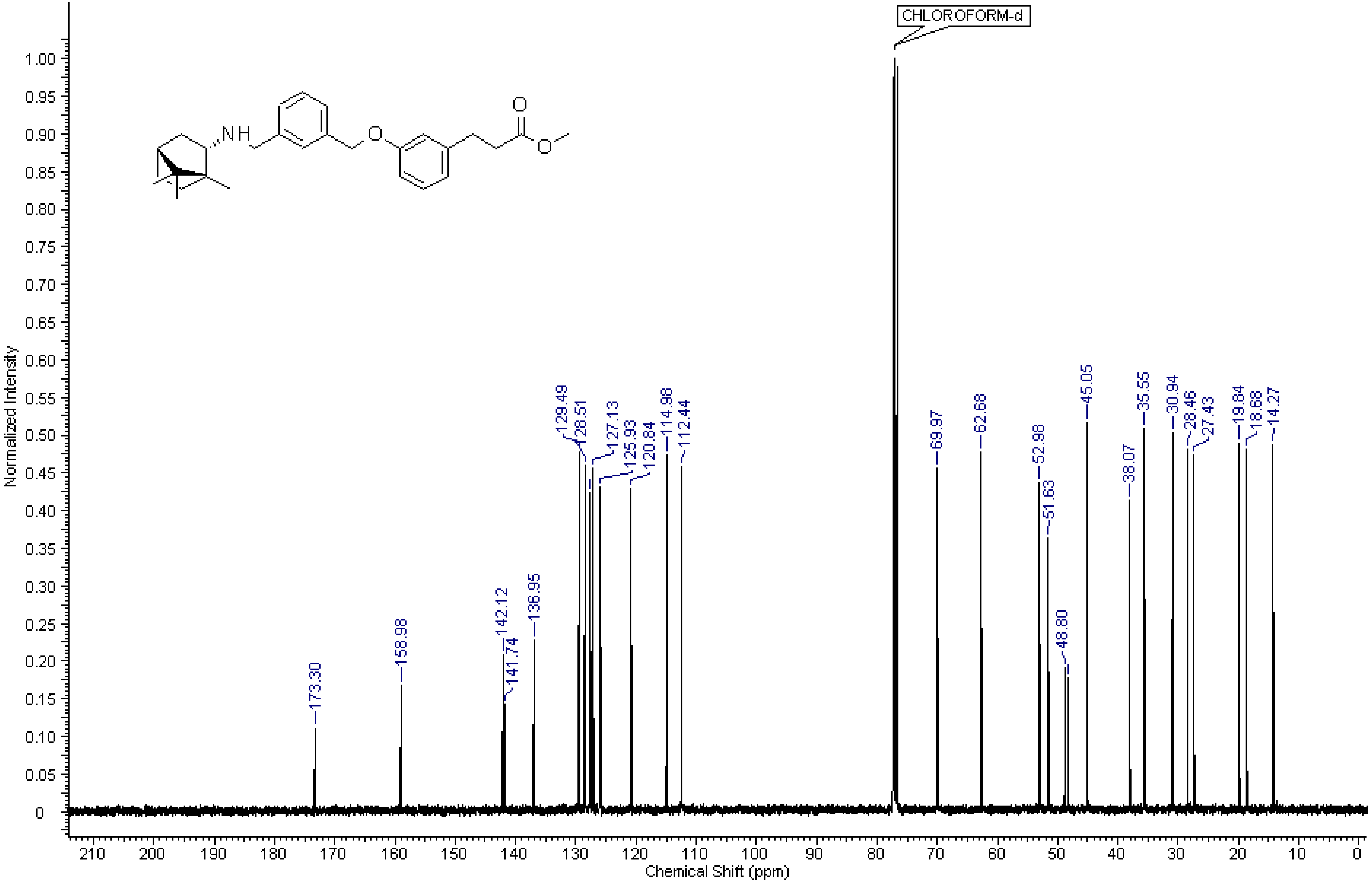

Figure S38. [13]**C NMR spectrum of 5h.**

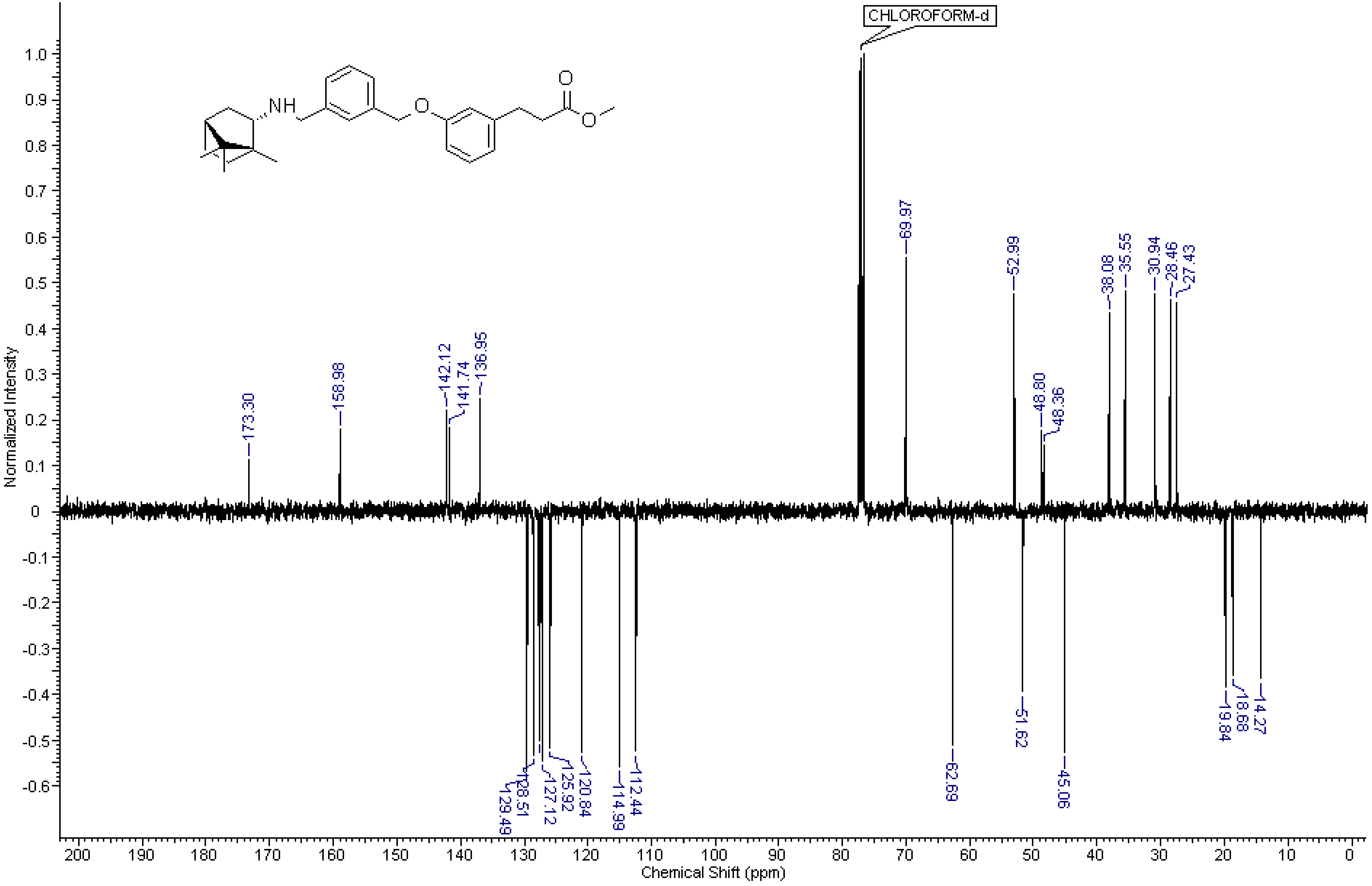

Figure S39. **^{13}C NMR spectrum of 5h (JMOD).**

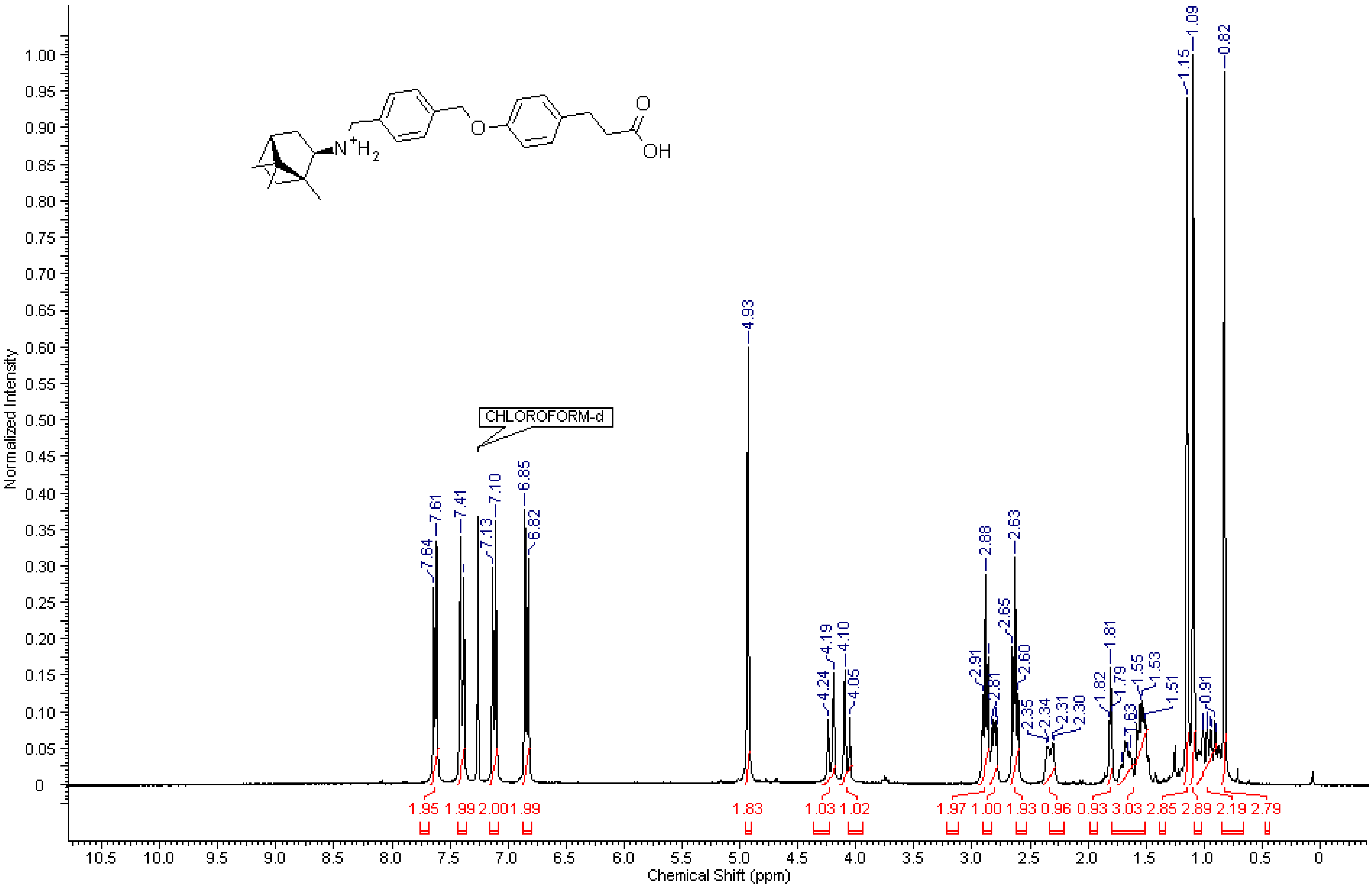

Figure S40. ^{1}H NMR spectrum of **6a**.

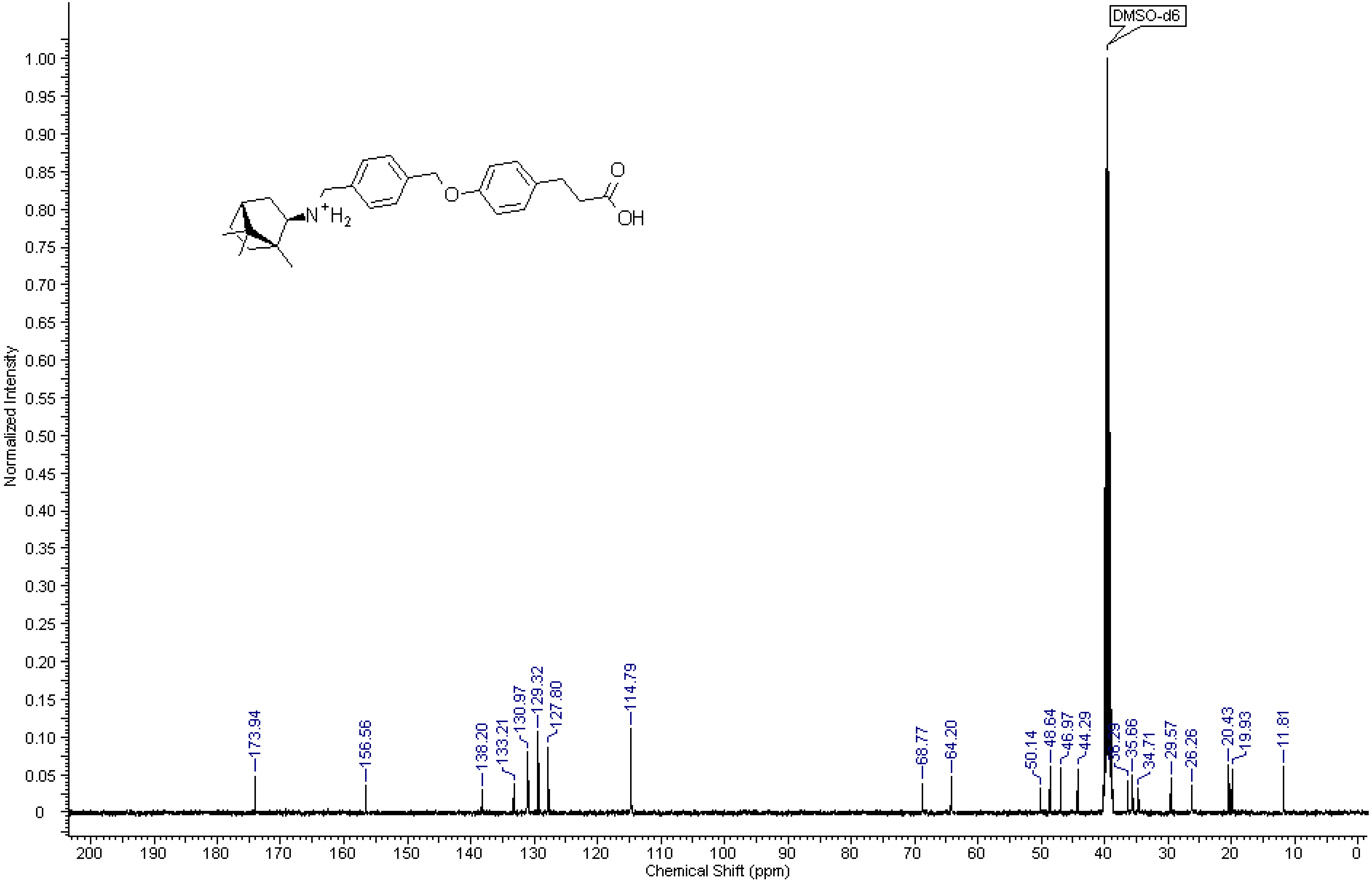

Figure S41. ^{13}C NMR spectrum of **6a.**

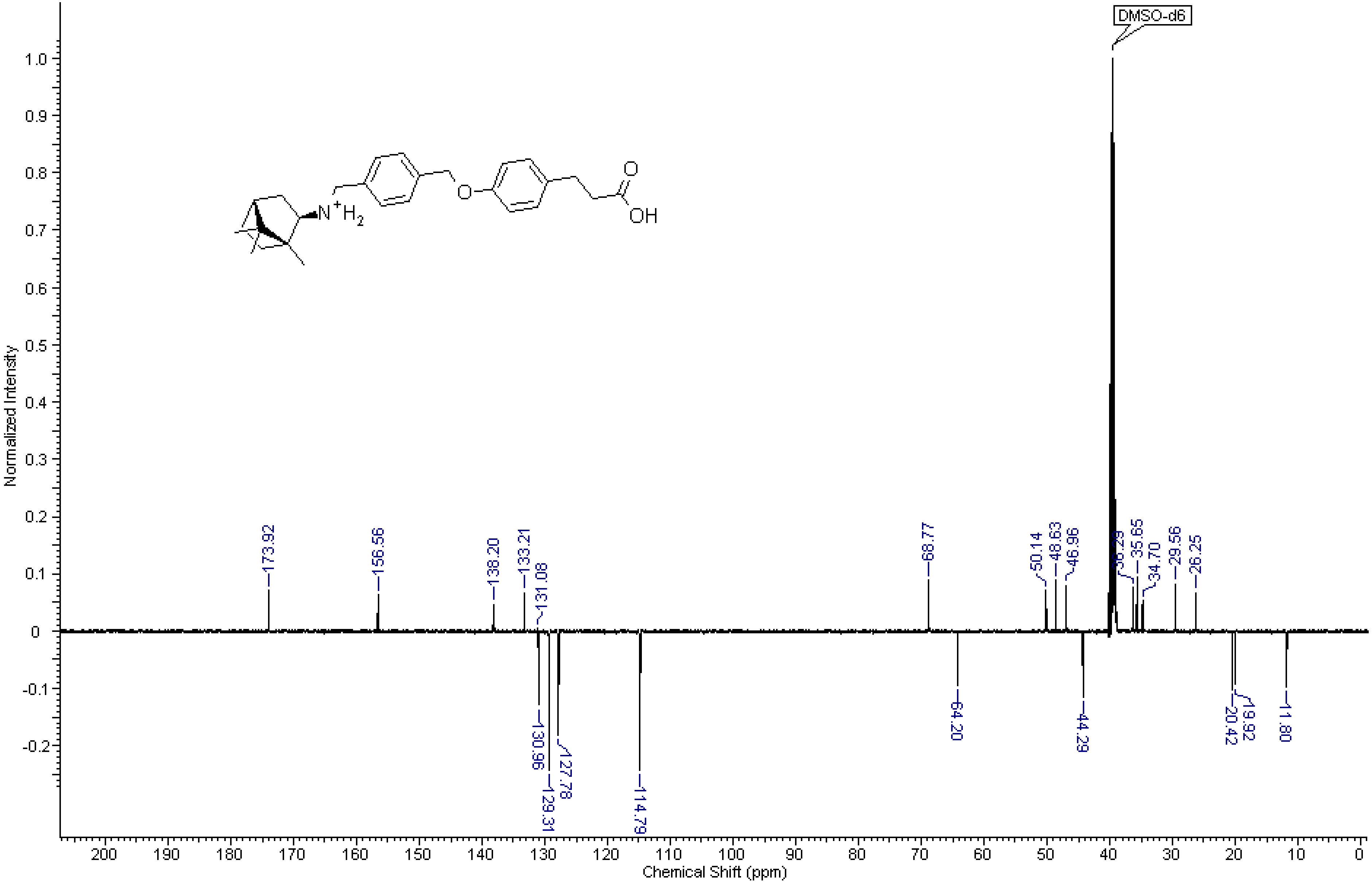

Figure S42. ^{13}C **NMR spectrum of 6a (JMOD).**

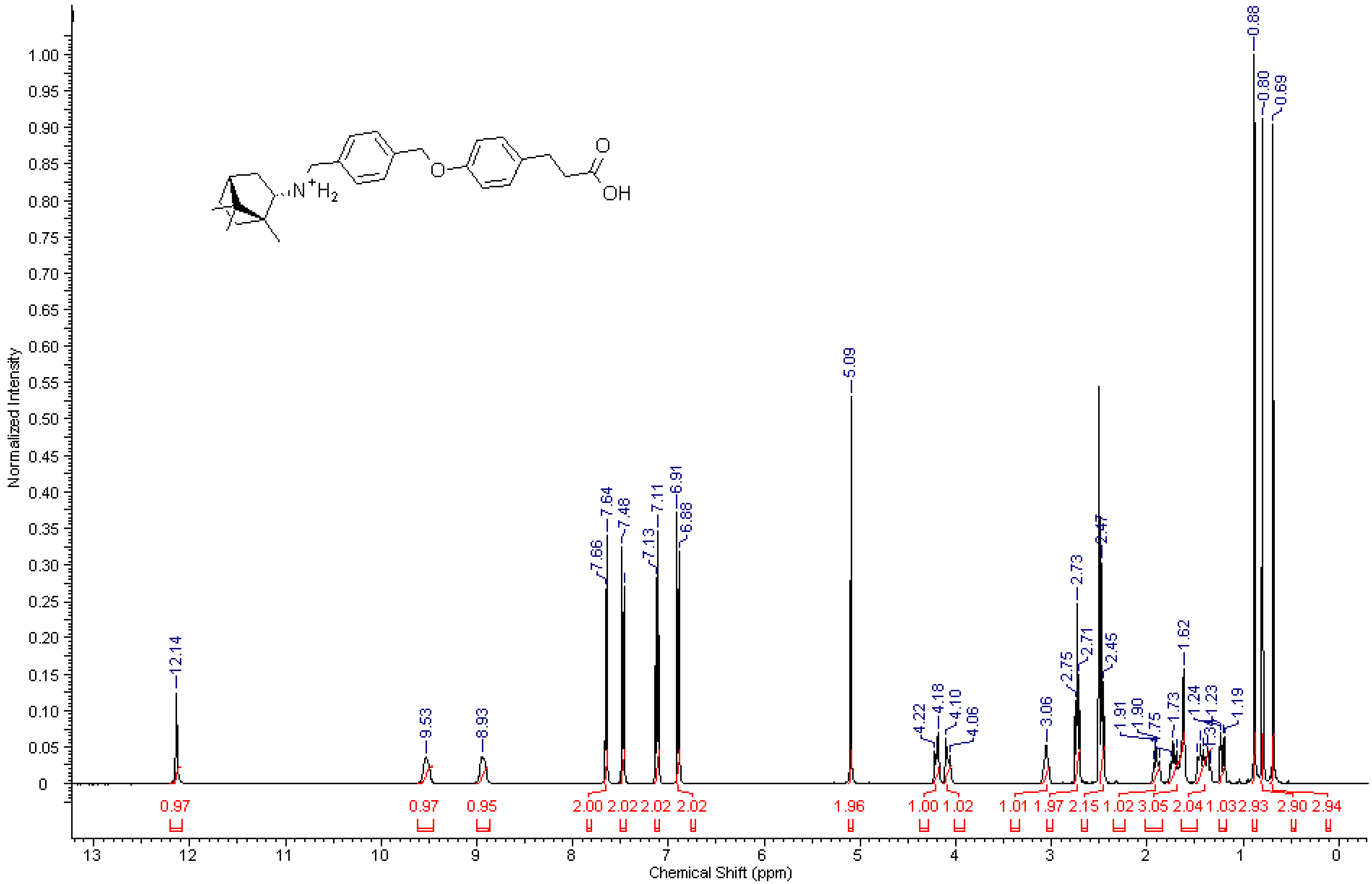

Figure S43. ^{1}H NMR spectrum of **6b**.

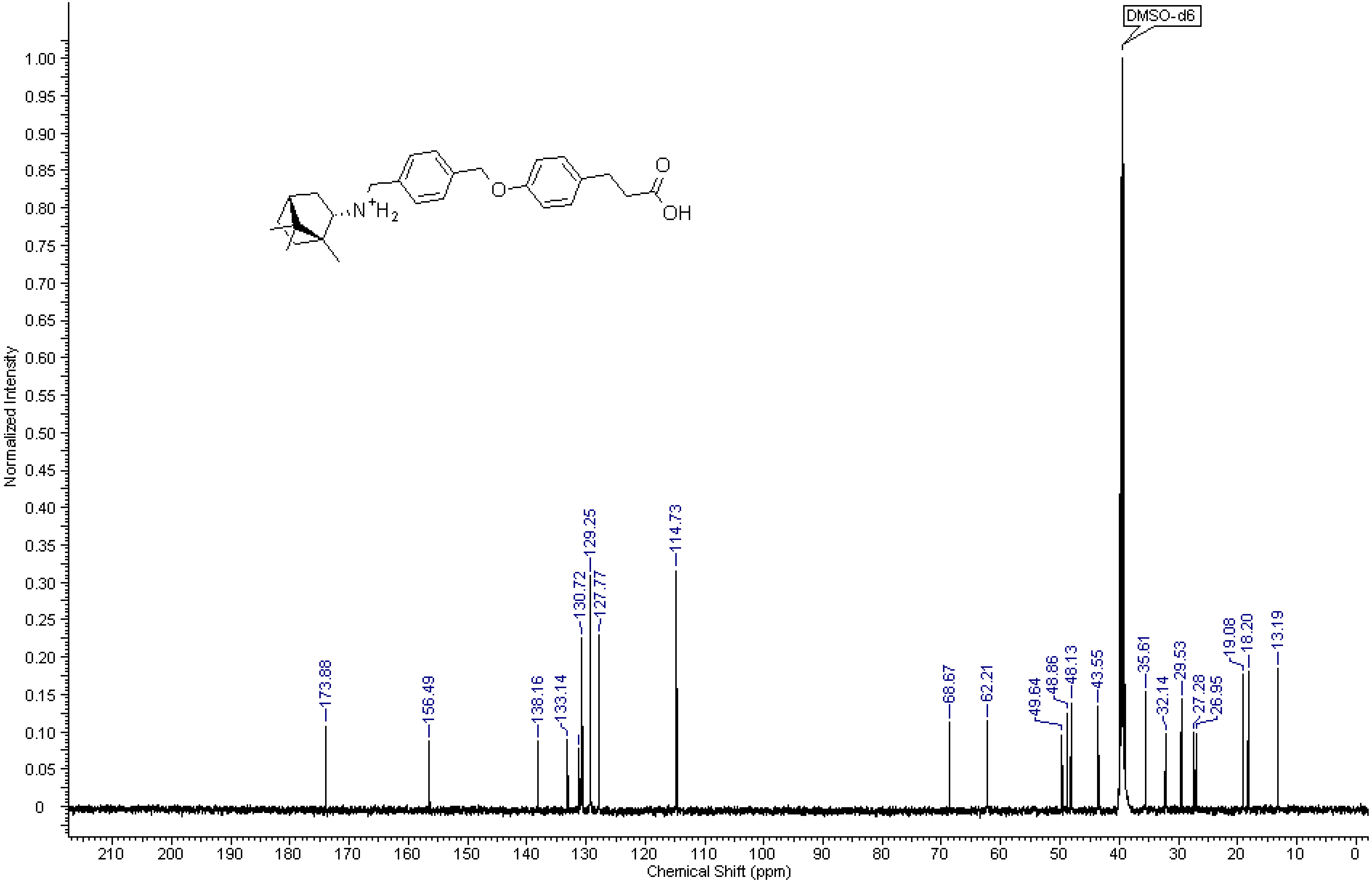

Figure S44. **^{13}C NMR spectrum of 6b.**

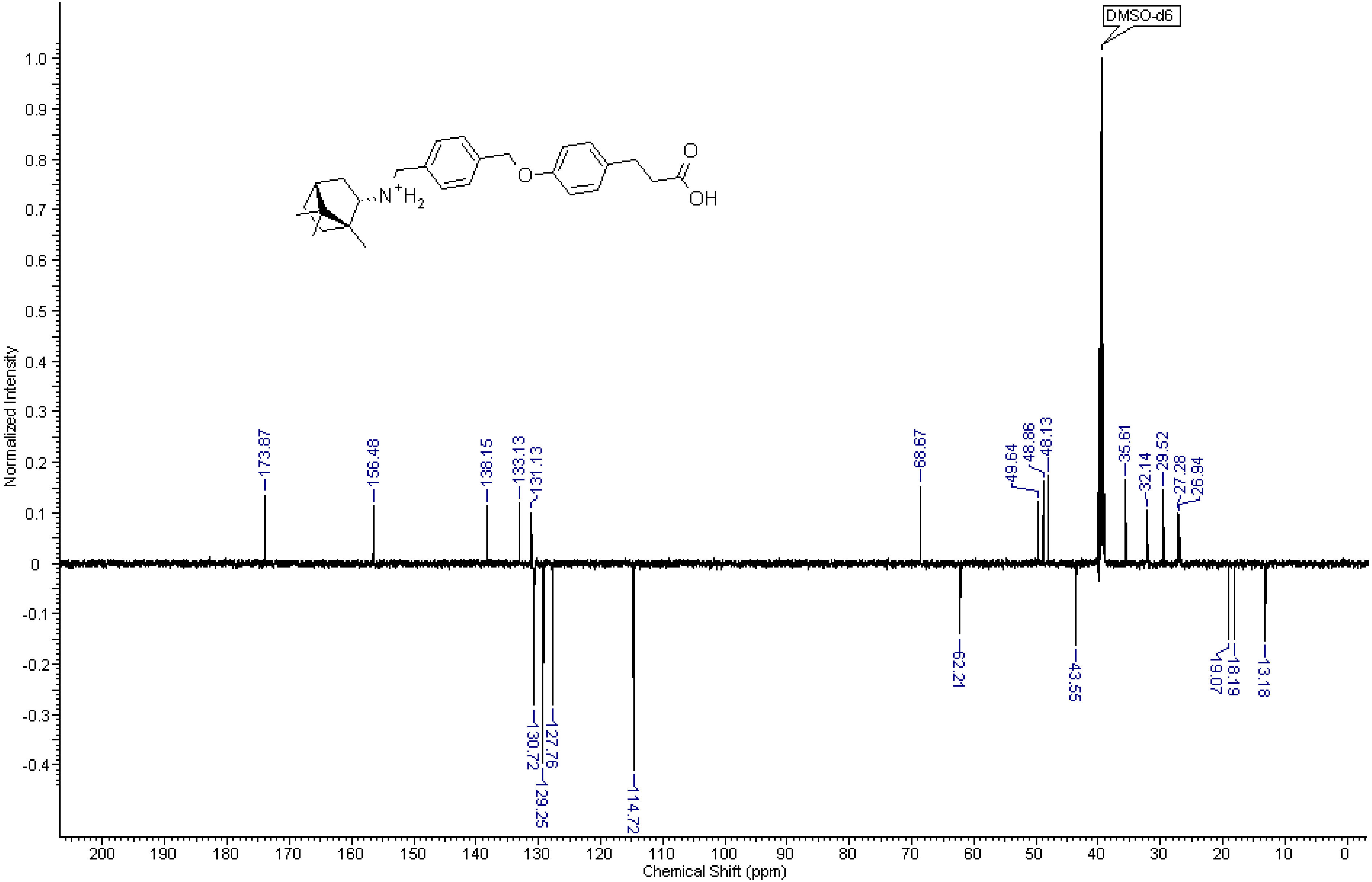

Figure S45. **^{13}C NMR spectrum of 6b (JMOD).**

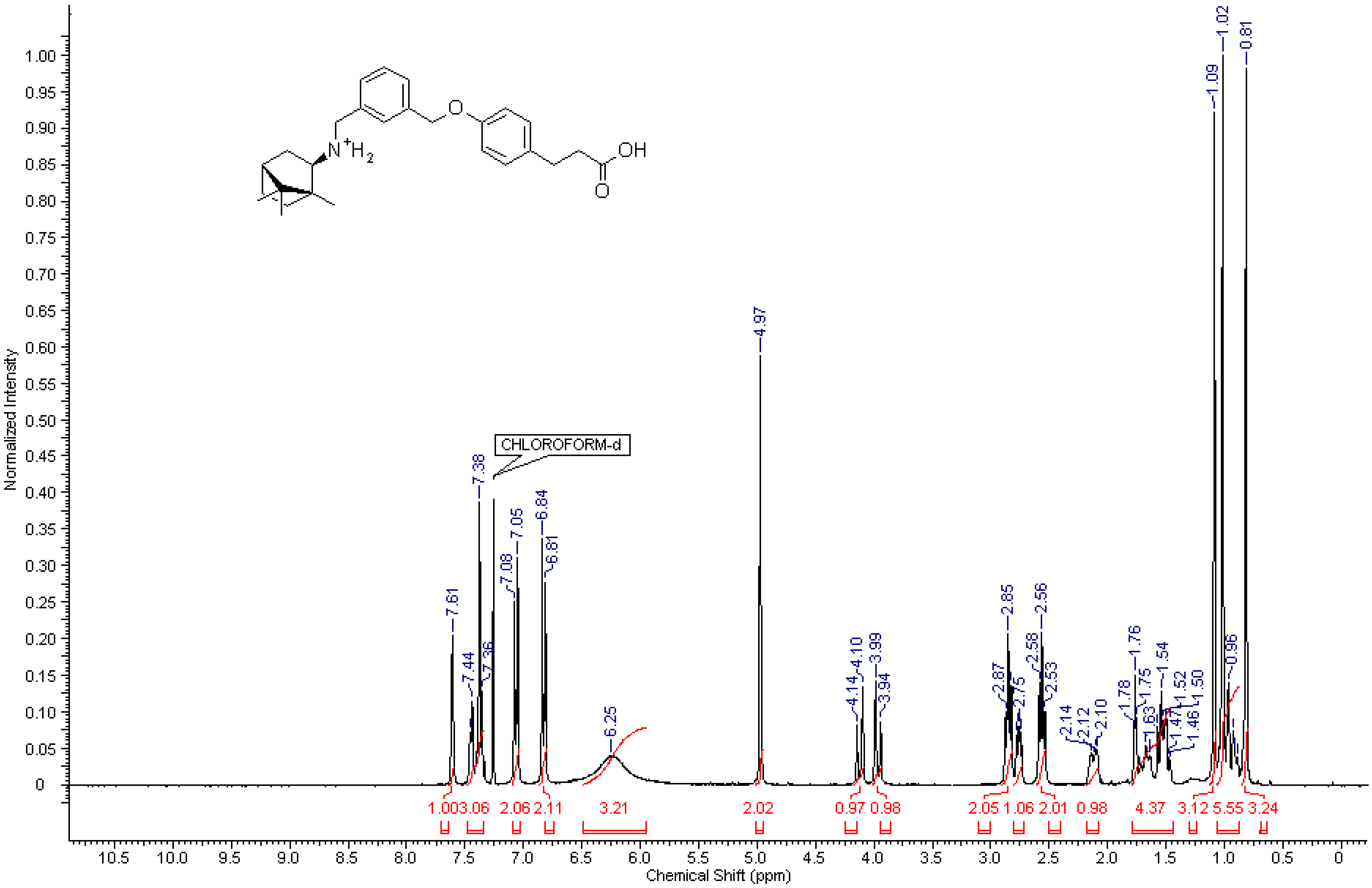

Figure S46. ^{1}**H NMR spectrum of 6c.**

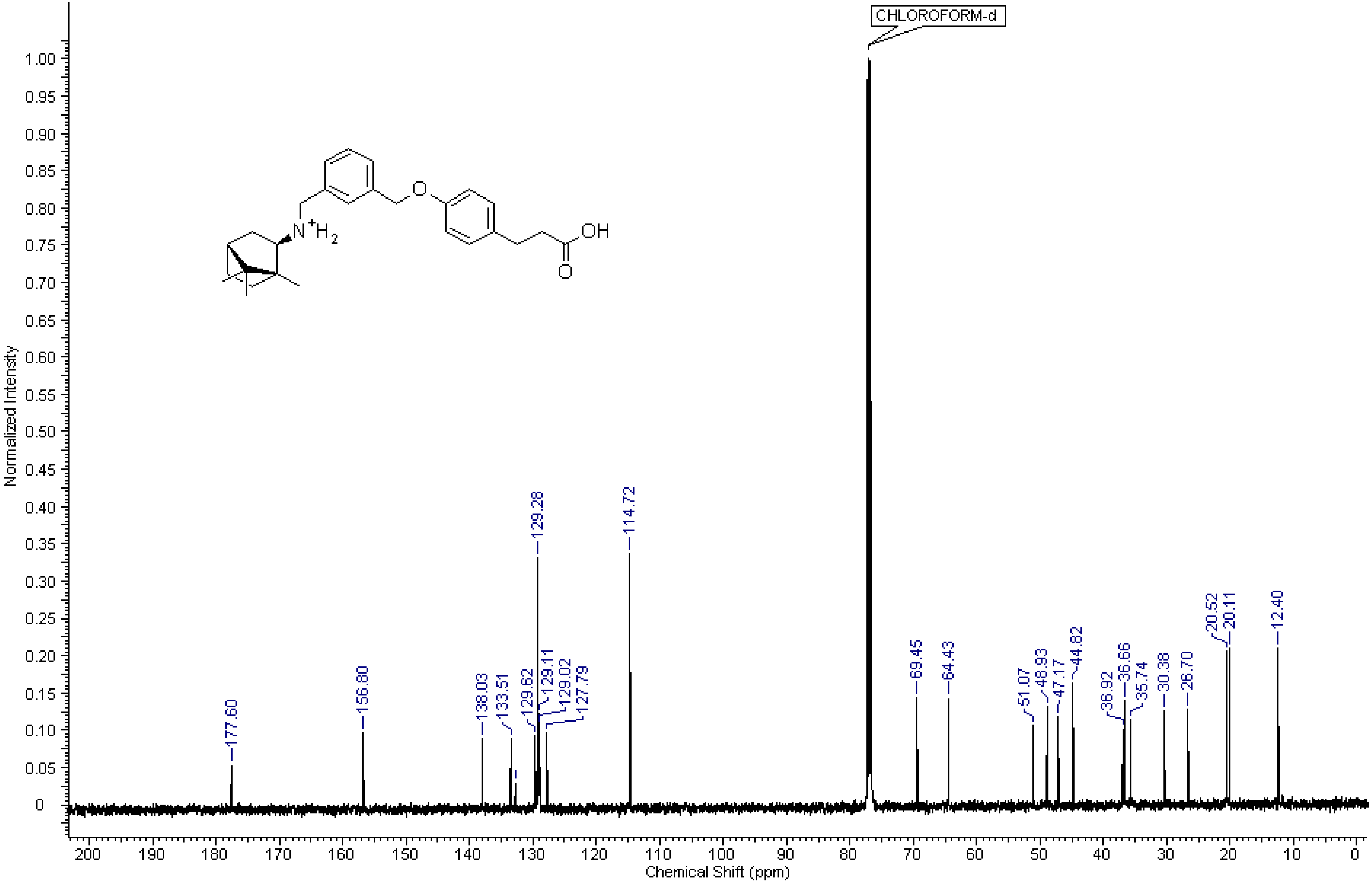

Figure S47. ^{13}**C NMR spectrum of 6c.**

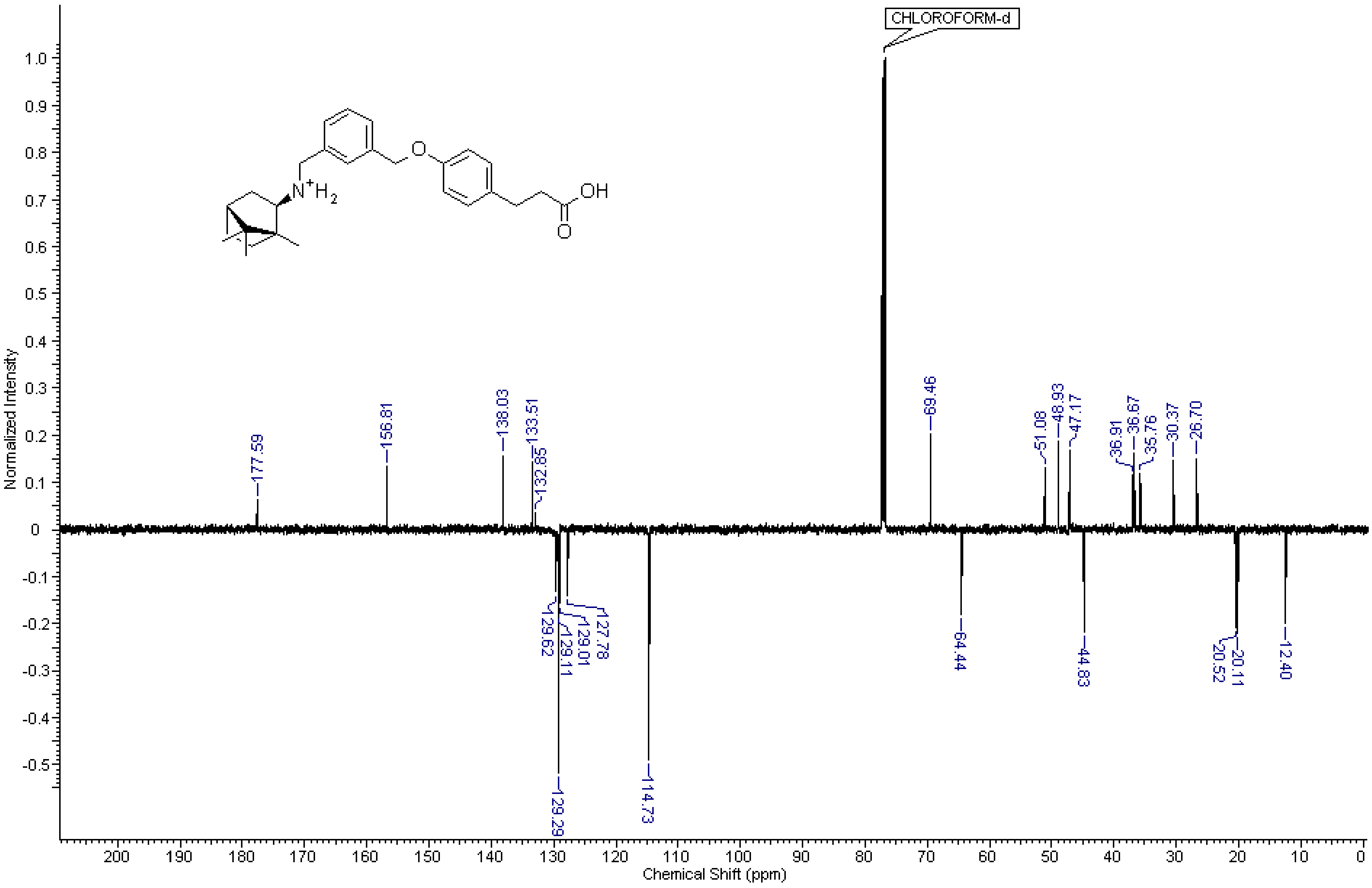

Figure S48. ^{13}C **NMR spectrum of 6c (JMOD).**

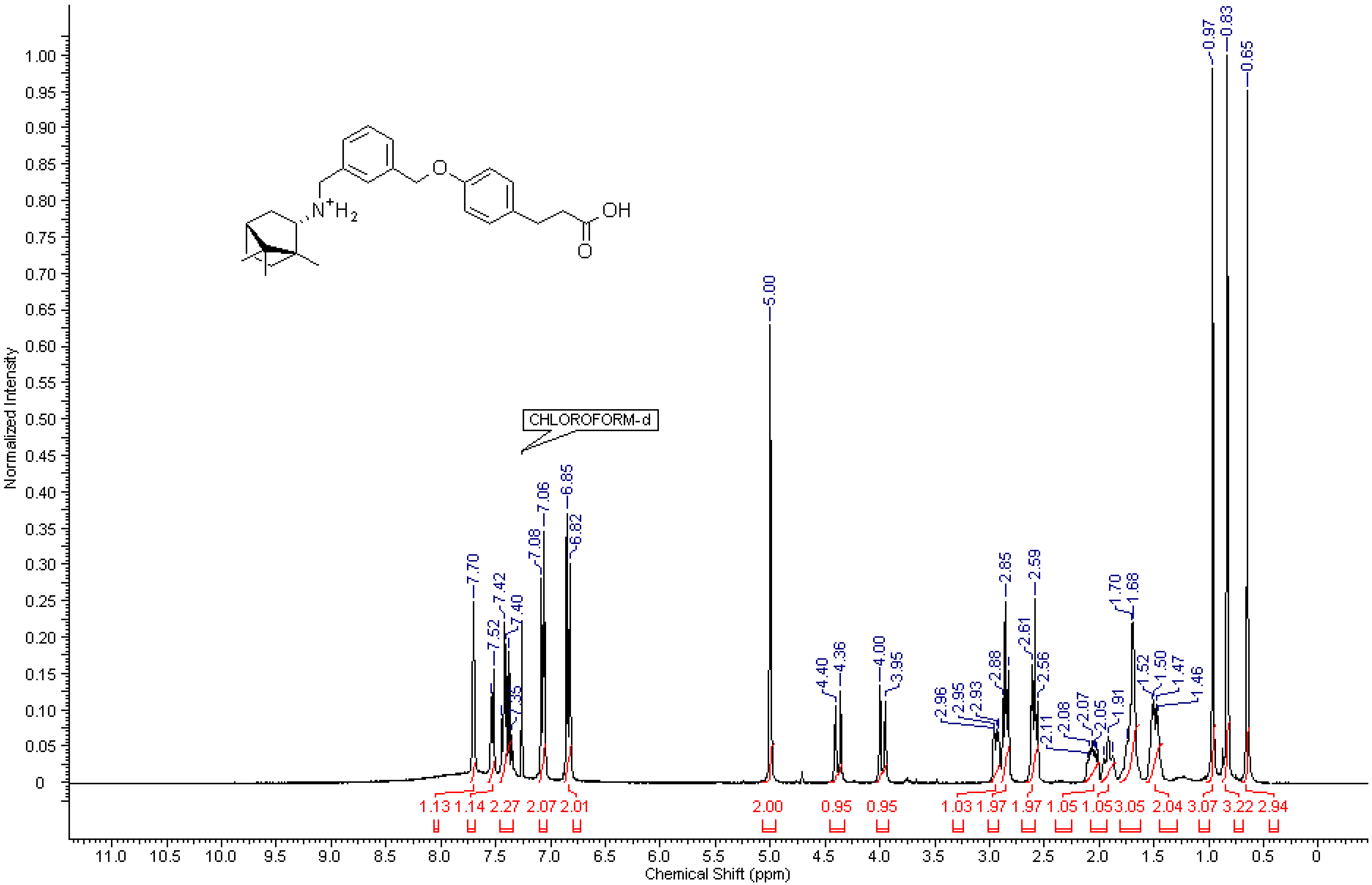

Figure S49. ^{1}H NMR spectrum of 6d.

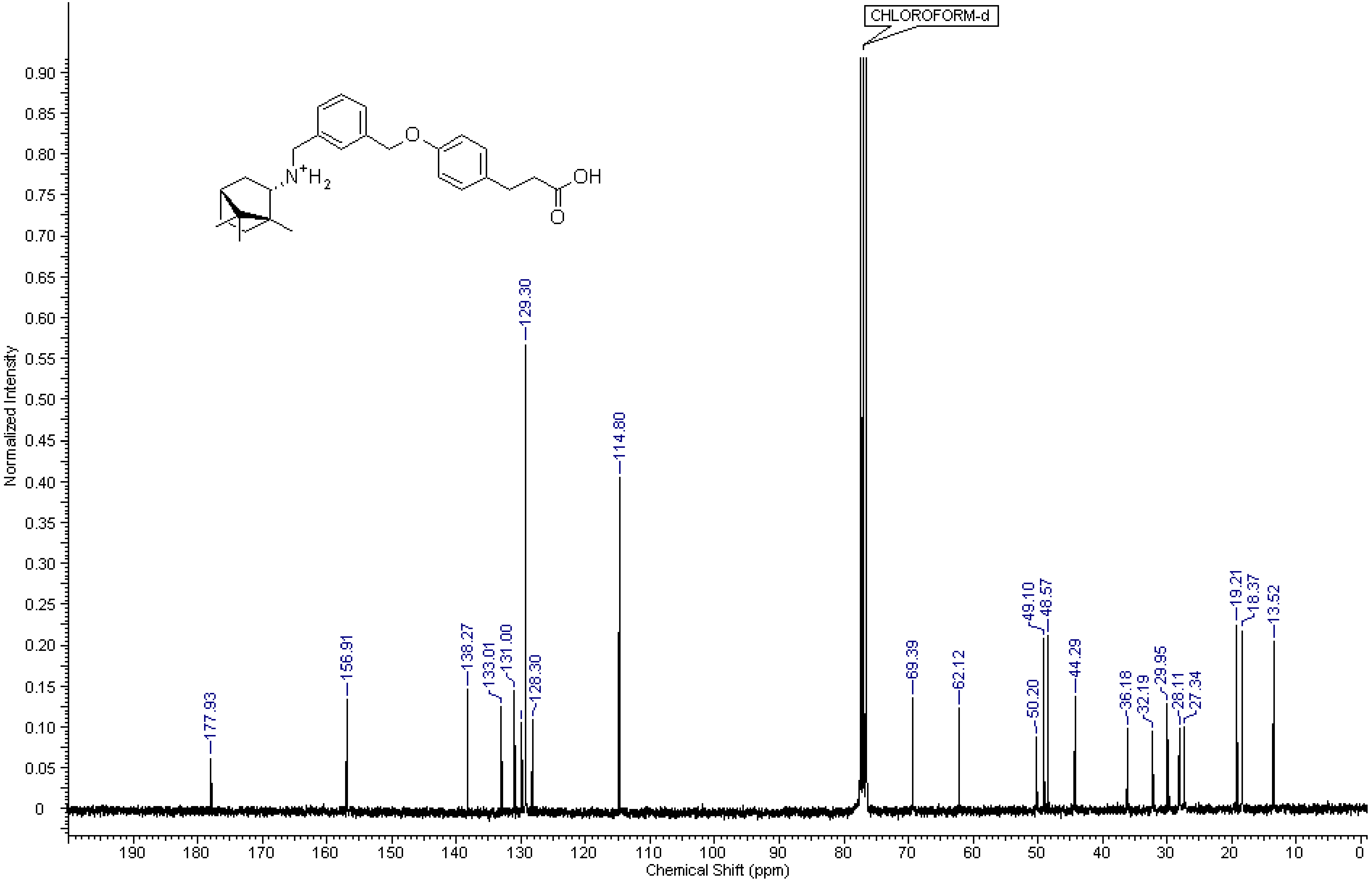

Figure S50. [13]**C NMR spectrum of 6d.**

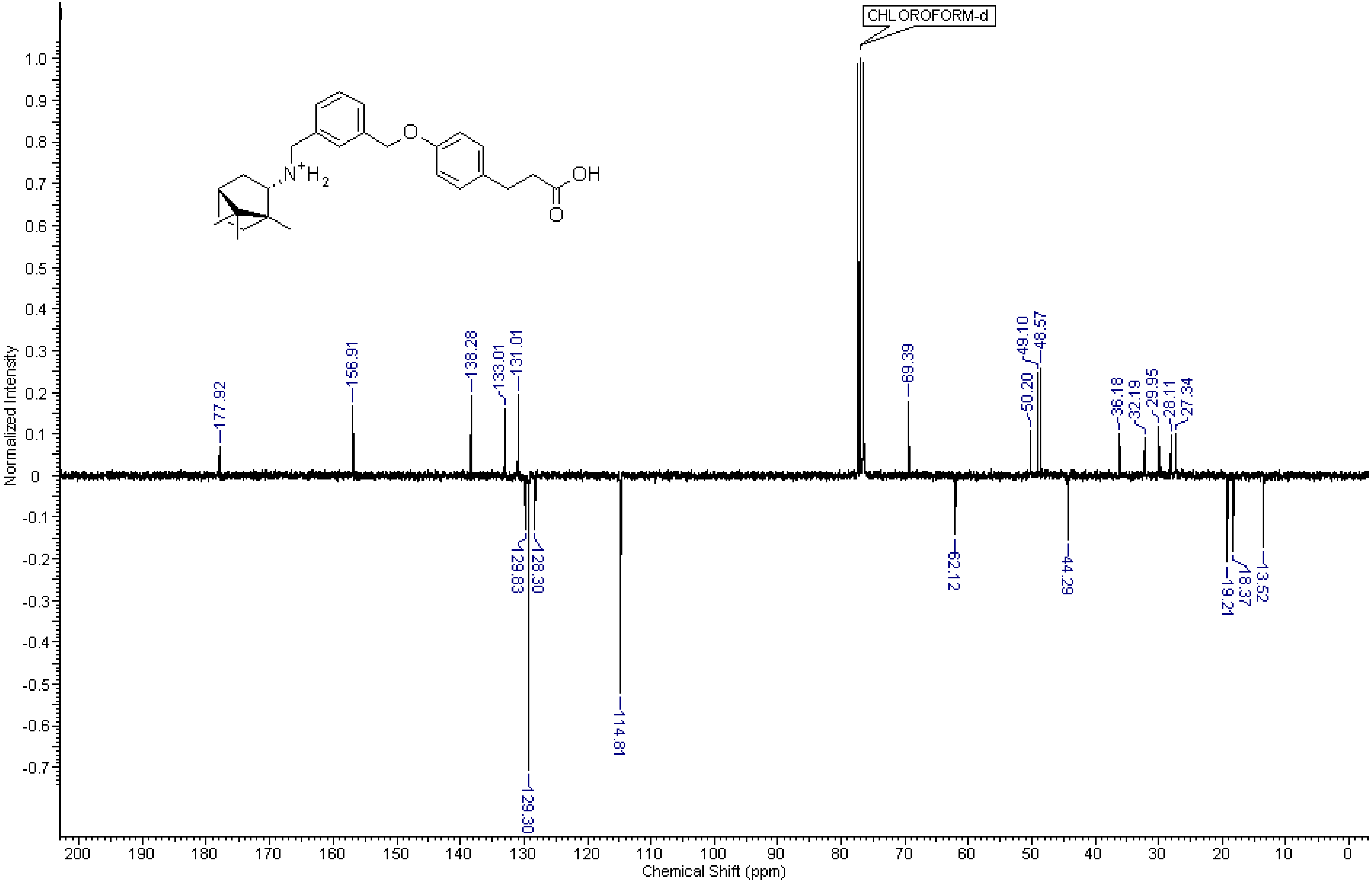

Figure S51. ^{13}**C NMR spectrum of 6d (JMOD).**

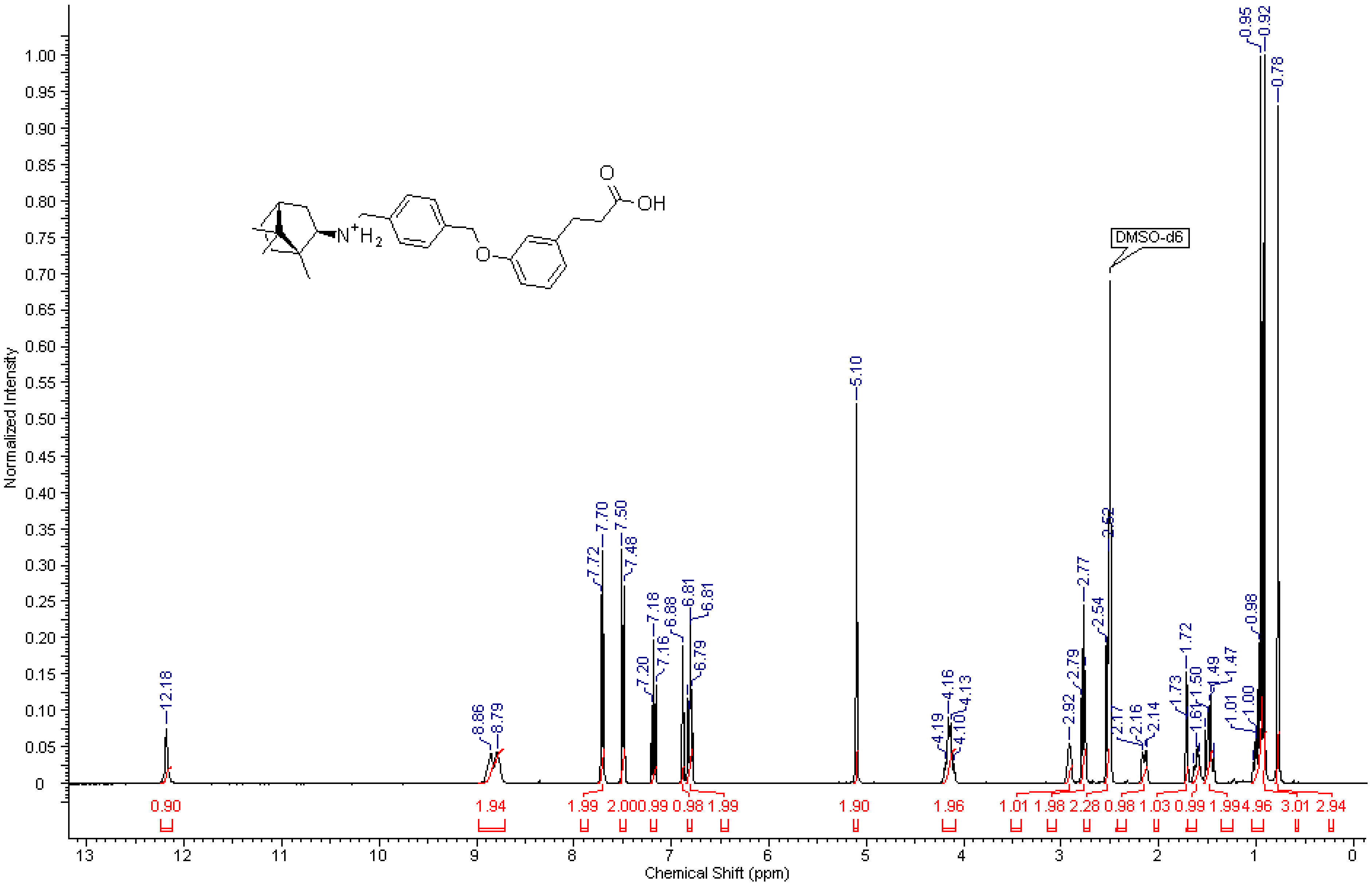

Figure S52. ^{1}**H NMR spectrum of 6e.**

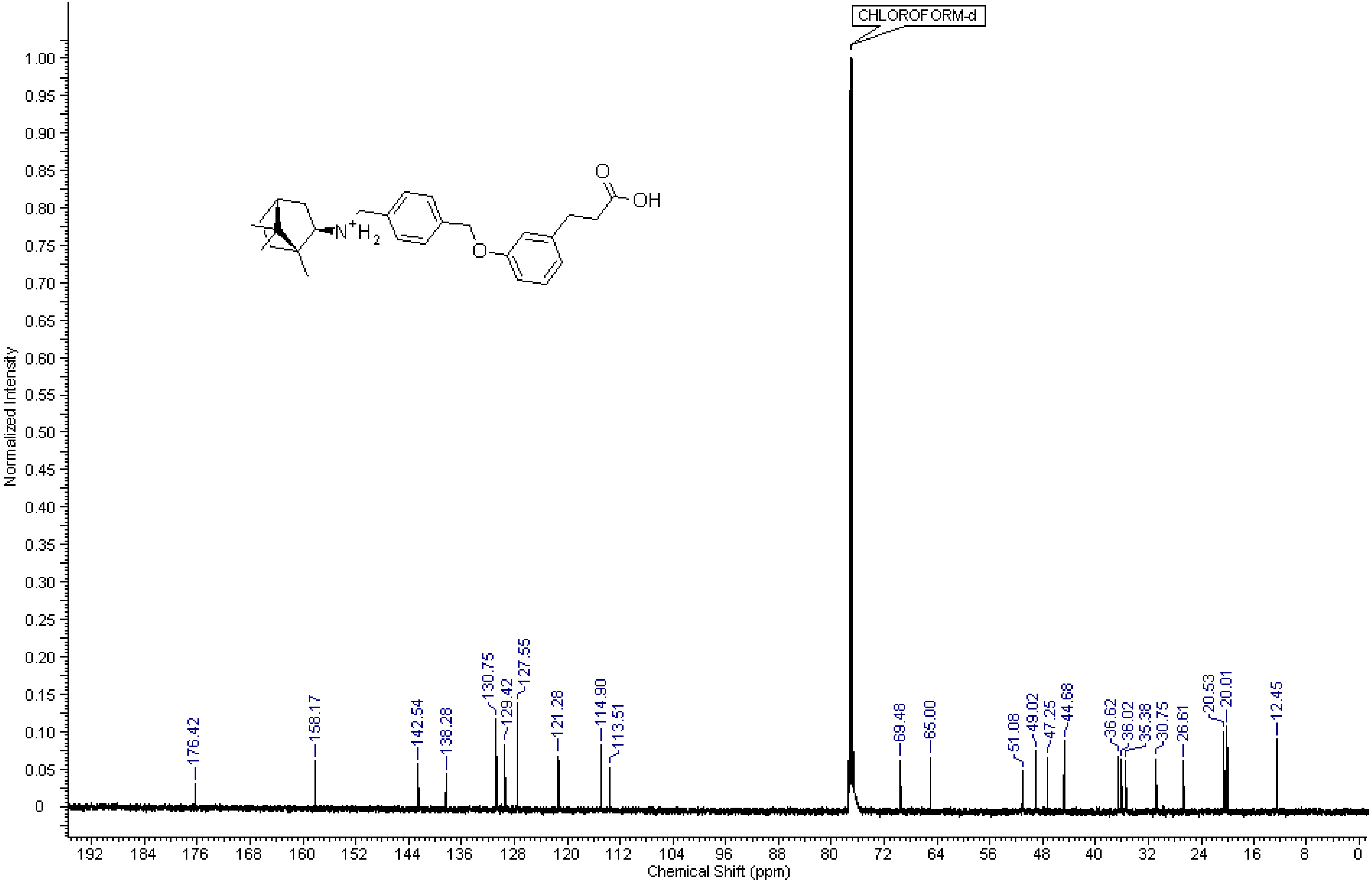

Figure S53. ^{13}**C NMR spectrum of 6e.**

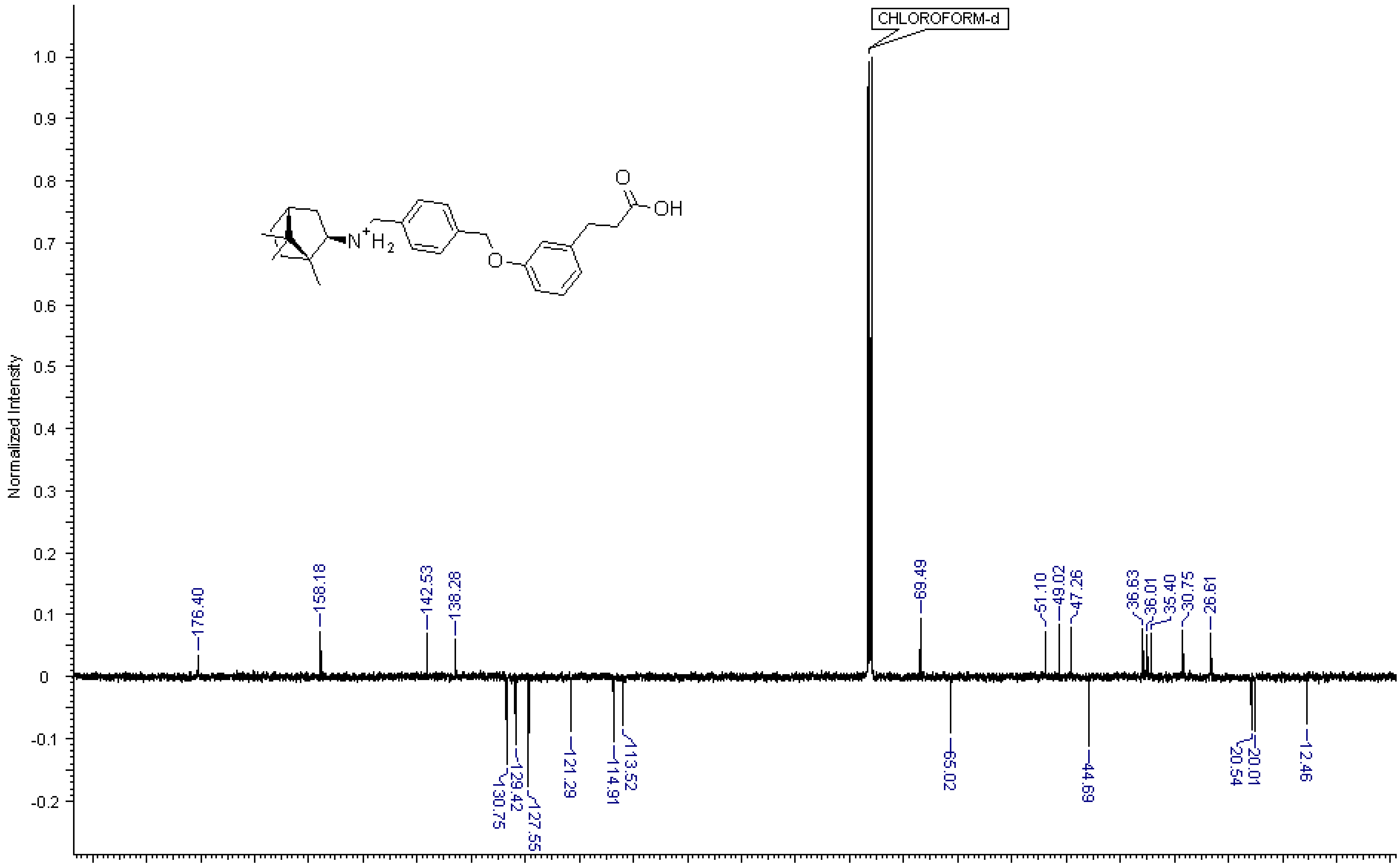

Figure S54. ^{13}**C NMR spectrum of 6e (JMOD).**

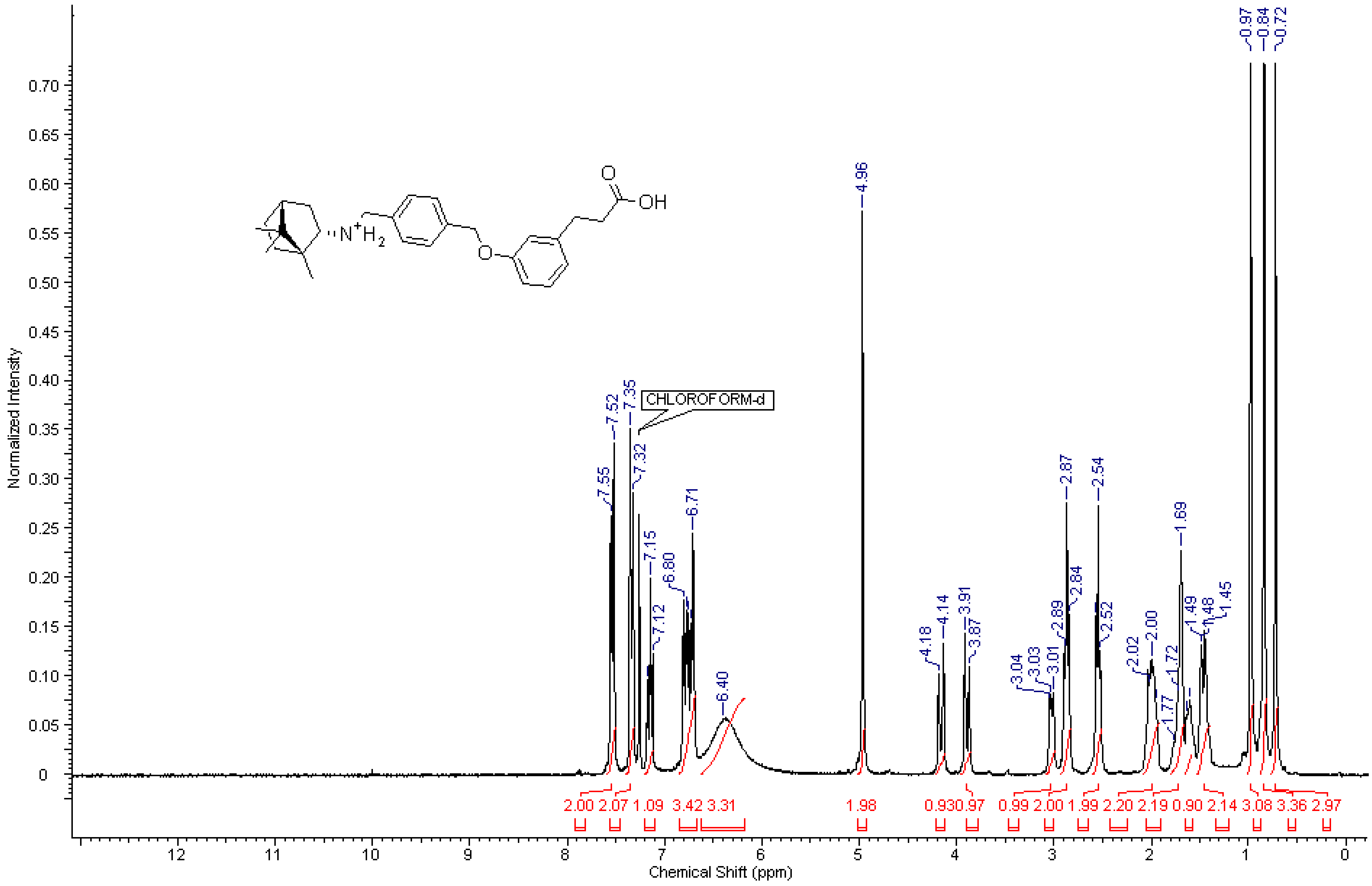

Figure S55. ^{1}**H NMR spectrum of 6f.**

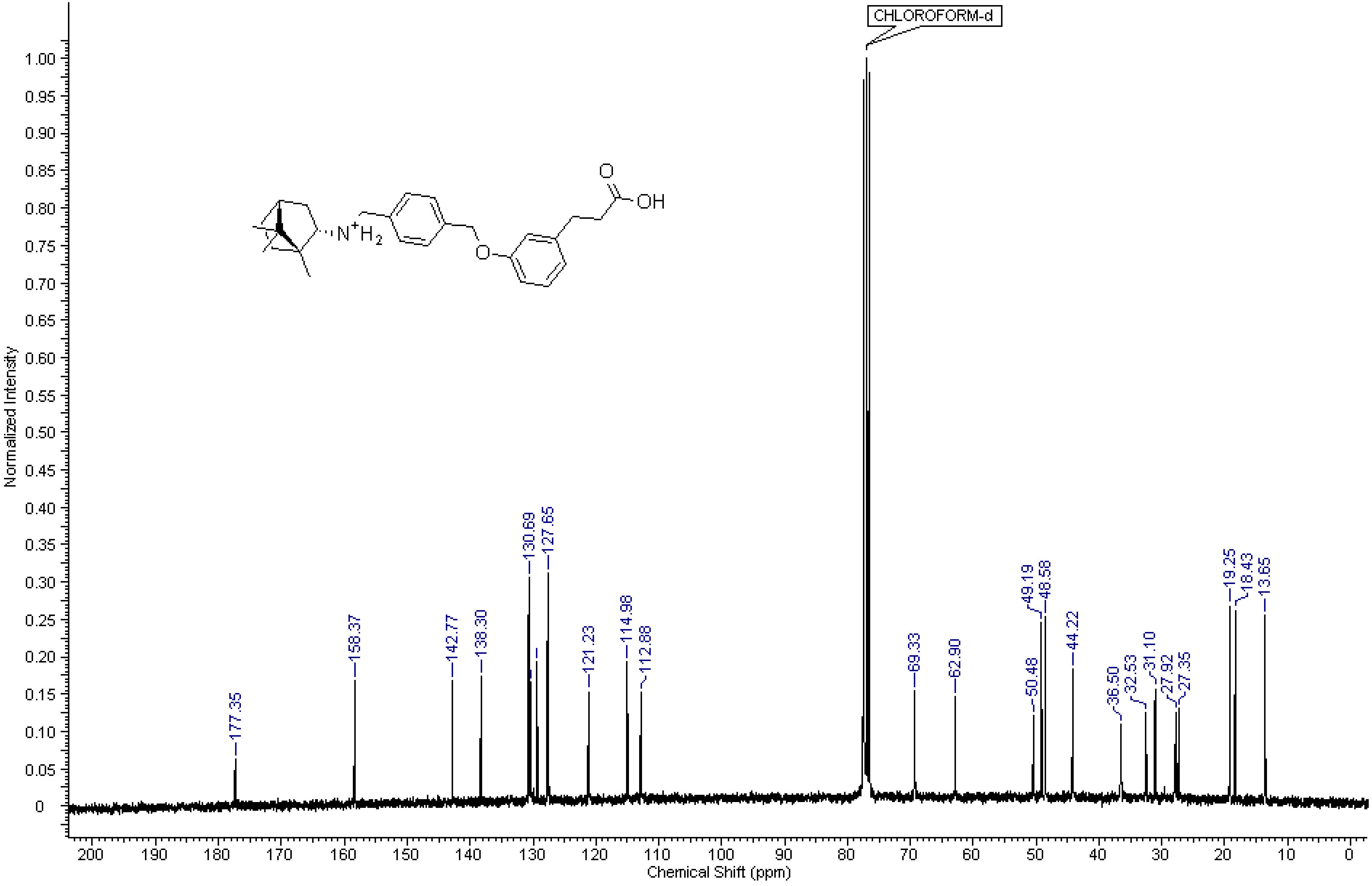

Figure S56. **^{13}C NMR spectrum of 6f.**

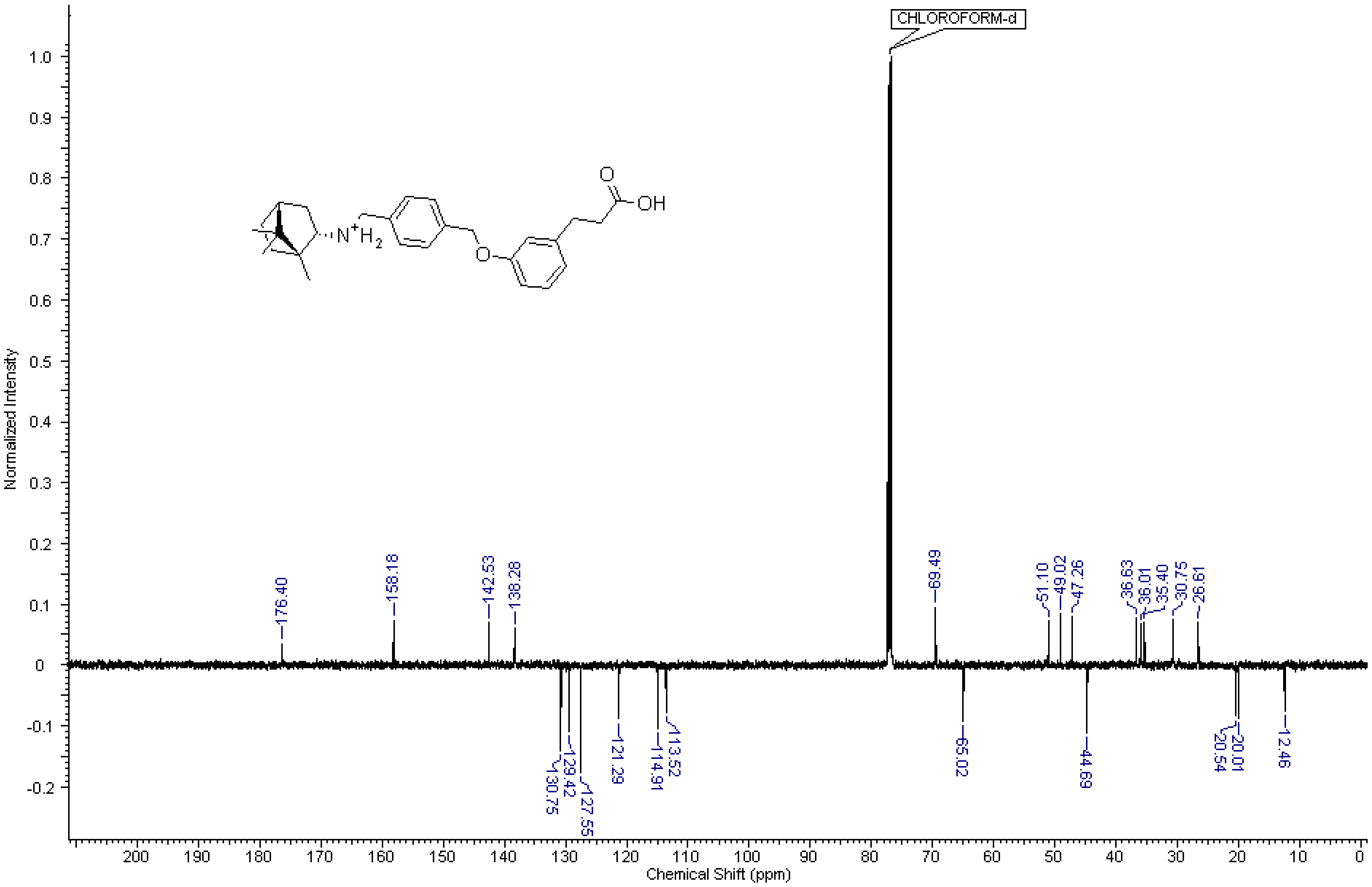

Figure S57. ^{13}**C NMR spectrum of 6f (JMOD).**

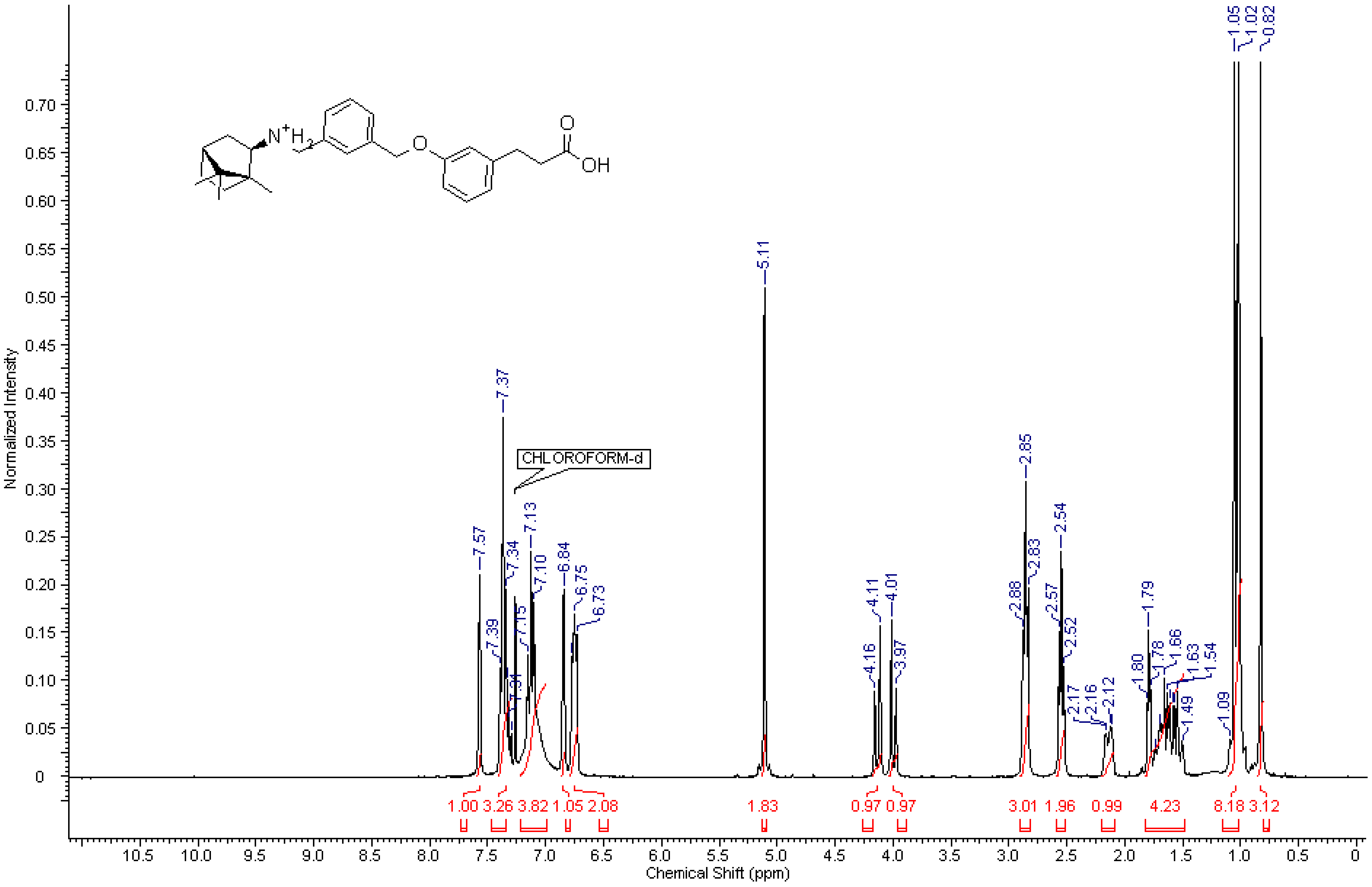

Figure S58. ^{1}H NMR spectrum of 6g.

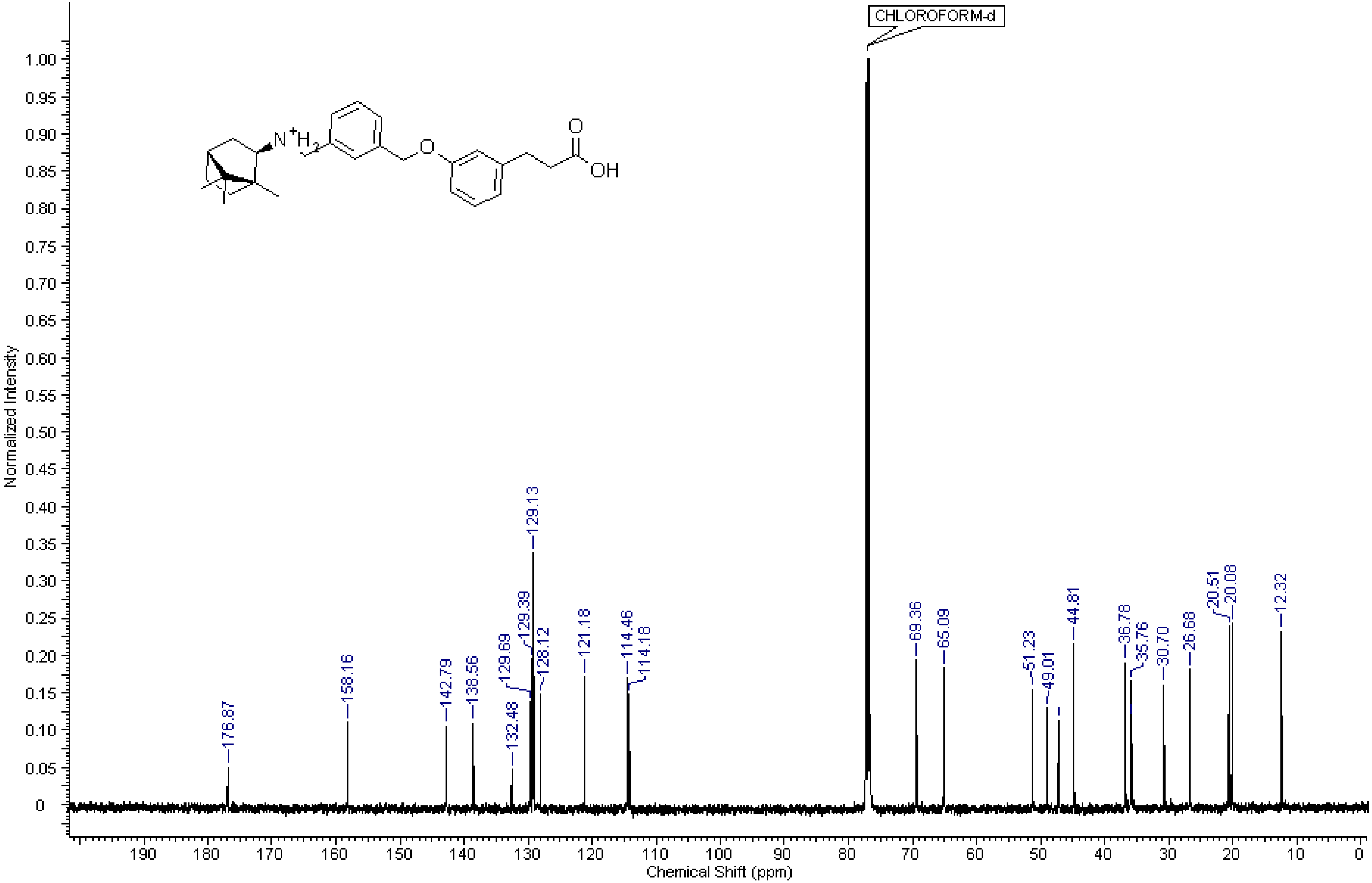

Figure S59. **^{13}C NMR spectrum of 6g.**

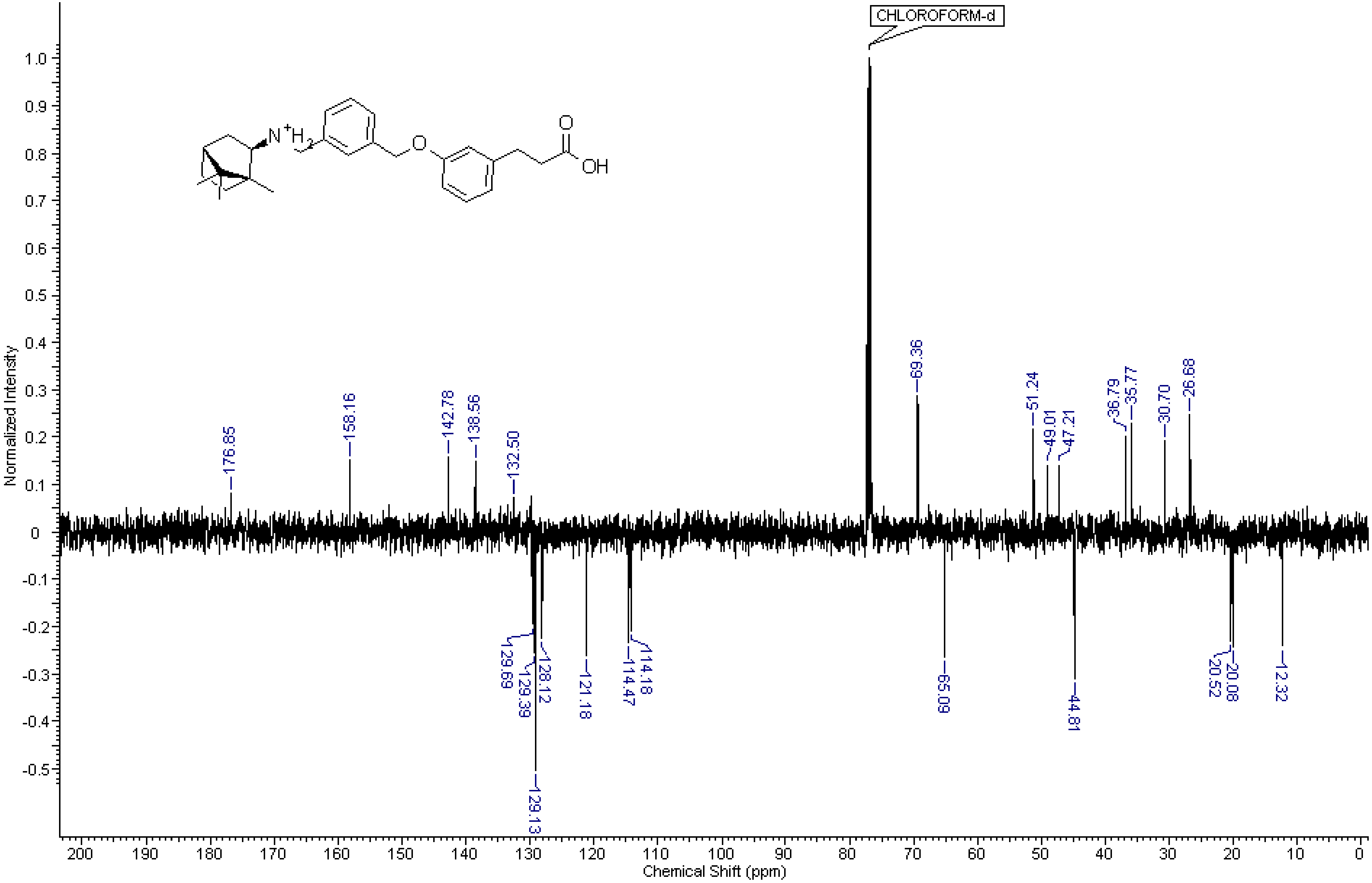

Figure S60. **^{13}C NMR spectrum of 6g (JMOD).**

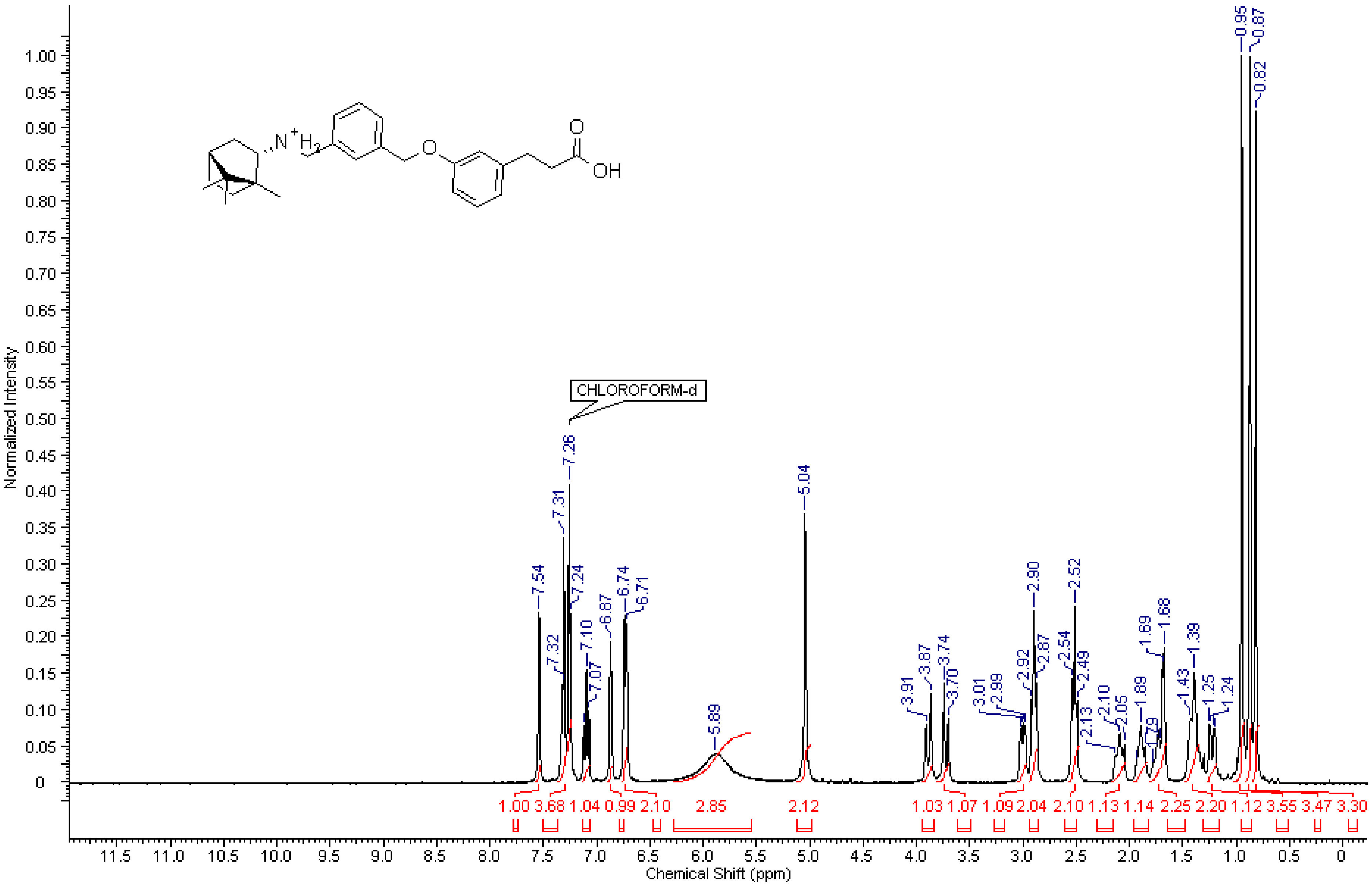

Figure S61. [1]**H NMR spectrum of 6h.**

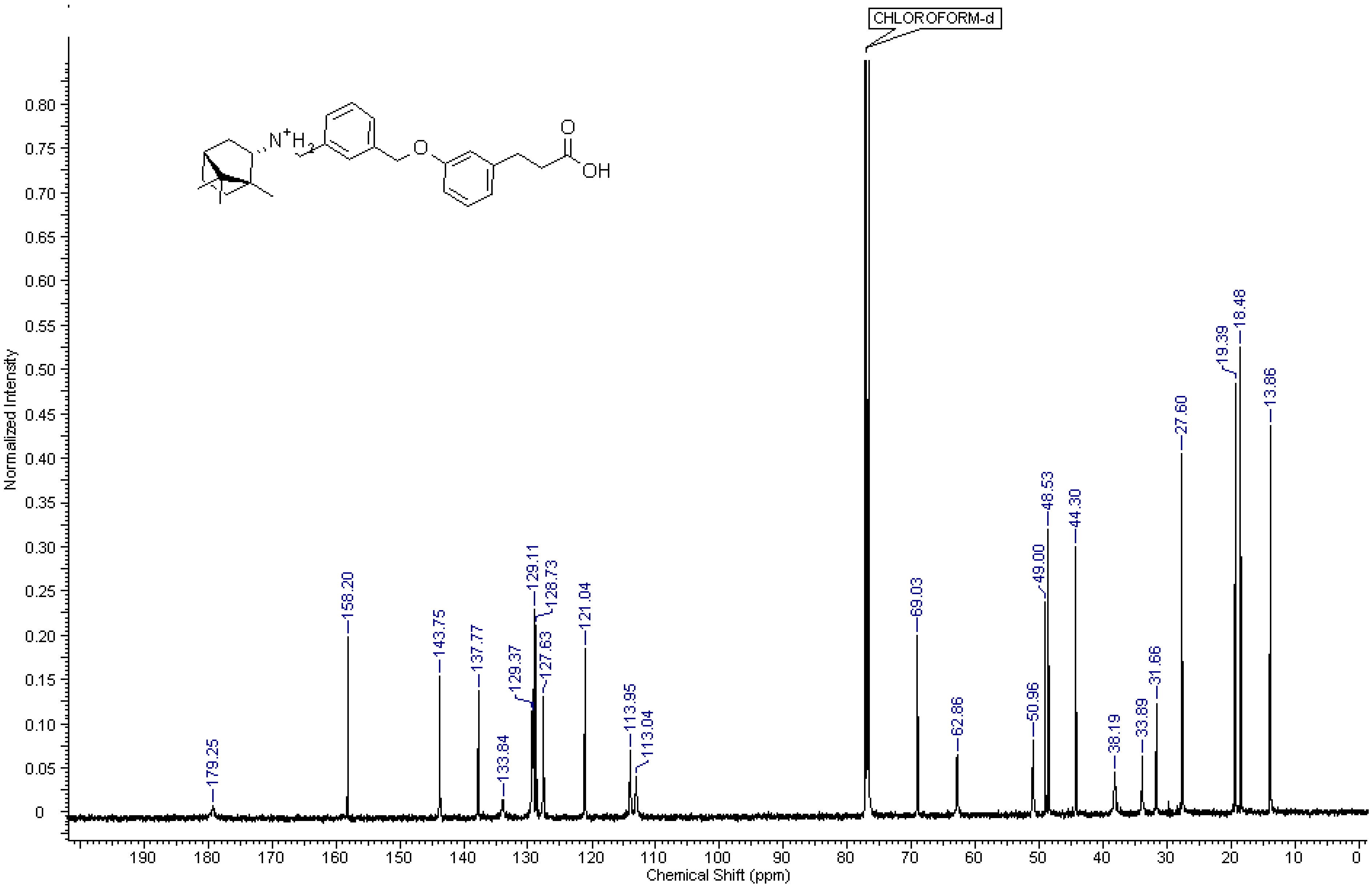

Figure S62. [13]**C NMR spectrum of 6h.**

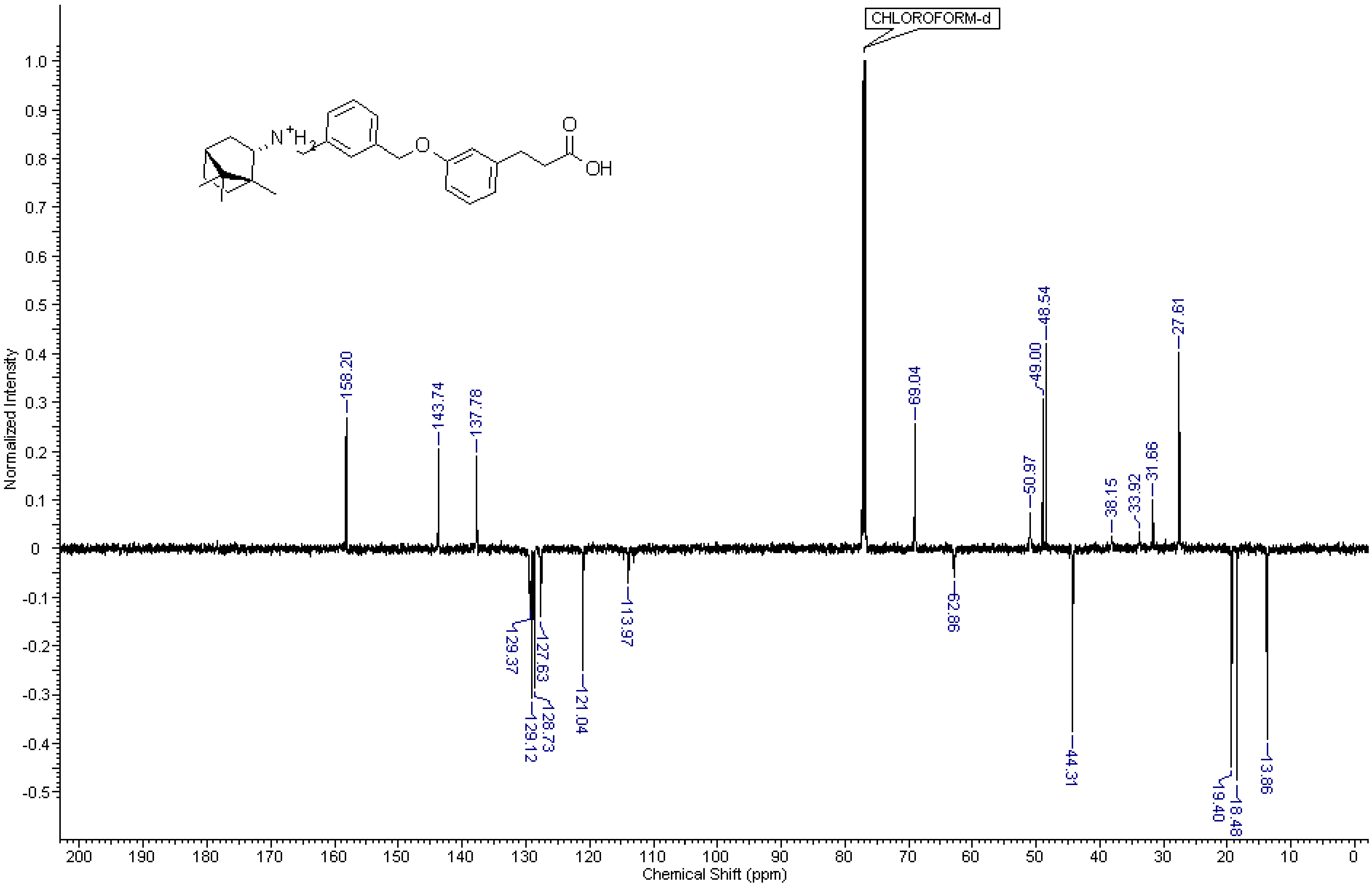

Figure S63. ^13^C NMR spectrum of 6h.
